# Supplementary material for: Embedding the intrinsic relevance of vertices in network analysis: the case of centrality metrics
Source: Sci Rep. 2020 Feb 24;10:3297. doi: 10.1038/s41598-020-60151-x (PMC7039870; doi:10.1038/s41598-020-60151-x)
Supplement: Supplementary file 1 — Supplementary Material. [file 41598_2020_60151_MOESM1_ESM.docx]

**Embedding the intrinsic relevance of vertices in network analysis: the case of centrality metrics**

^1^Orazio Giustolisi*, ^2^Luca Ridolfi, ^3^Antonietta Simone

^1^Politecnico di Bari, via Orabona, 4, Bari, Italy, orazio.giustolisi@poliba.it

^2^Politecnico di Torino, Corso Duca degli Abruzzi, 24, Torino, Italy

^3^Politecnico di Bari, via Orabona, 4, Bari, Italy

**Supplementary Material**

**Supplementary Figures 1A- 24B**

The following figures report the application of the vertices intrinsic relevance to two regular networks with 100 and 1,000 vertices and two random networks of the same size, both generated using the Watts and Strogatz model (1998) with mean degree *d* = 10 and probability p = {0,1}. The standard betweenness and harmonic centrality were computed for each network, while the intrinsic relevance-based metrics were computed using each one of the six functions *f*(*R_s_*, *R_t_*) reported in the manuscript. The intrinsic relevance was assigned to vertices adding to the “classic” unit value a random one sampled from the uniform distribution in the range [0; *d*]. At first a fraction, *r,* of 10 % of vertices was randomly selected and the intrinsic relevance was randomly assigned. Then, the intrinsic relevance was randomly assigned to all the vertices (*r*= 100%) in the same range [1; 1+*d*].

Therefore, for each network (regular and random, with 100 and 1,000 vertices), function *f*(*R_s_*, *R_t_*) and pattern of intrinsic relevance (10% and 100% of randomly assigned intrinsic relevance to vertices) the figures report the scatter plot of the:

- standard *versus* the relevance-based betweenness;
- relevance-based betweenness *versus* intrinsic relevance;
- standard *versus* the relevance-based harmonic centrality;
- relevance-based harmonic centrality *versus* intrinsic relevance;

and five networks (for each case of intrinsic relevance random assignment) with coloured vertices referring to a colour-bar indicating the values of the:

- standard betweenness;
- relevance-based betweenness;
- intrinsic relevance of vertices;
- standard harmonic centrality;
- relevance-based harmonic centrality.


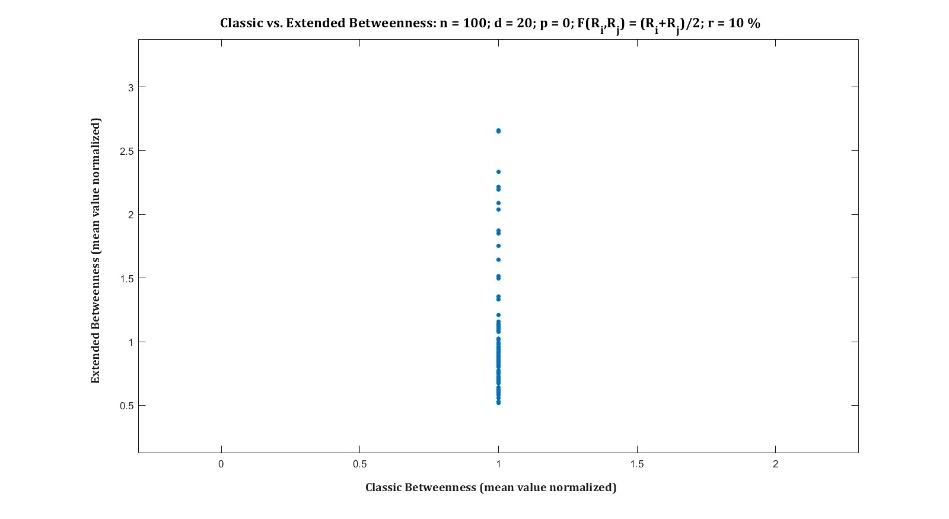

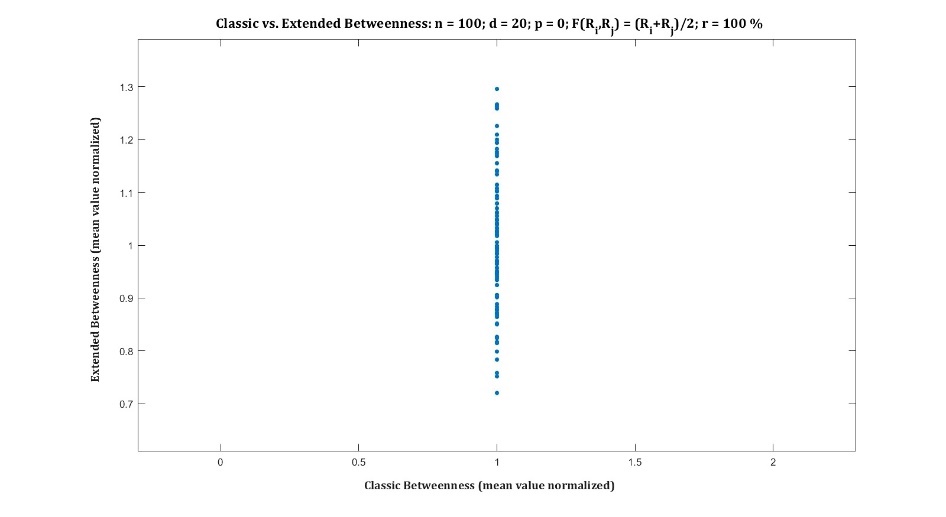


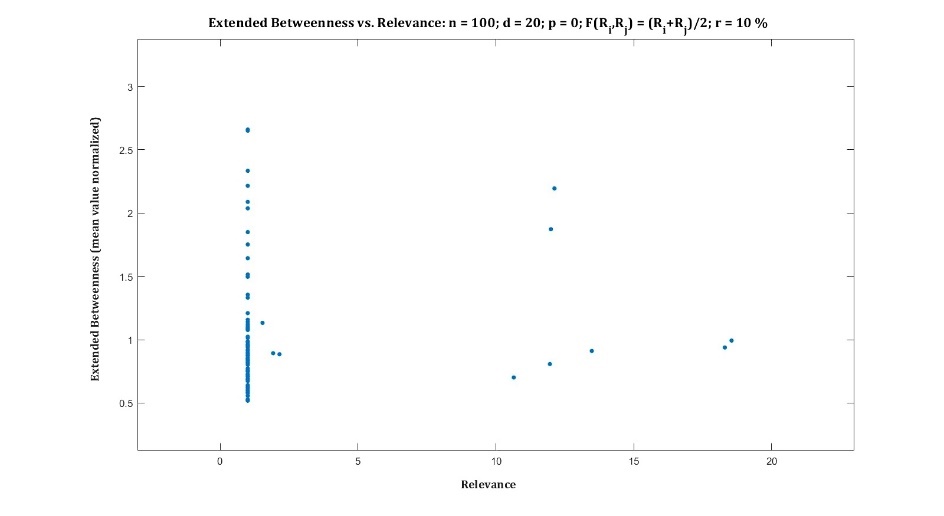

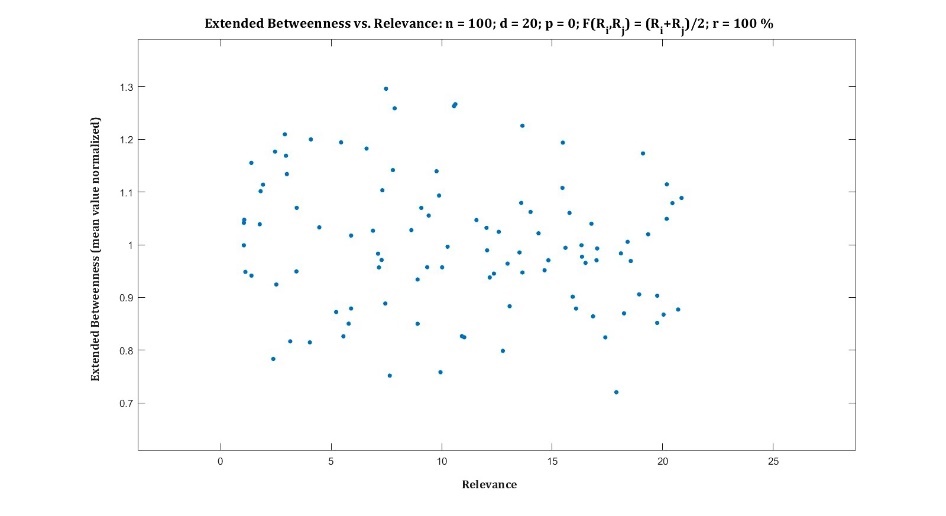


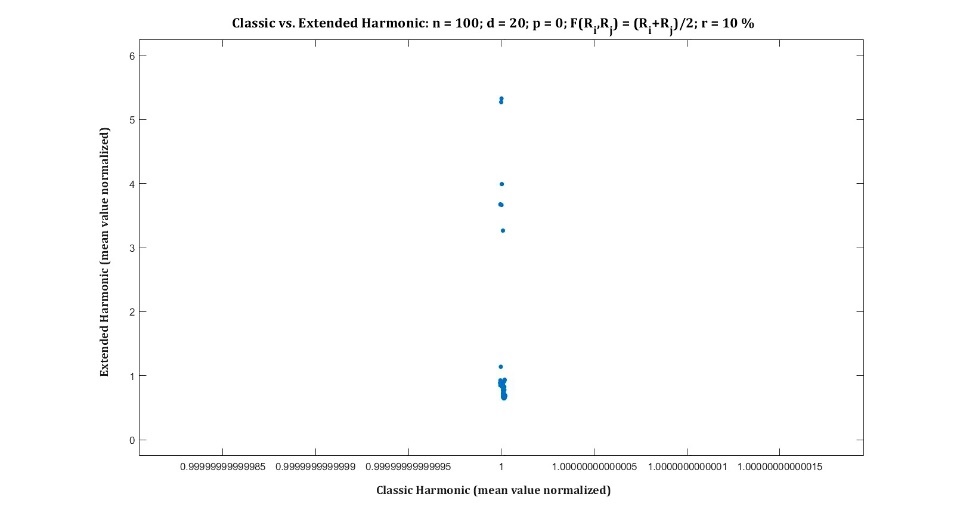

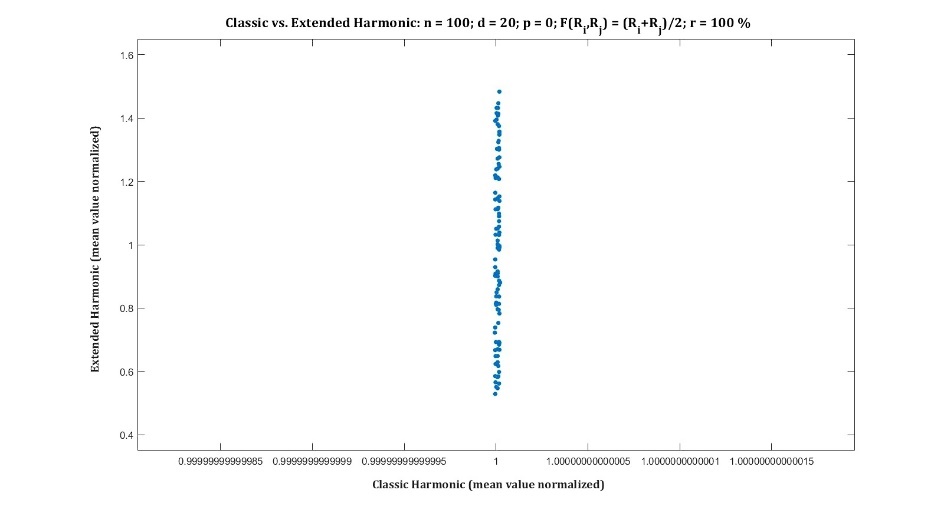


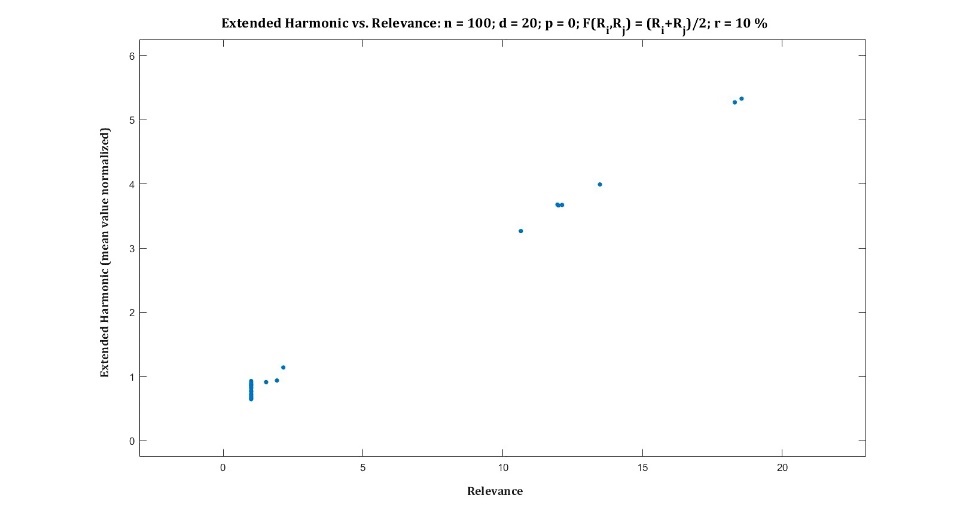

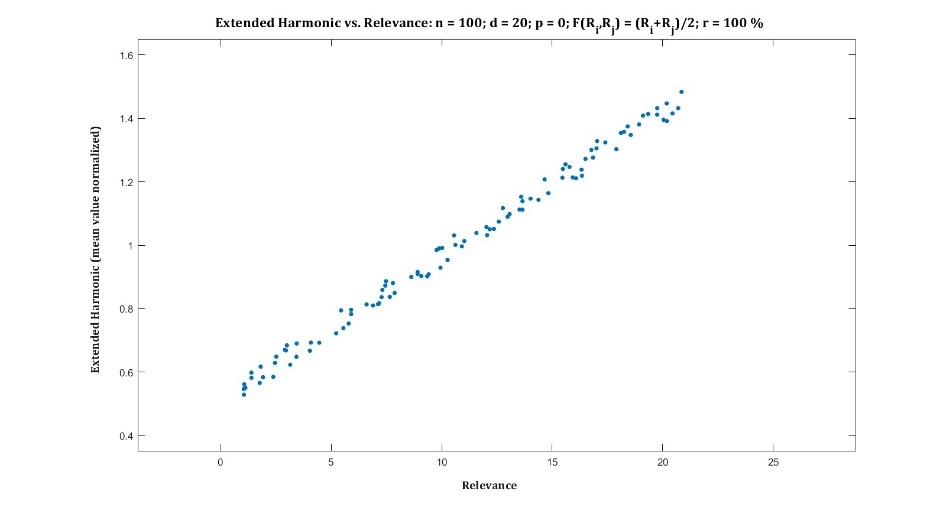


Figure 1A: *f*(*R_i_*, *R_j_*) = (*R_i_*+*R_j_*)/2 performed for a regular network with 100 vertices. It reports: standard *vs* relevance-based betweenness with *r*=10% (1^st^ panel-left) and *r*=100% (1^st^ panel-right), intrinsic relevance *vs* relevance-based betweenness with *r* =10% (2^nd^ panel-left) and *r* =100% (2^nd^ panel-right), classic *vs* relevance-based harmonic centrality with *r* =10% (3^rd^ panel-left) and *r* =100% (3^rd^ panel-right), and intrinsic relevance *vs* relevance-based harmonic with *r* =10% (4^th^ panel-left) and *r* =100% (4^th^ panel-right).


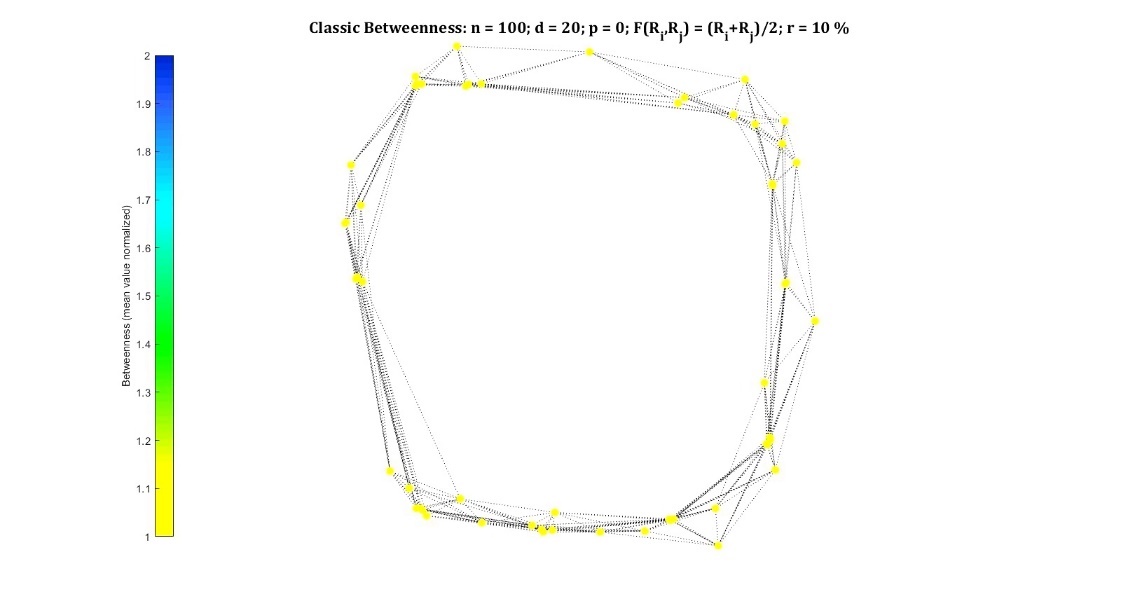

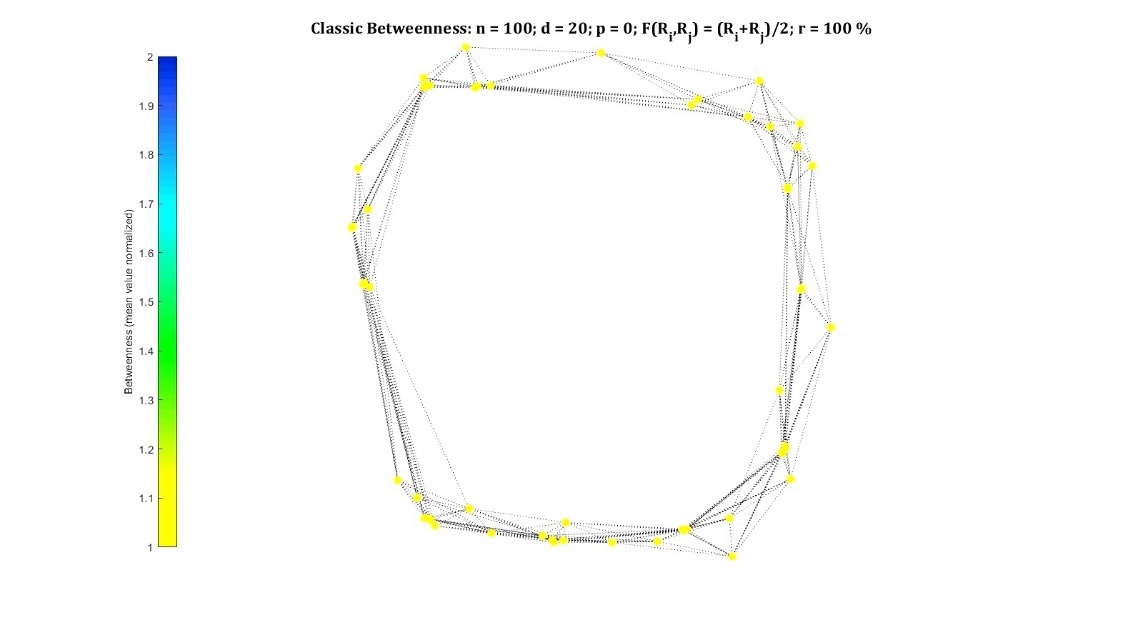


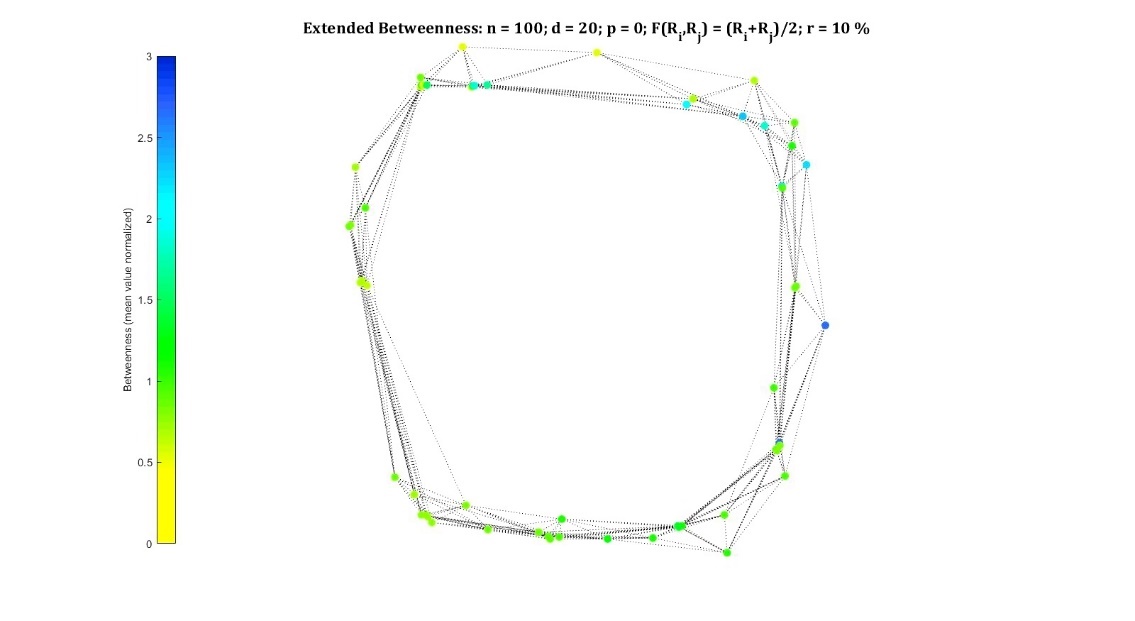

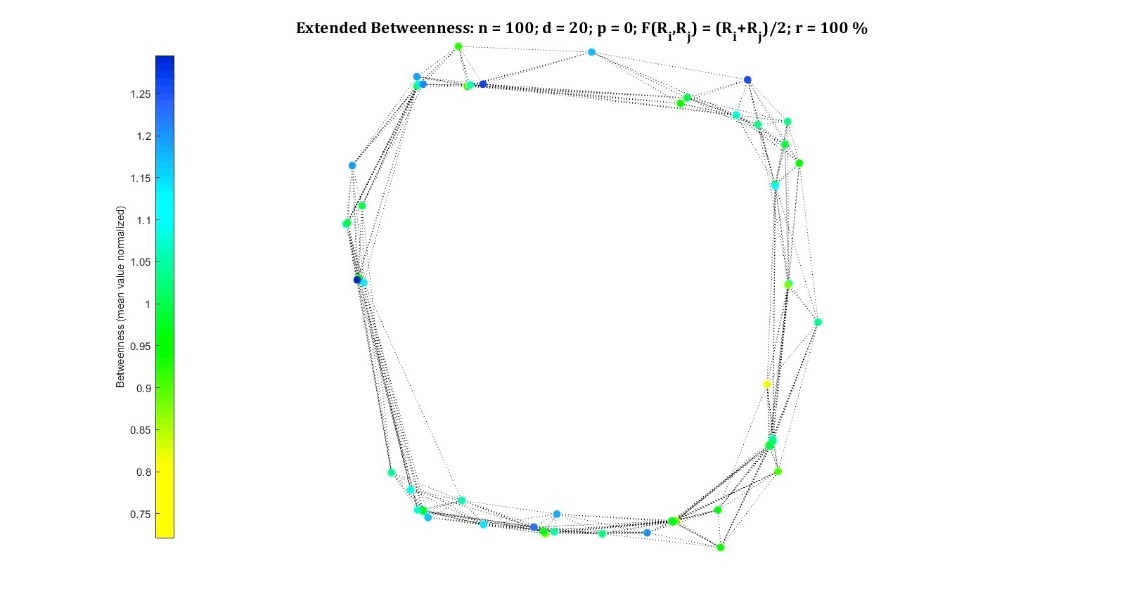


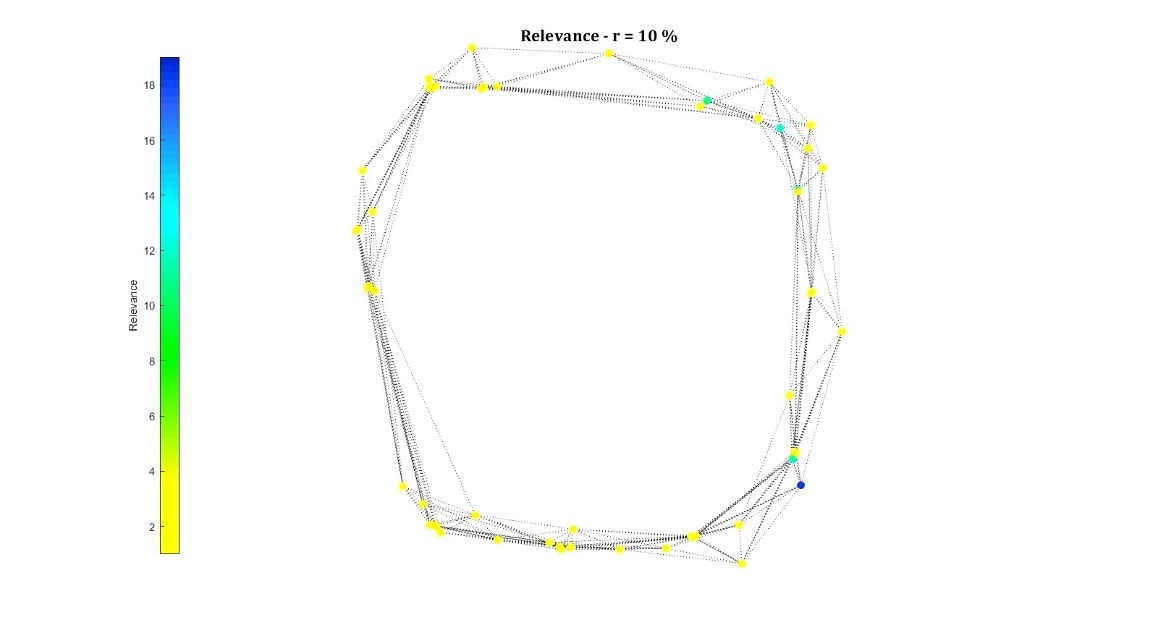

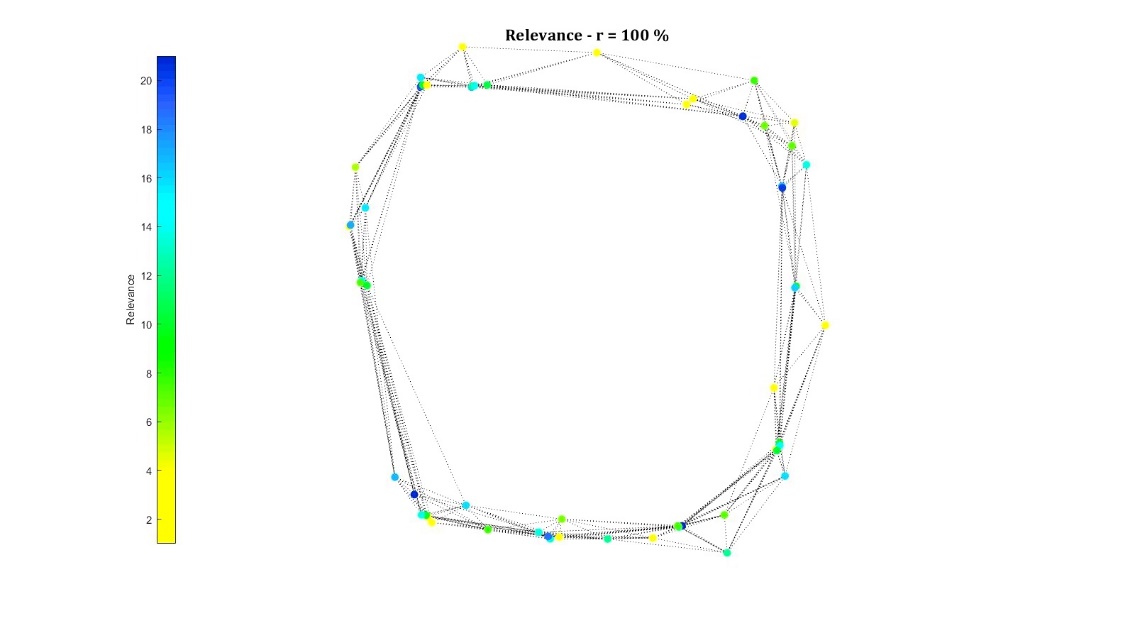


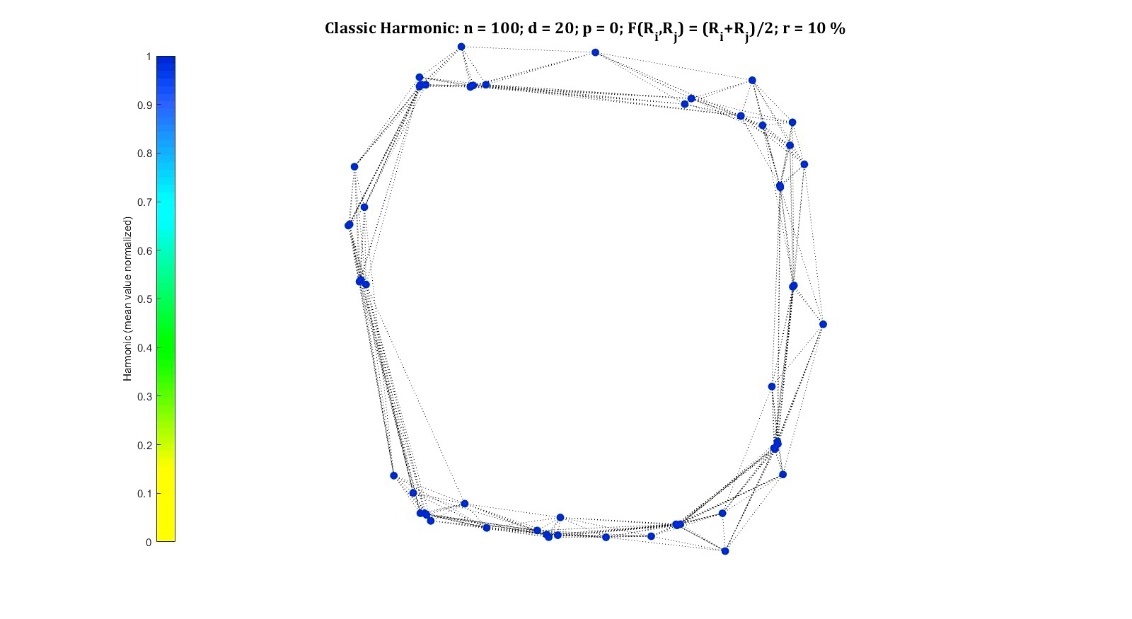

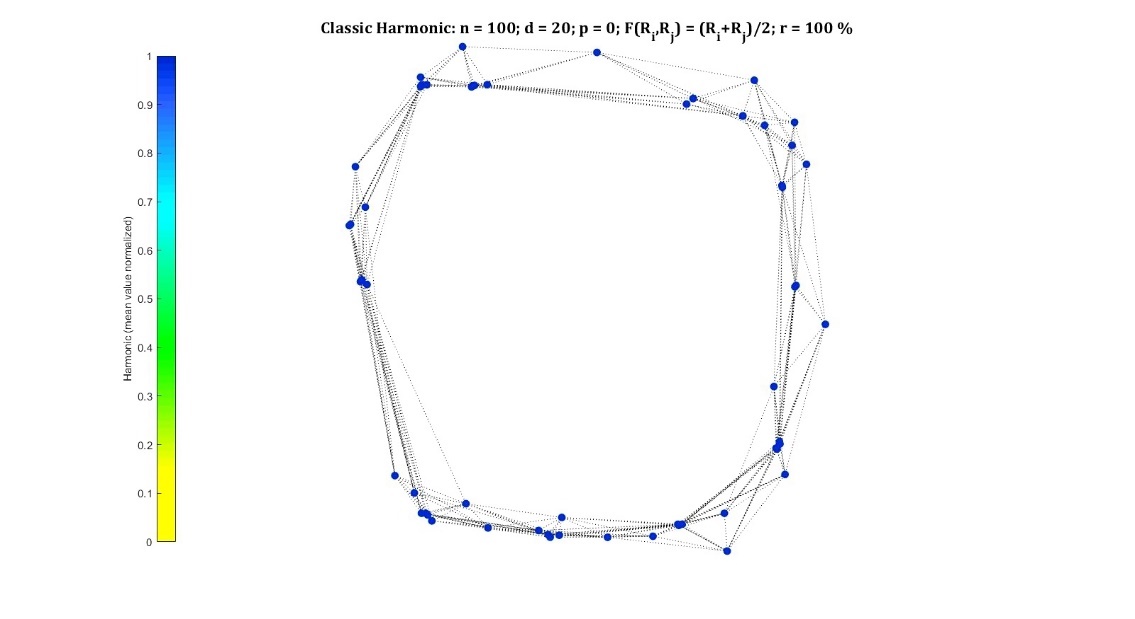


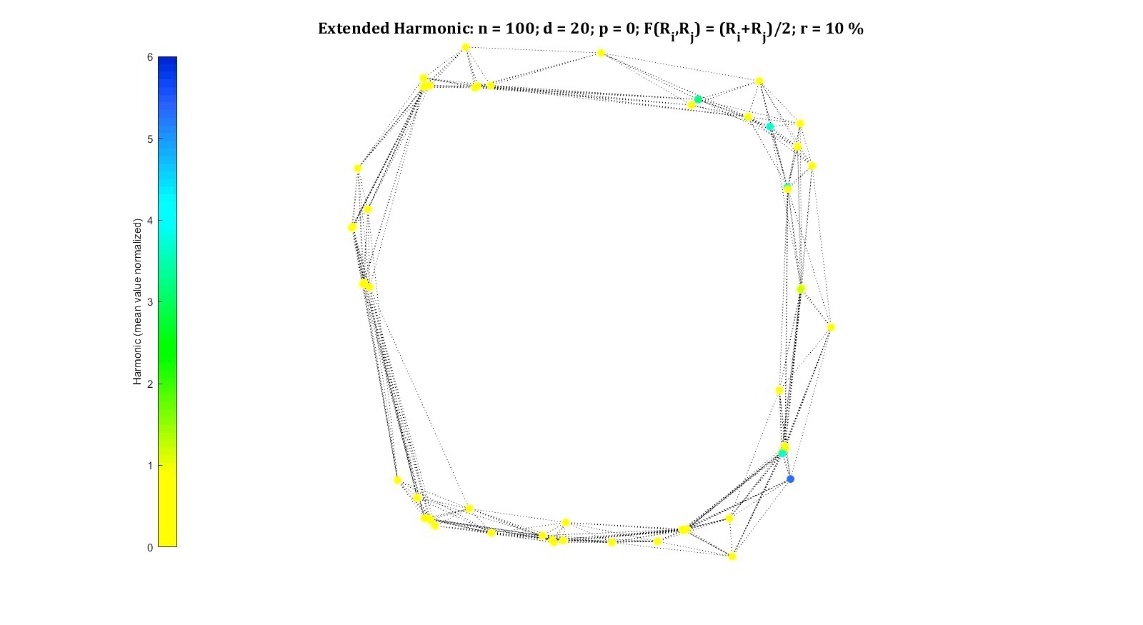

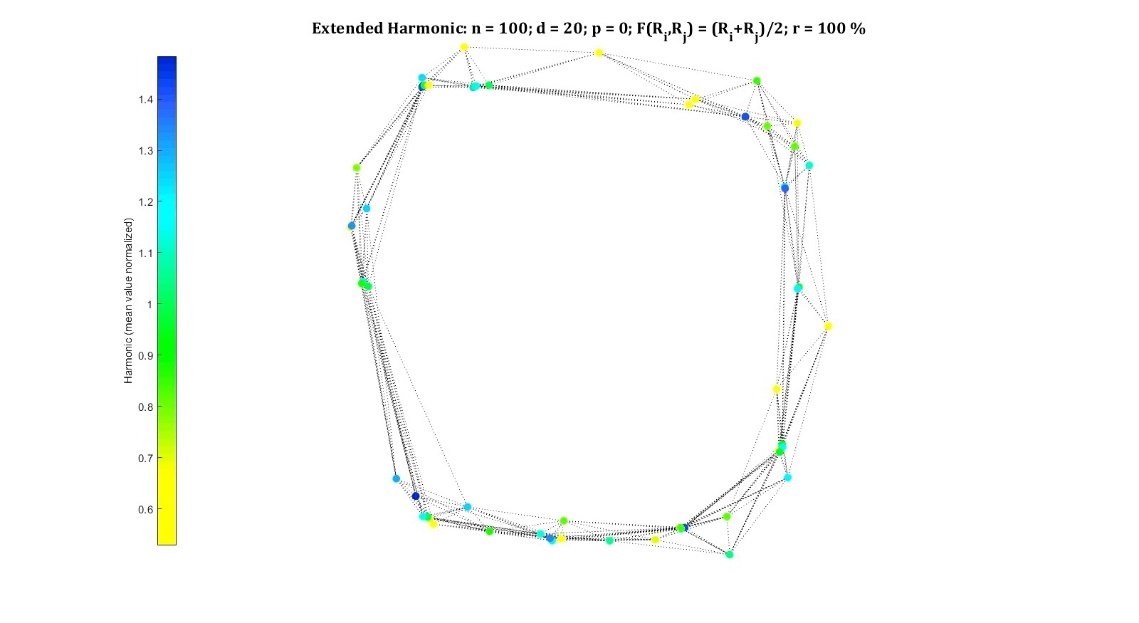


Figure 1B: regular network with 100 vertices corresponding to *r*=10% (left-panel) and *r*=100% (right-panel) of the randomly assigned intrinsic relevance. The coloured vertices refer to the colour-bar indicating the values of the: standard betweenness (1^st^ panel), relevance-based betweenness (2^nd^ panel), intrinsic relevance of vertices (3^rd^ panel), standard harmonic centrality (4^th^ panel) and relevance-based harmonic centrality (5^th^ panel). The relevance-based metrics refers to the function *f*(*R_i_*, *R_j_*) = (*R_i_*+*R_j_*)/2 as in Figure 1A.


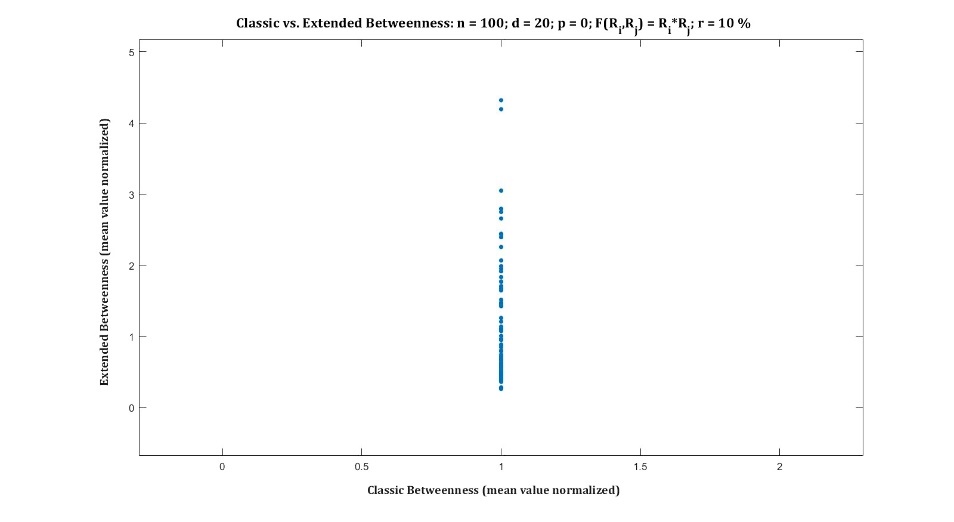

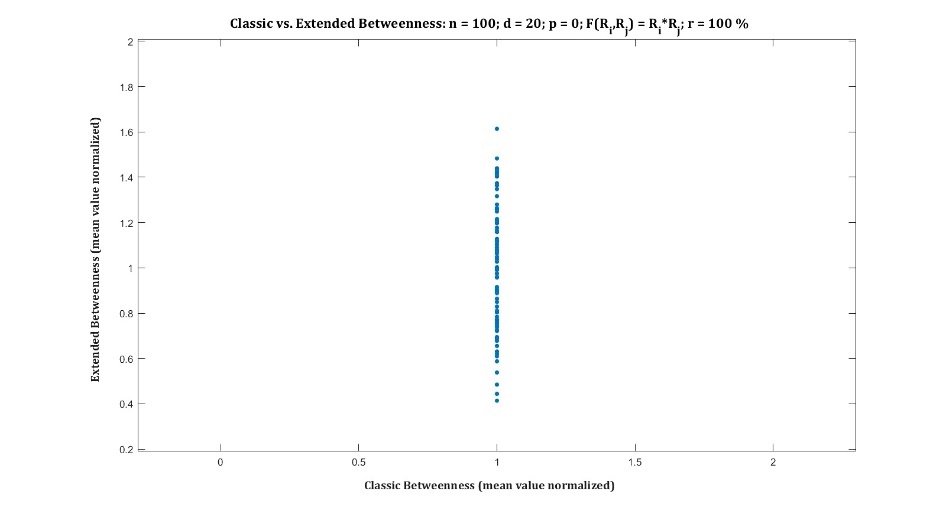


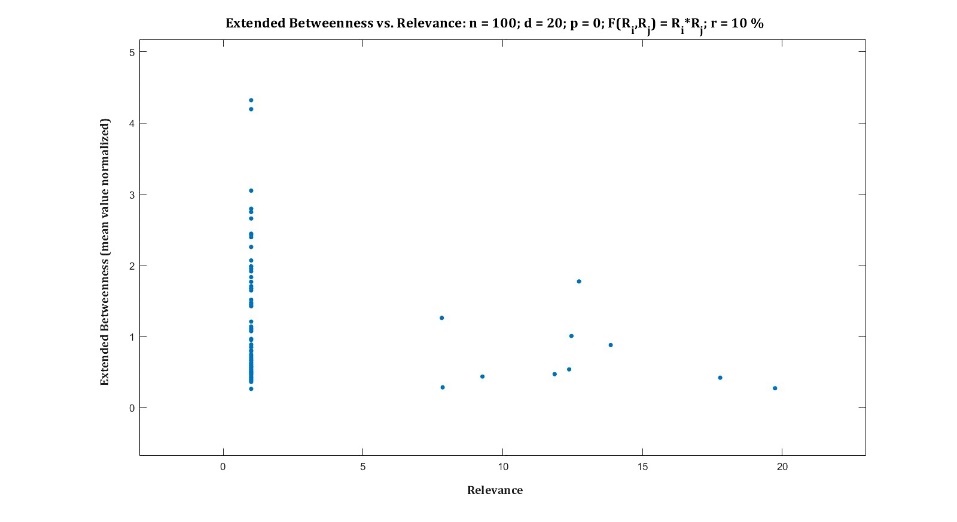

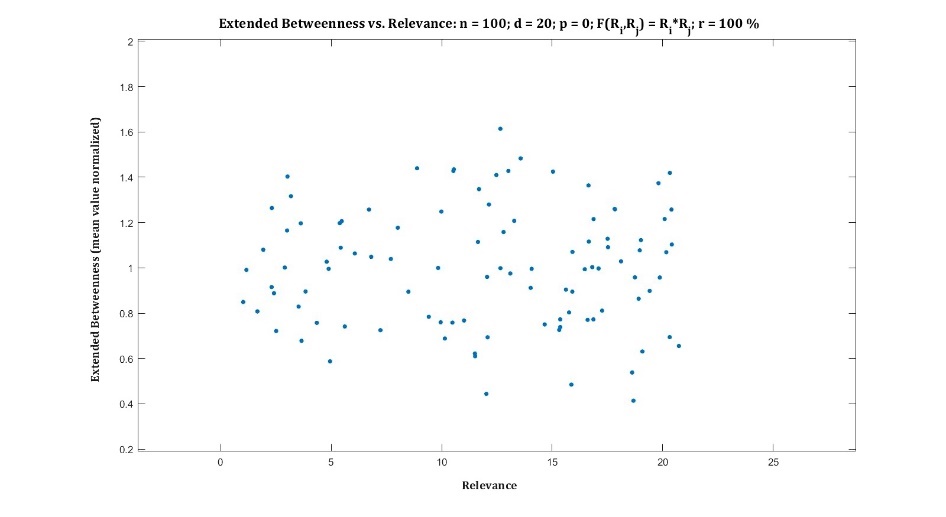


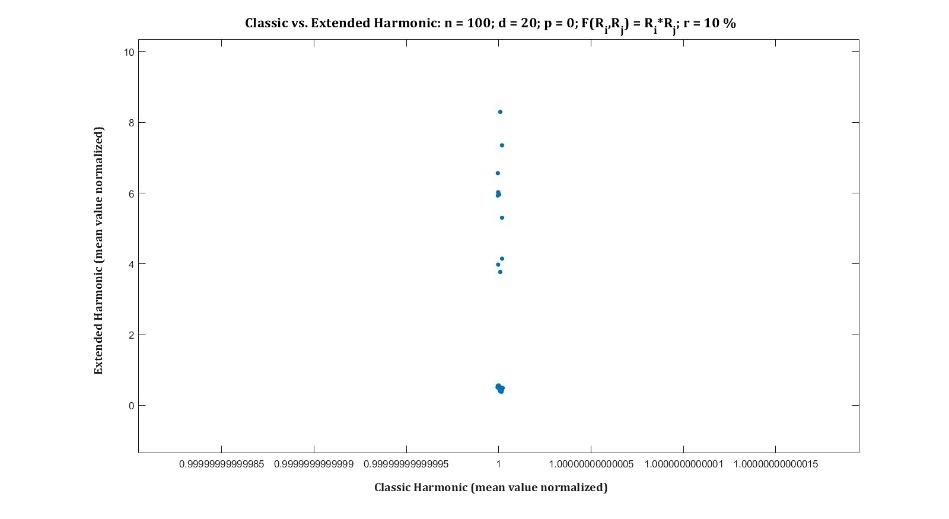

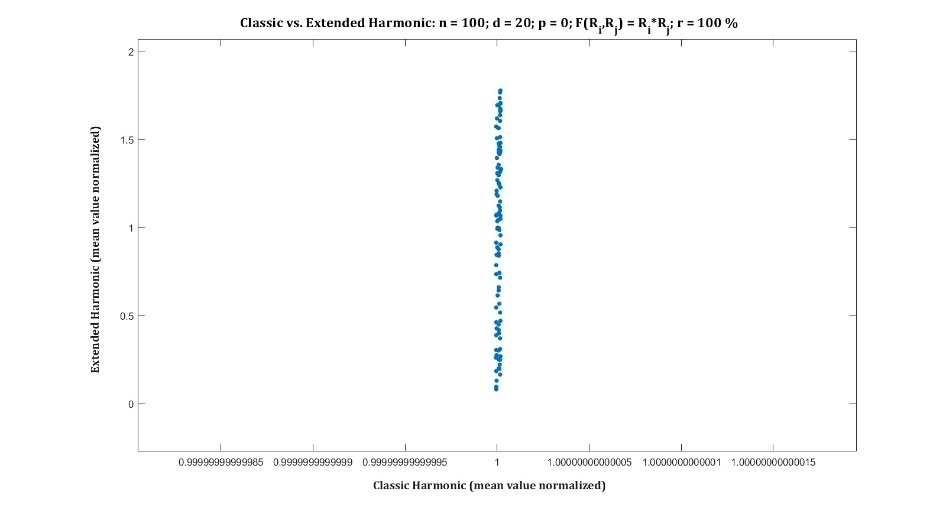


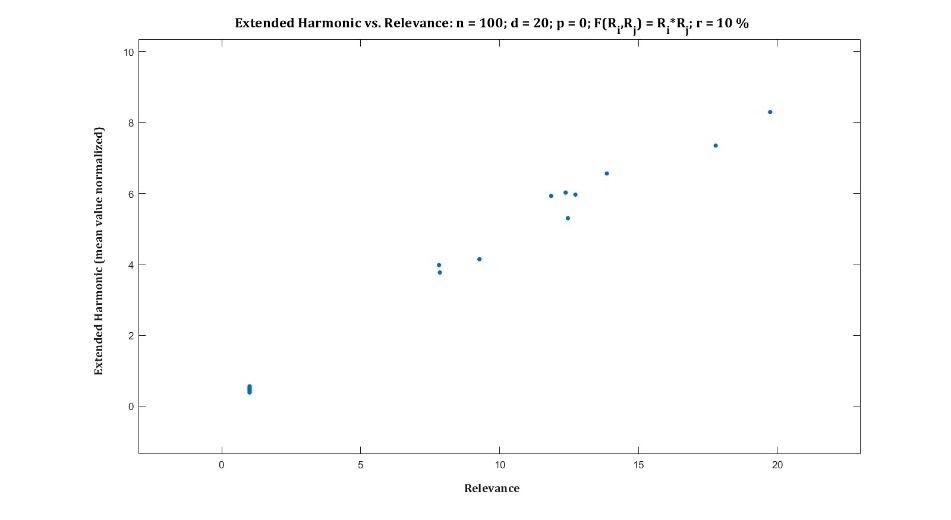

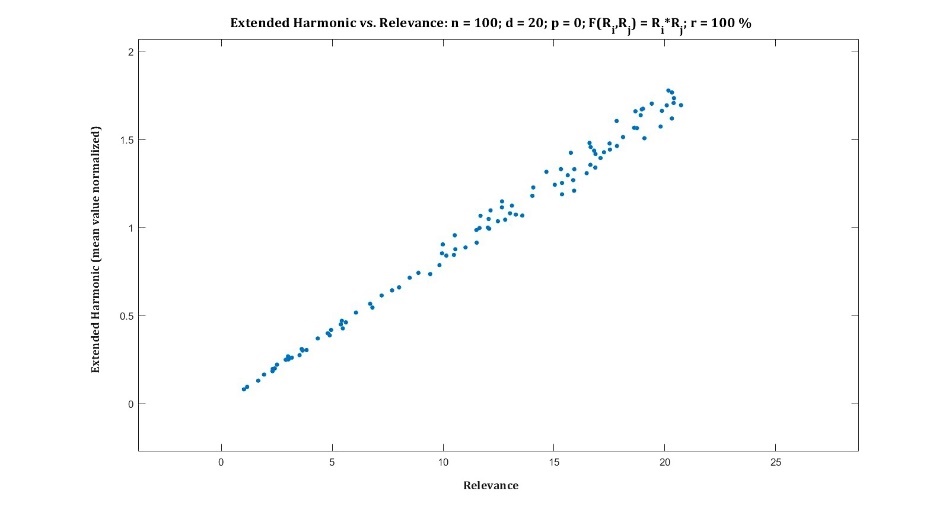


Figure 2A: *f*(*R_i_*, *R_j_*) = *R_i_*∙*R_j_* performed for a regular network with 100 vertices. It reports: standard *vs* relevance-based betweenness with *r*=10% (1^st^ panel-left) and *r*=100% (1^st^ panel-right), intrinsic relevance *vs* relevance-based betweenness with *r* =10% (2^nd^ panel-left) and *r* =100% (2^nd^ panel-right), classic *vs* relevance-based harmonic centrality with *r* =10% (3^rd^ panel-left) and *r* =100% (3^rd^ panel-right), and intrinsic relevance *vs* relevance-based harmonic with *r* =10% (4^th^ panel-left) and *r* =100% (4^th^ panel-right).


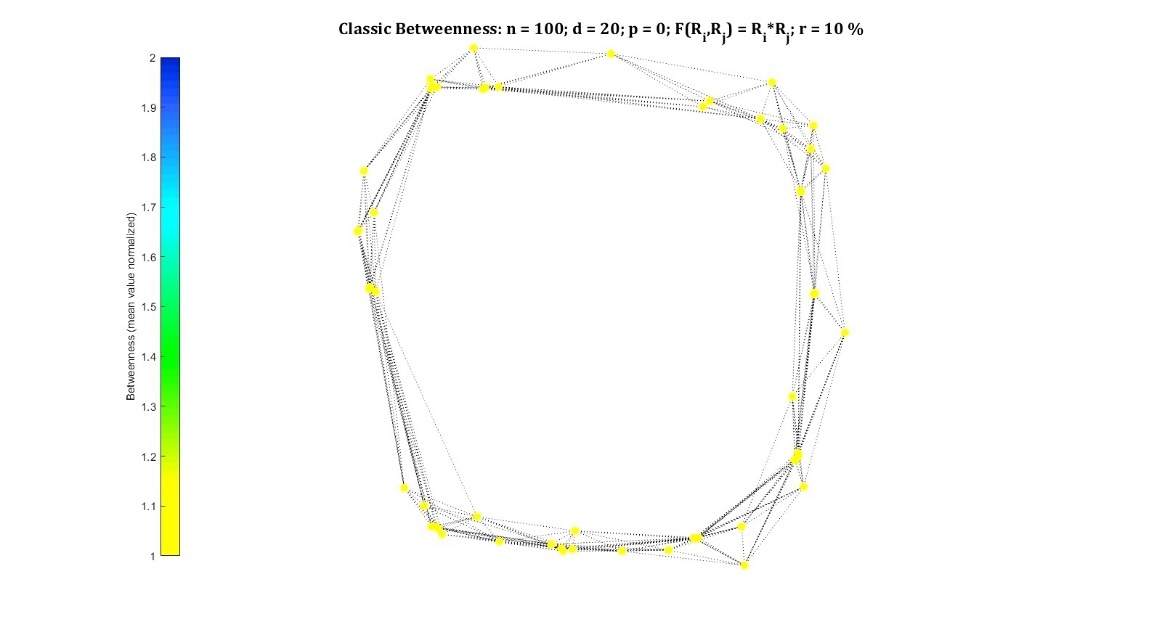

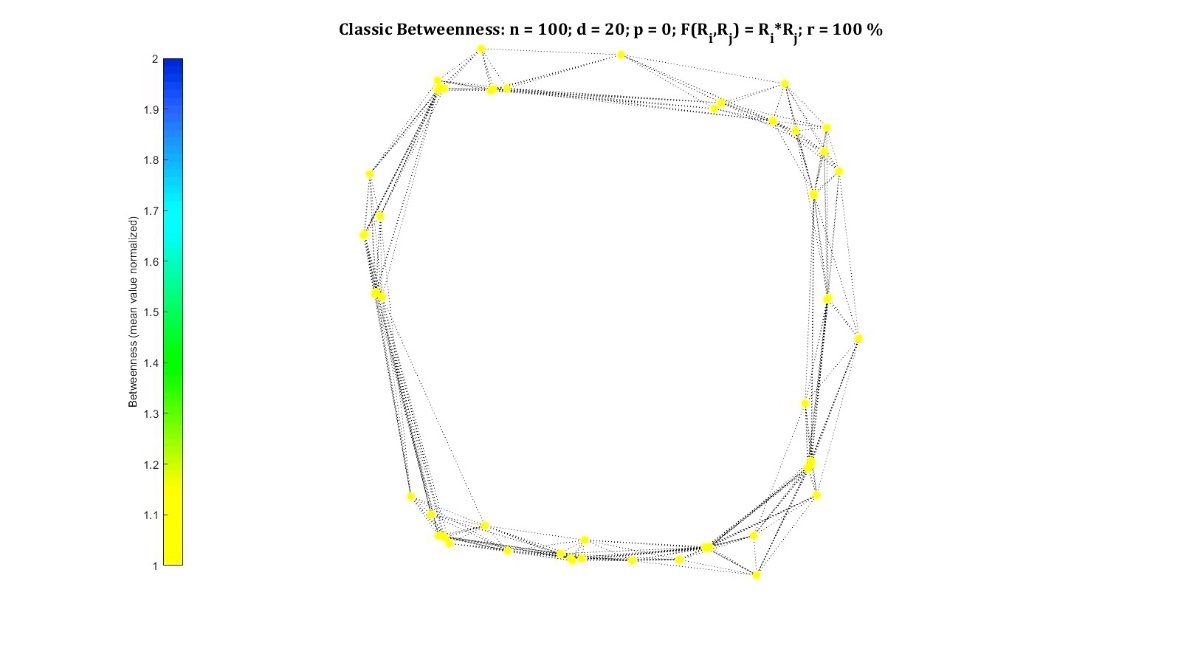


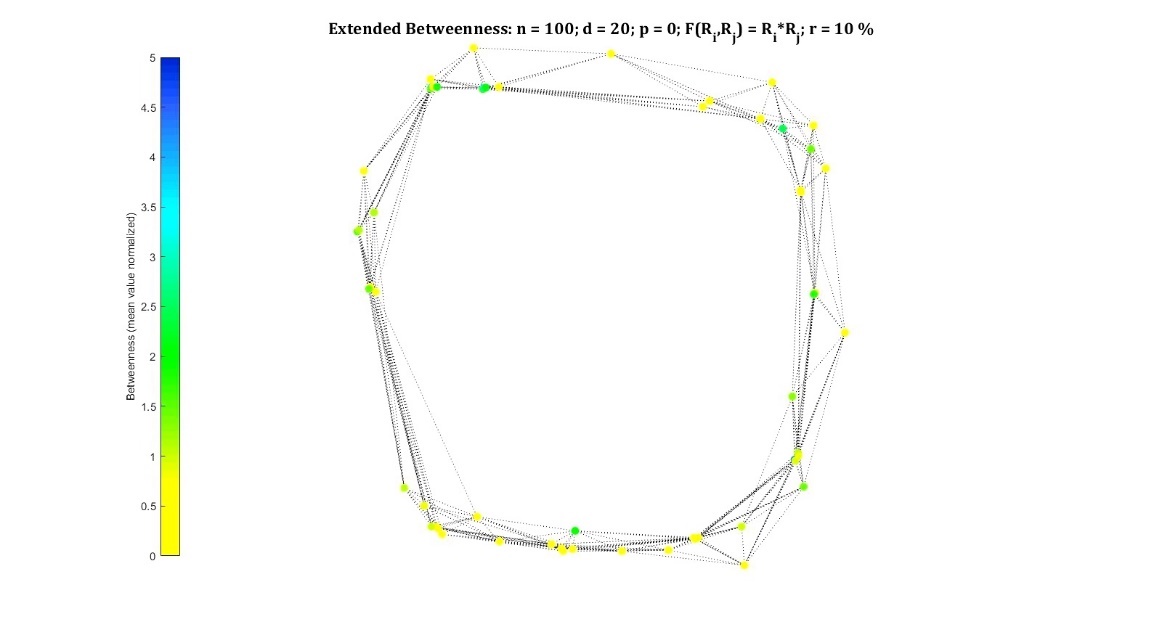

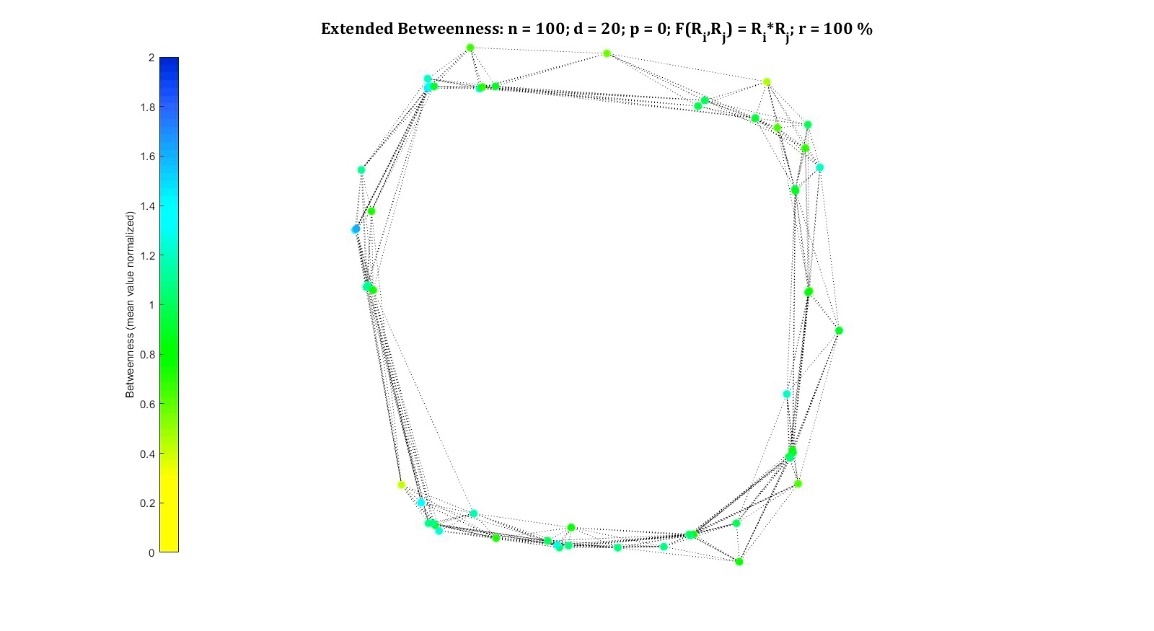


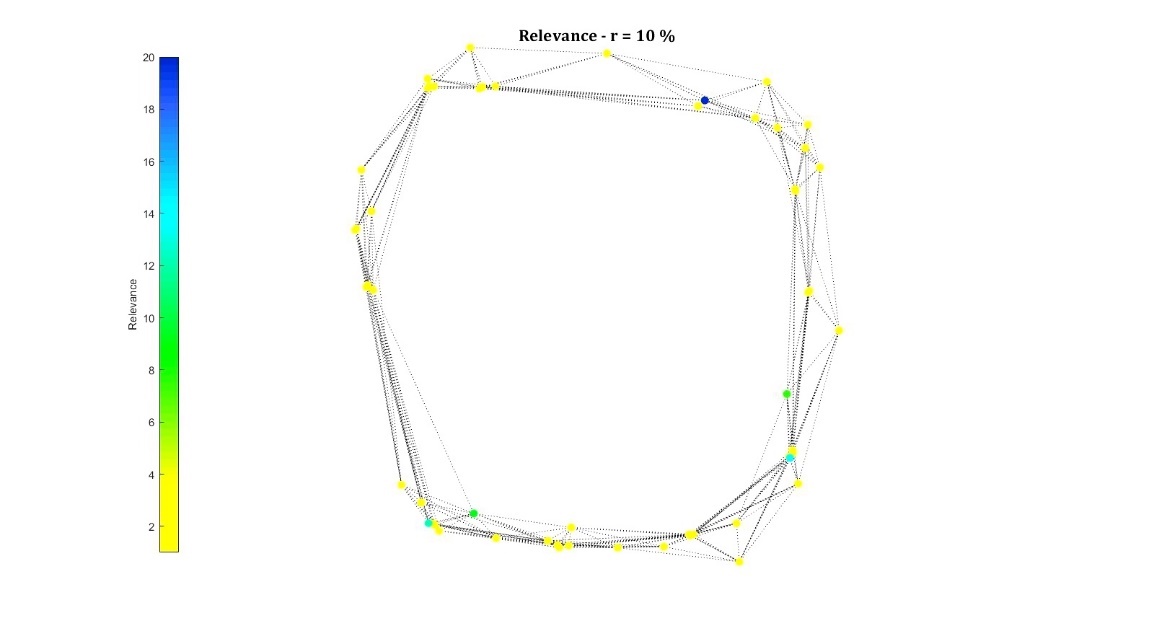

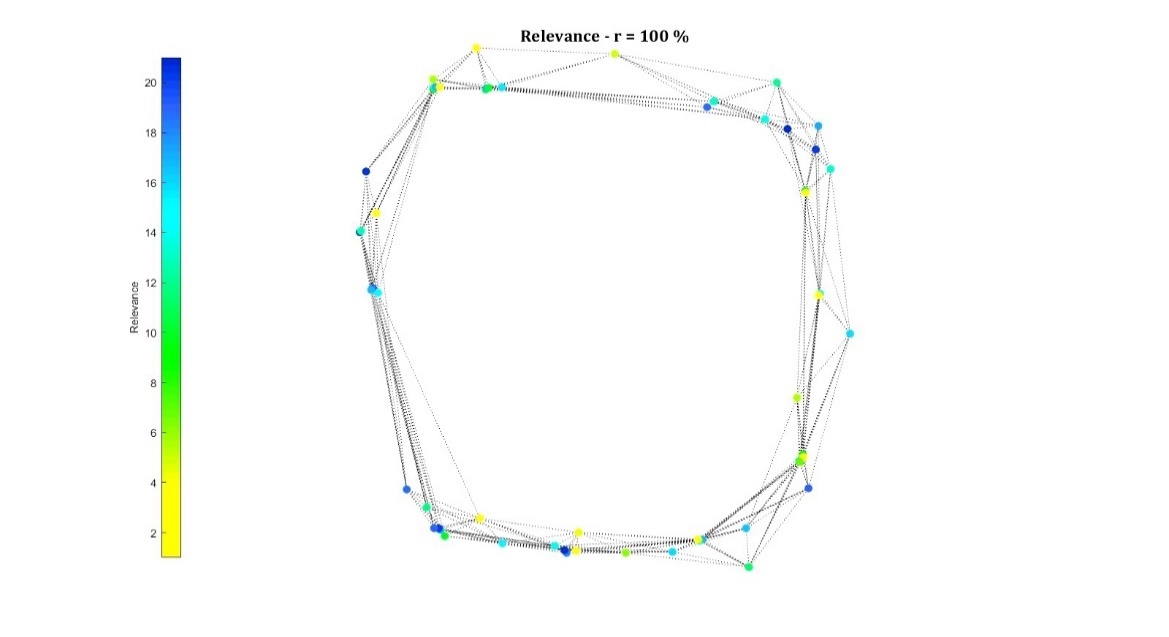


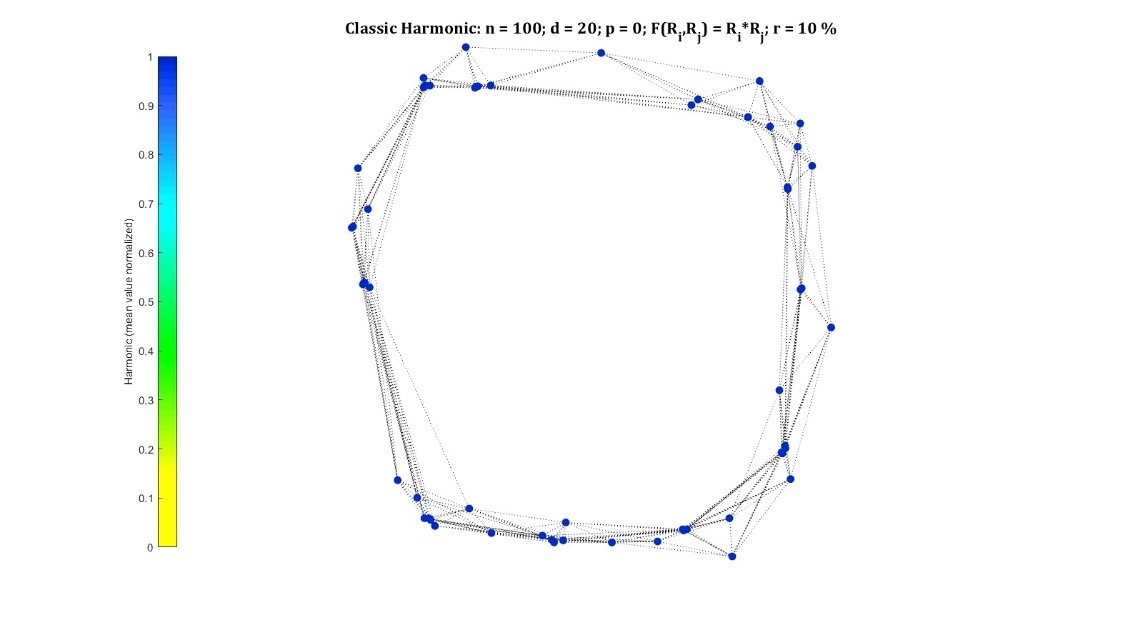

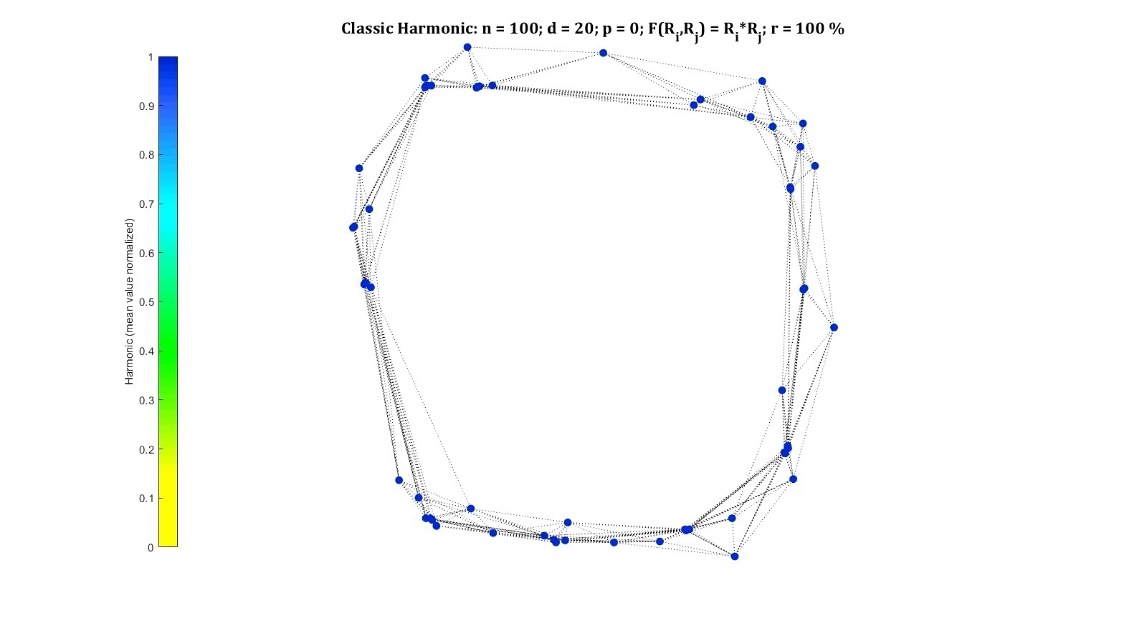


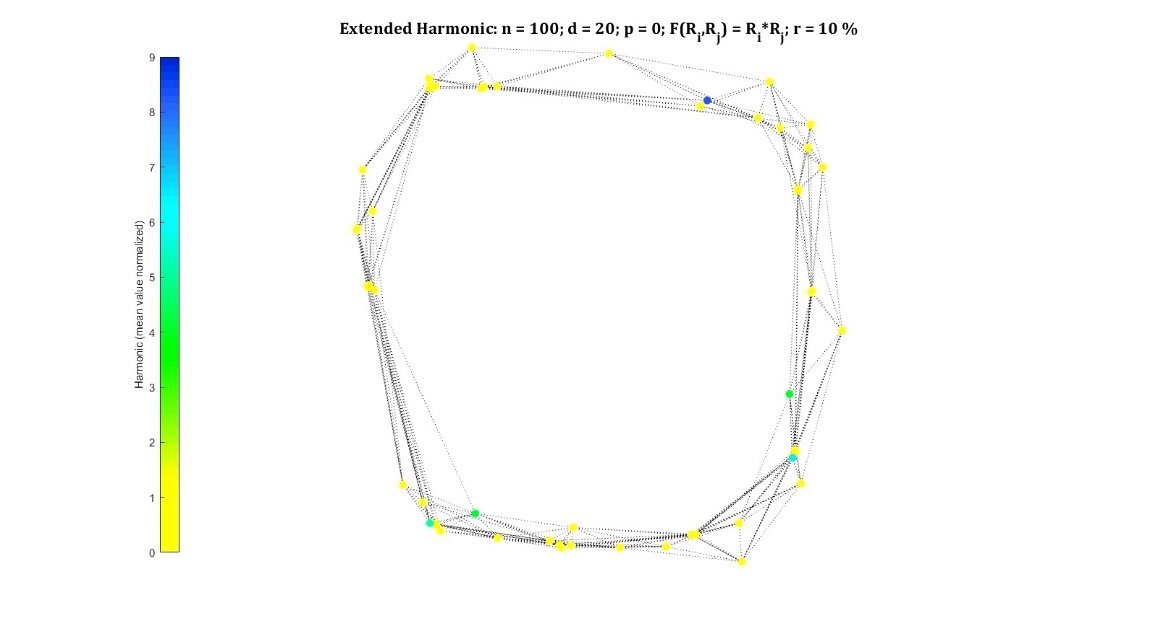

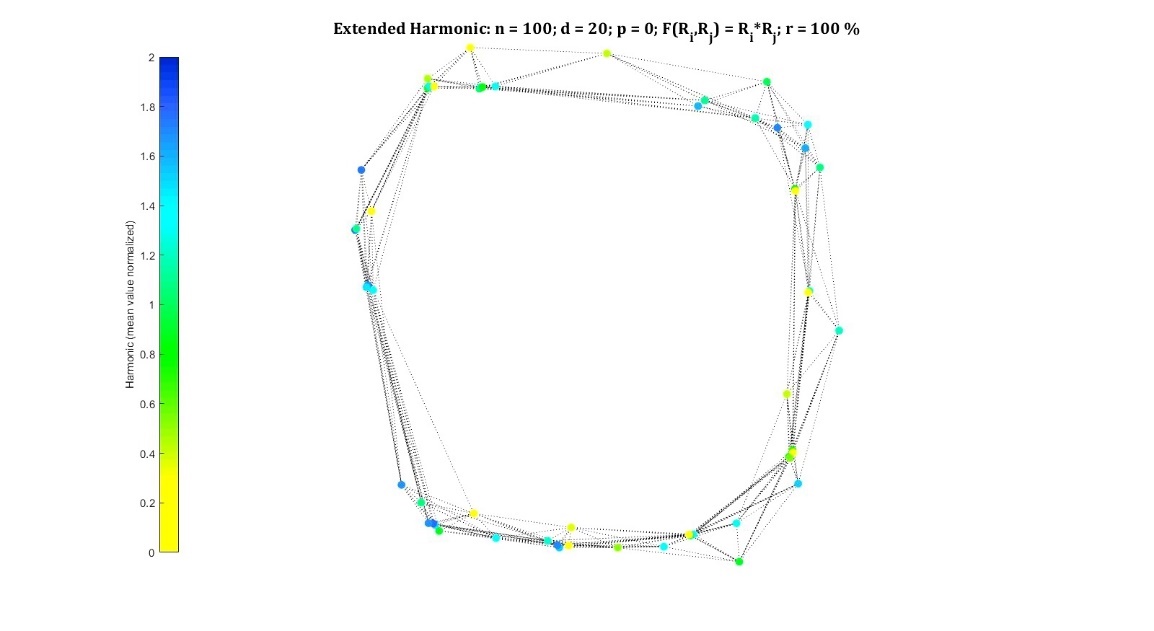


Figure 2B: regular network with 100 vertices corresponding to *r*=10% (left-panel) and *r*=100% (right-panel) of the randomly assigned intrinsic relevance. The coloured vertices refer to the colour-bar indicating the values of the: standard betweenness (1^st^ panel), relevance-based betweenness (2^nd^ panel), intrinsic relevance of vertices (3^rd^ panel), standard harmonic centrality (4^th^ panel) and relevance-based harmonic centrality (5^th^ panel). The relevance-based metrics refers to the function *f*(*R_i_*, *R_j_*) = *R_i_*∙*R_j_* as in Figure 2A.


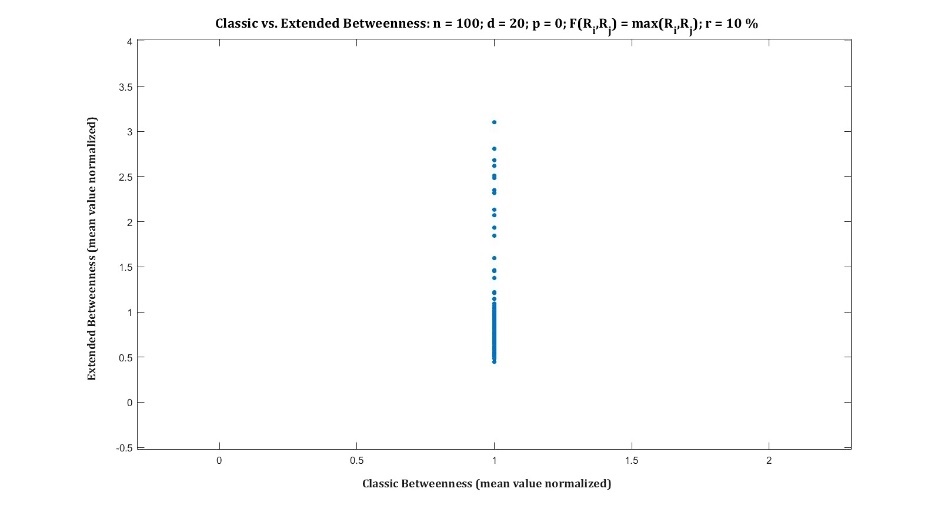

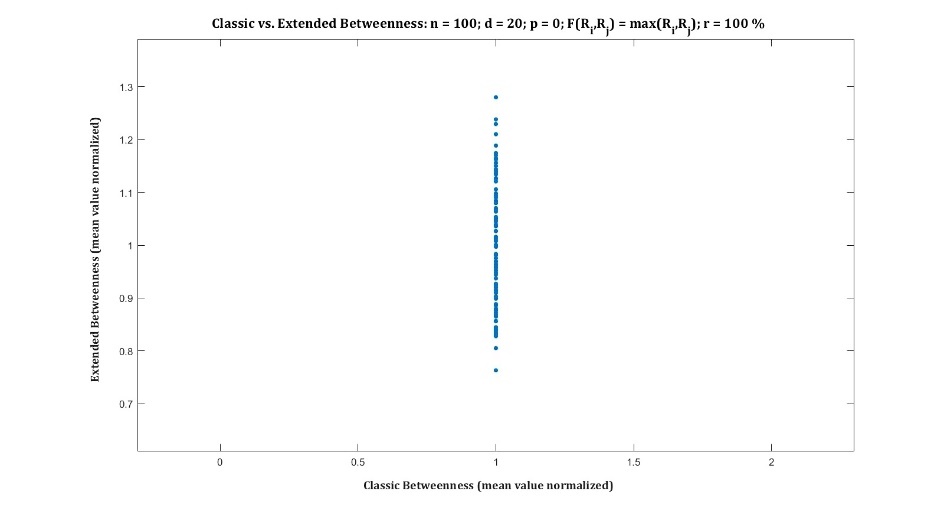


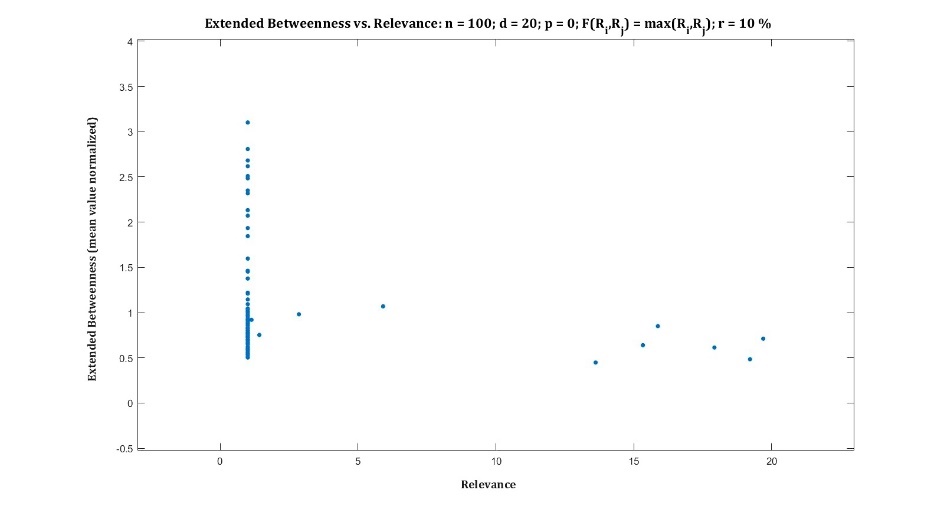

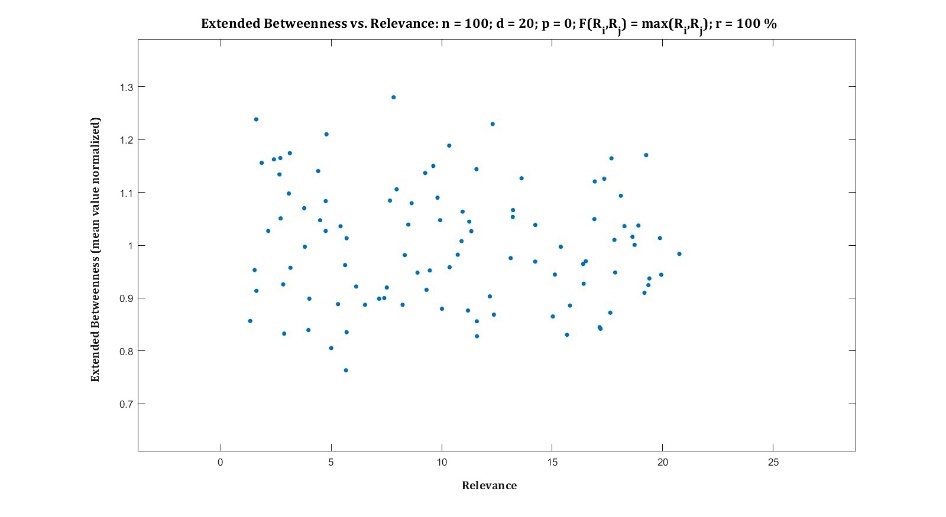


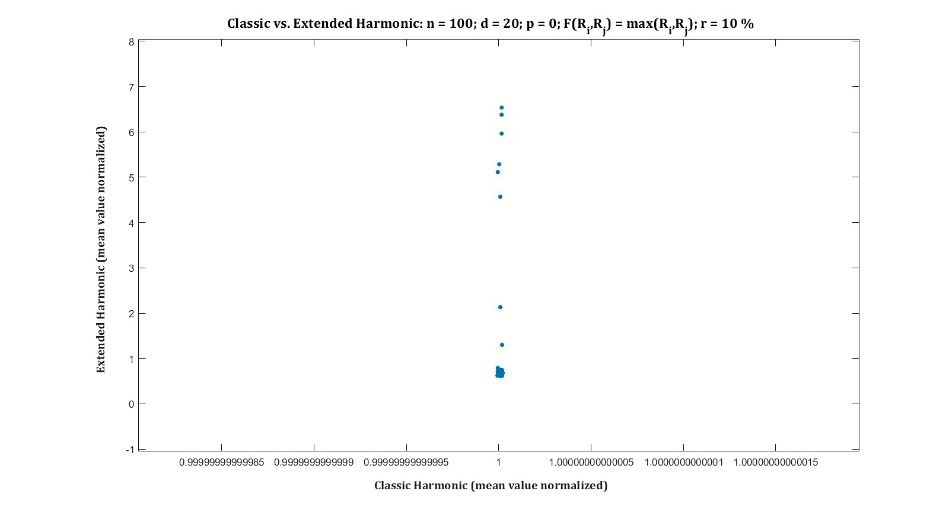

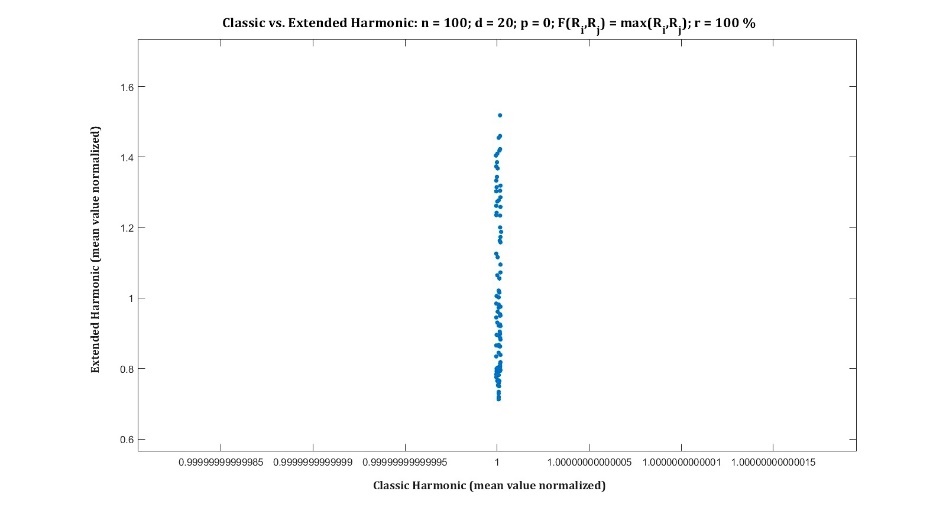


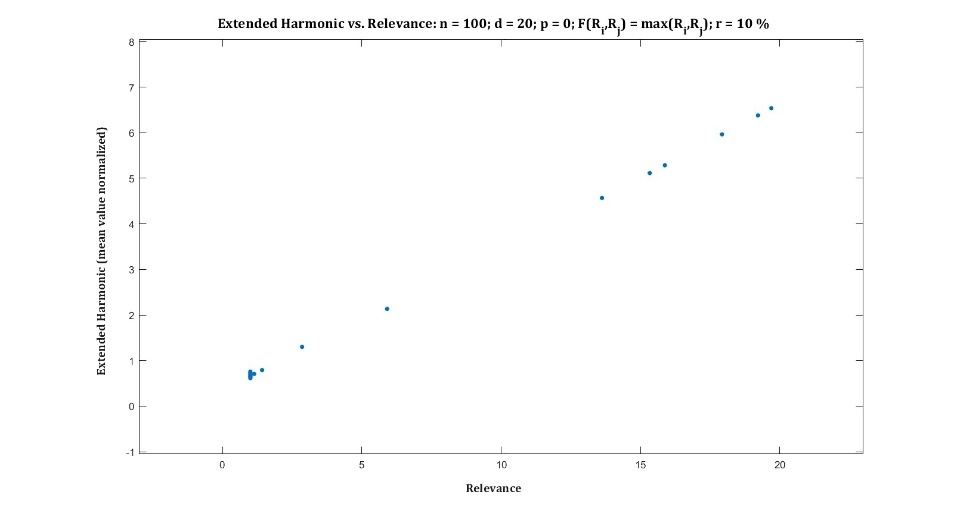

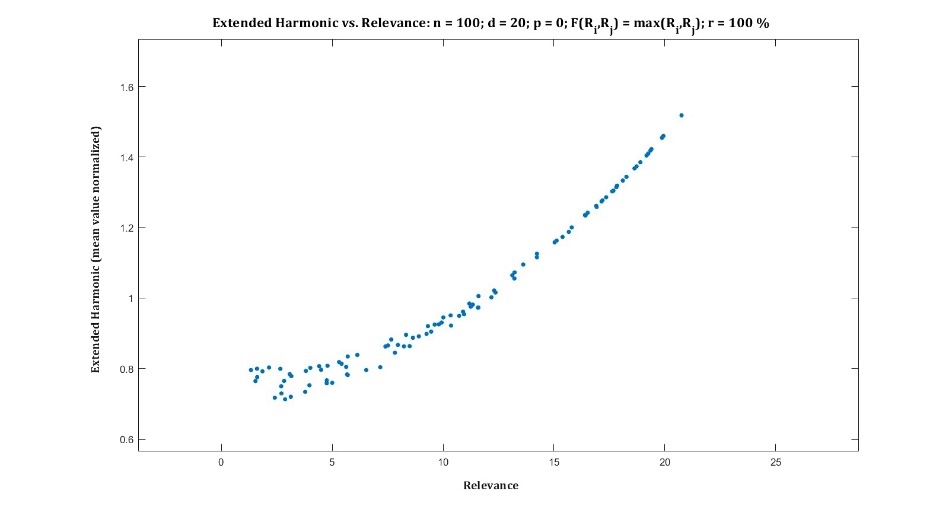


Figure 3A: *f*(*R_i_*, *R_j_*) = max(*R_i_*, *R_j_*) performed for a regular network with 100 vertices. It reports: standard *vs* relevance-based betweenness with *r*=10% (1^st^ panel-left) and *r*=100% (1^st^ panel-right), intrinsic relevance *vs* relevance-based betweenness with *r* =10% (2^nd^ panel-left) and *r* =100% (2^nd^ panel-right), classic *vs* relevance-based harmonic centrality with *r* =10% (3^rd^ panel-left) and *r* =100% (3^rd^ panel-right), and intrinsic relevance *vs* relevance-based harmonic with *r* =10% (4^th^ panel-left) and *r* =100% (4^th^ panel-right).


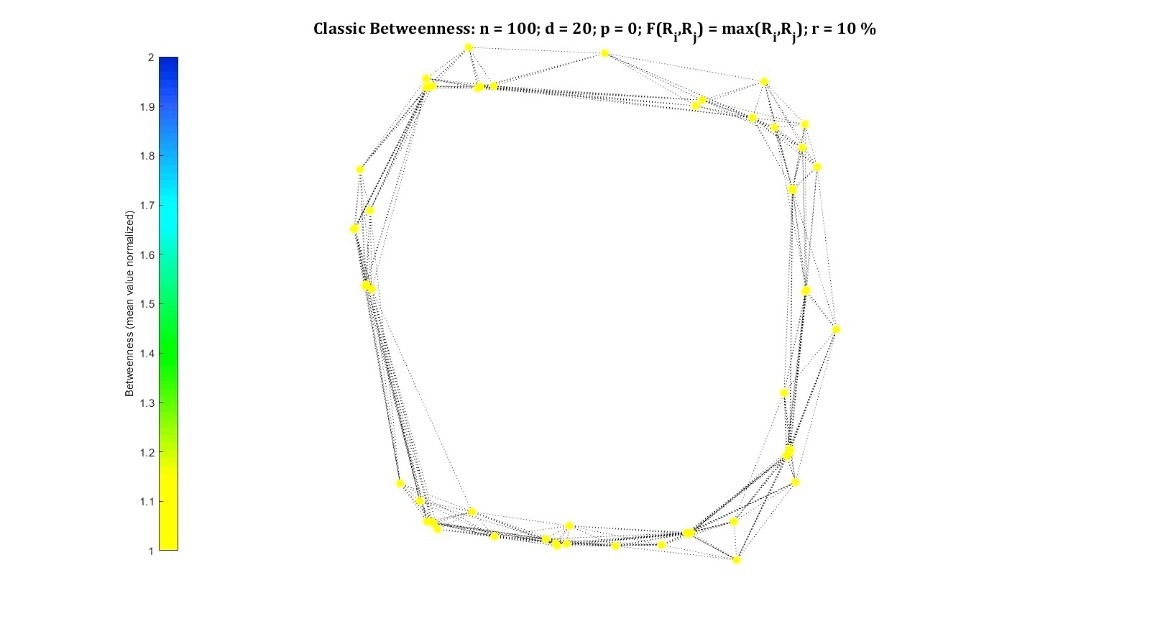

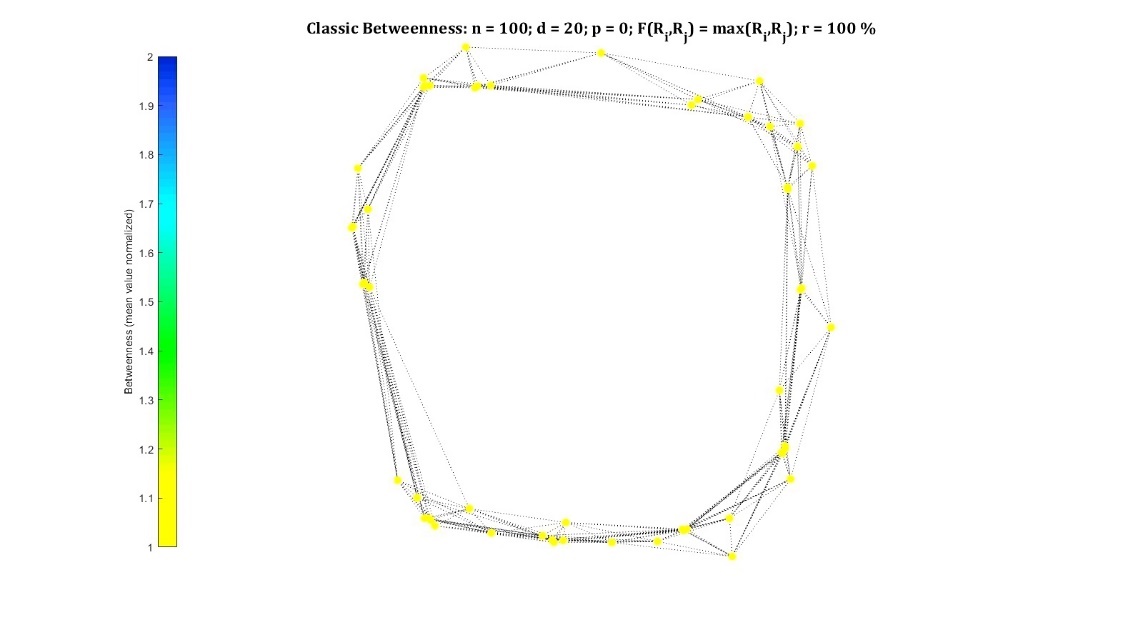


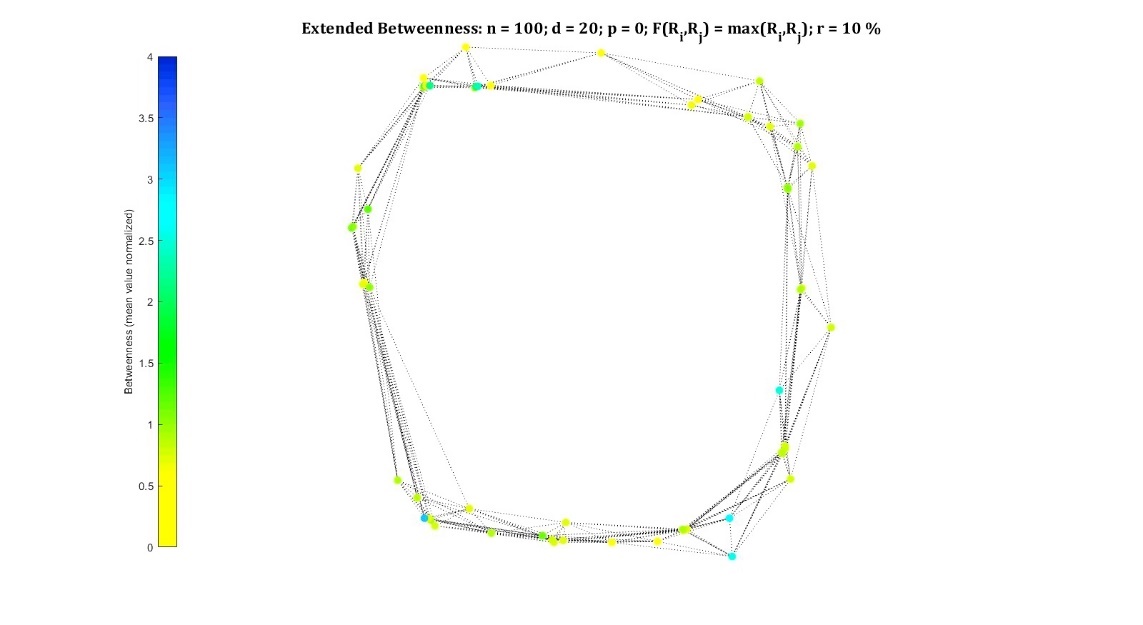

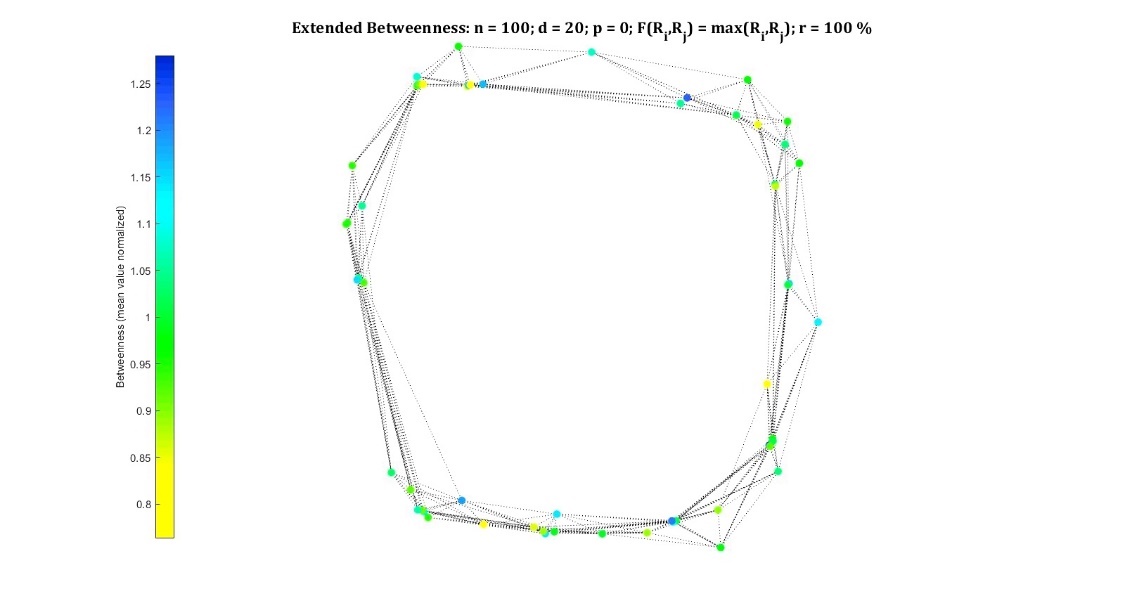


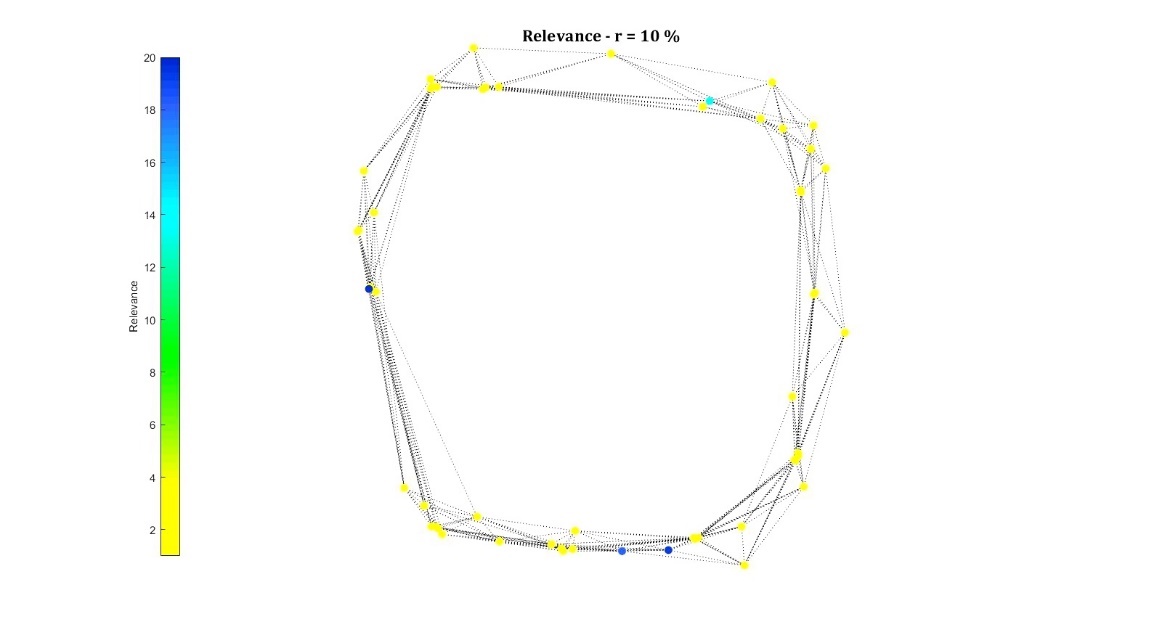

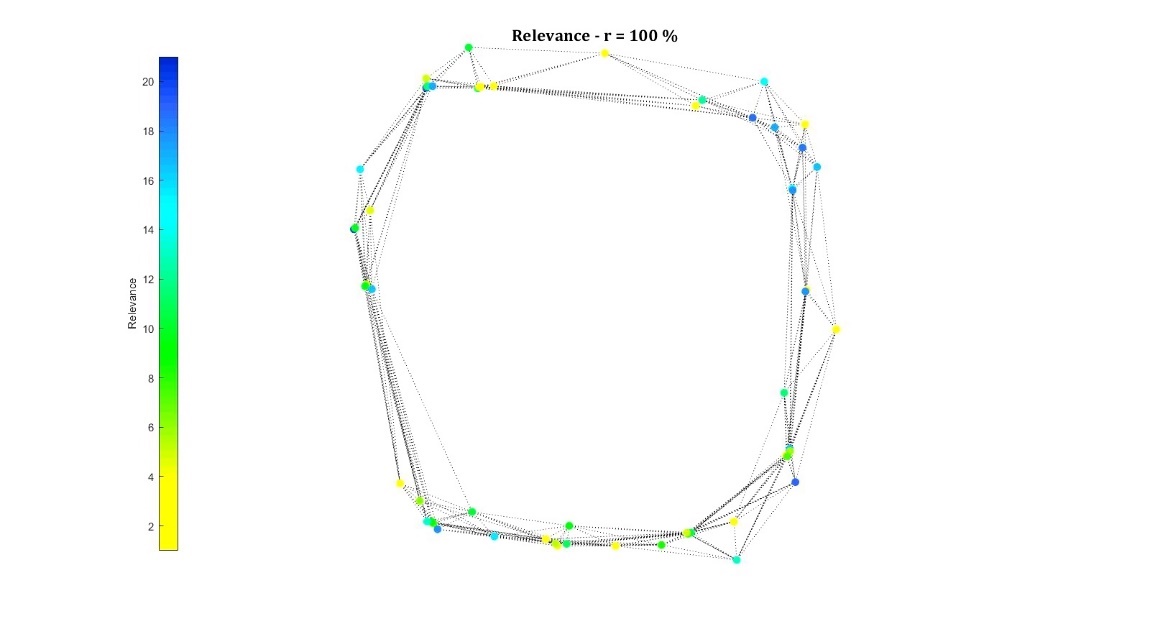


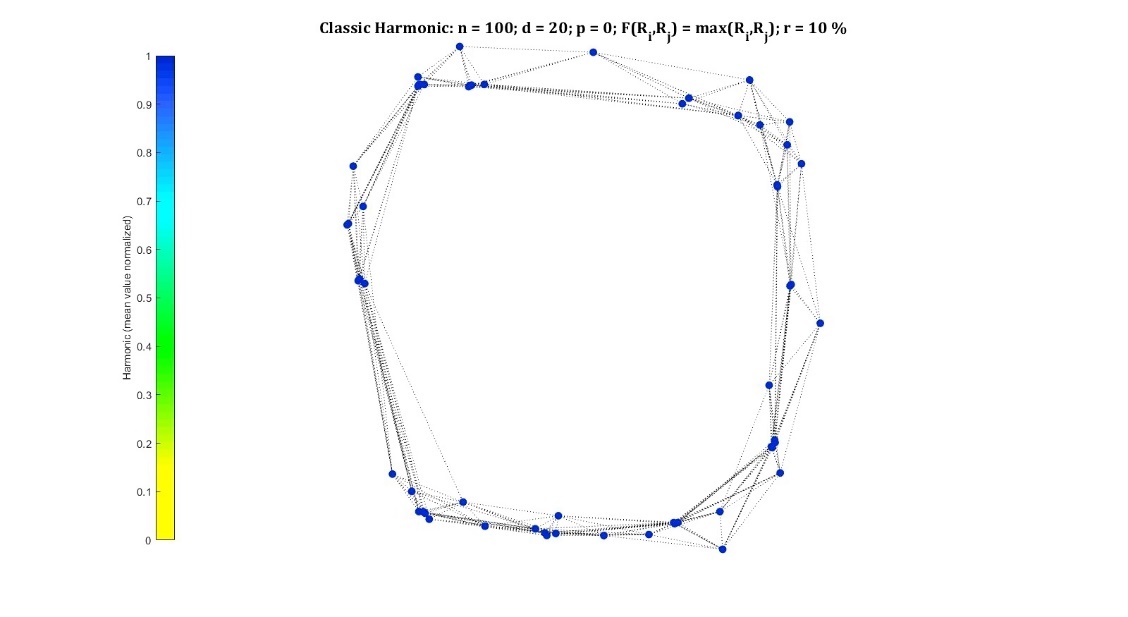

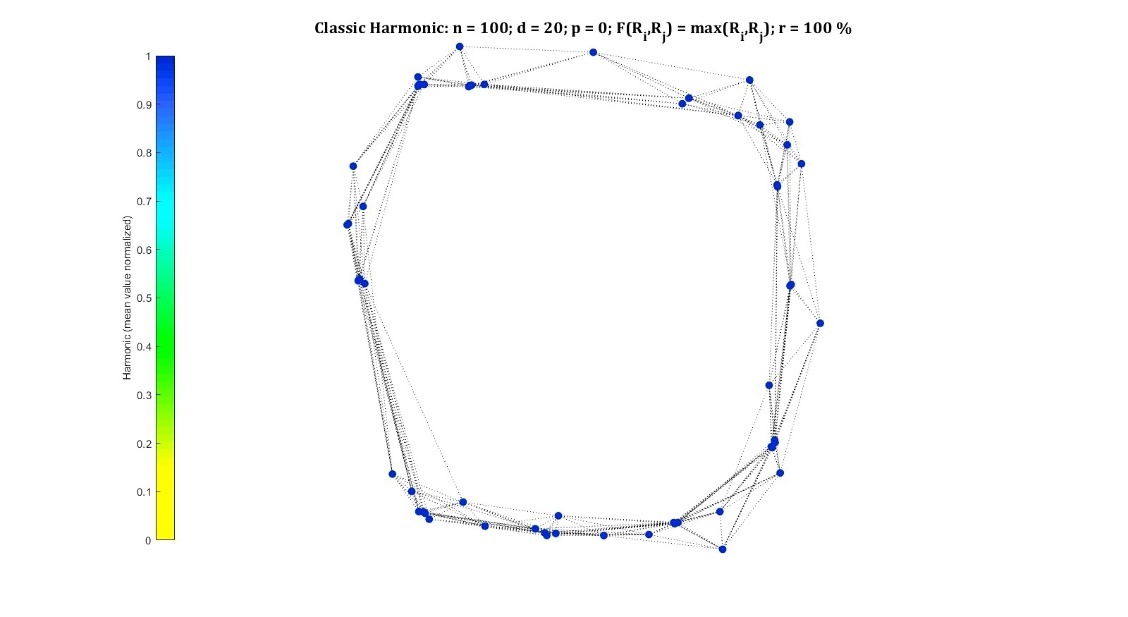


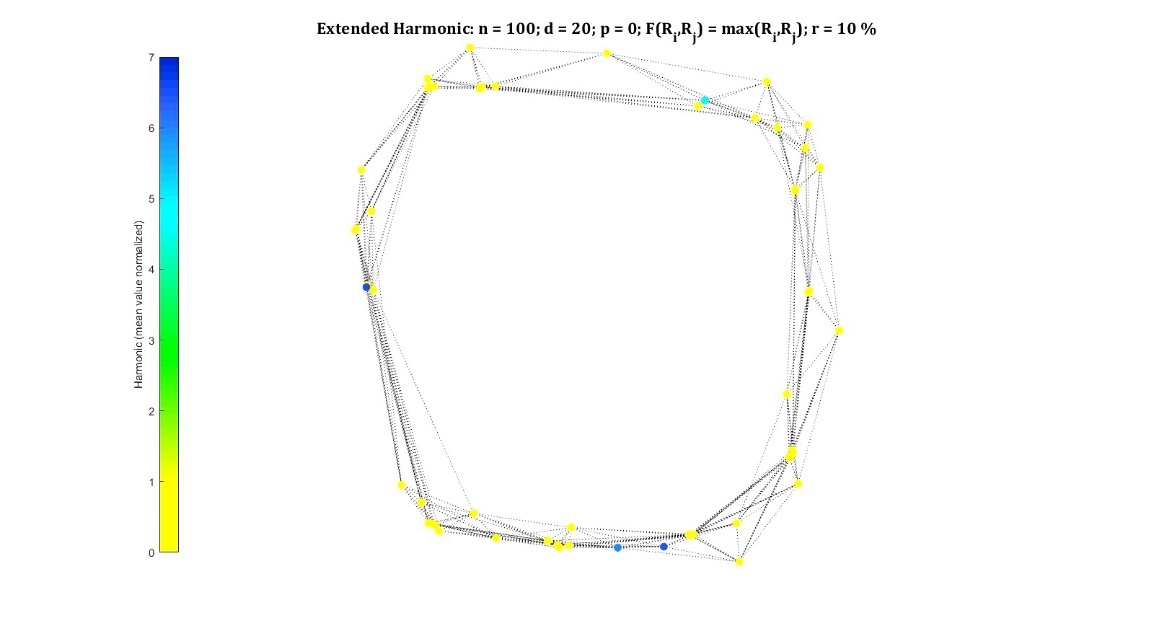

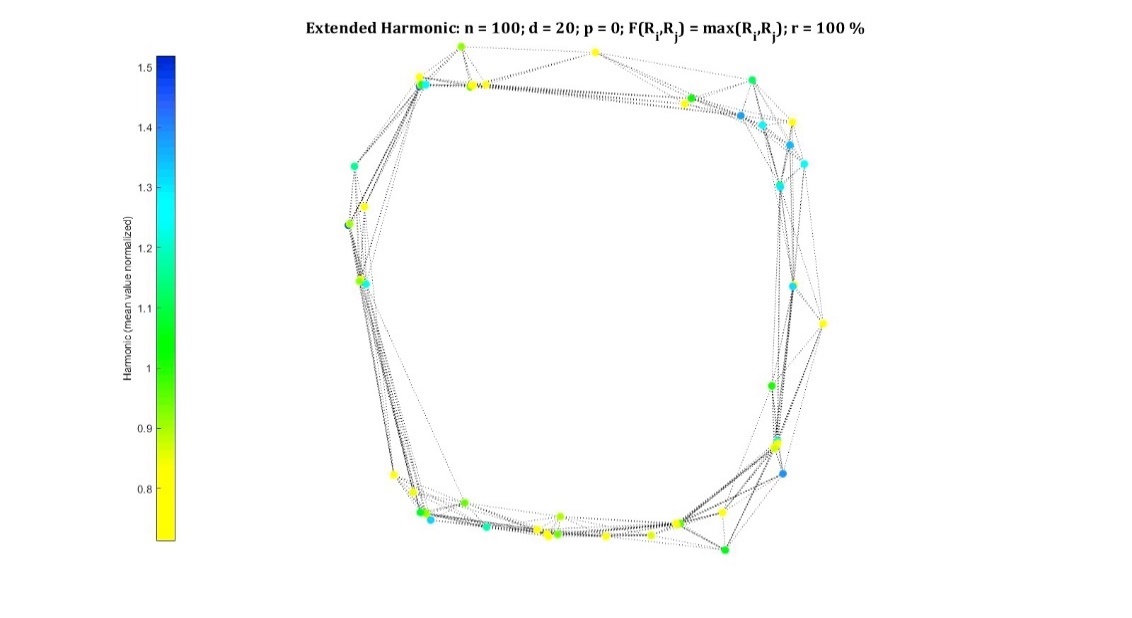


Figure 3B: regular network with 100 vertices corresponding to *r*=10% (left-panel) and *r*=100% (right-panel) of the randomly assigned intrinsic relevance. The coloured vertices refer to the colour-bar indicating the values of the: standard betweenness (1^st^ panel), relevance-based betweenness (2^nd^ panel), intrinsic relevance of vertices (3^rd^ panel), standard harmonic centrality (4^th^ panel) and relevance-based harmonic centrality (5^th^ panel). The relevance-based metrics refers to the function *f*(*R_i_*, *R_j_*) = max(*R_i_*, *R_j_*) as in Figure 3A.


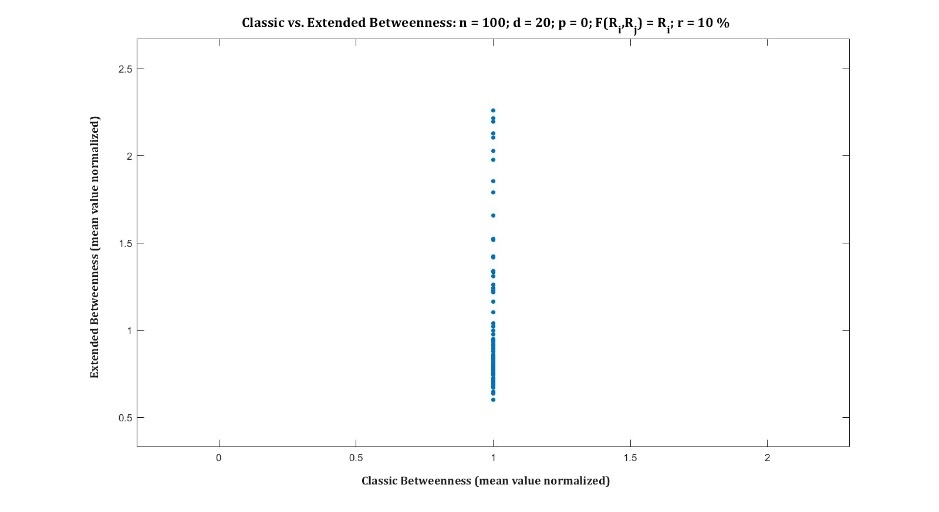

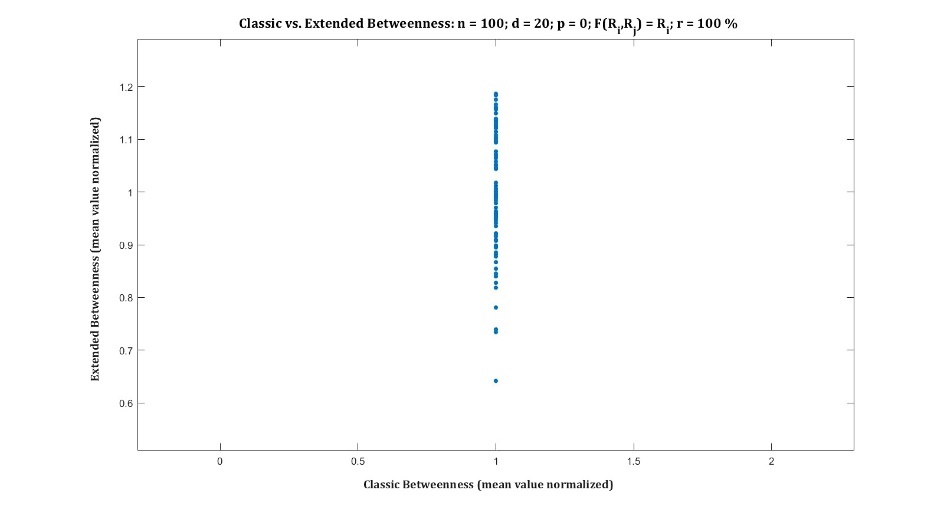


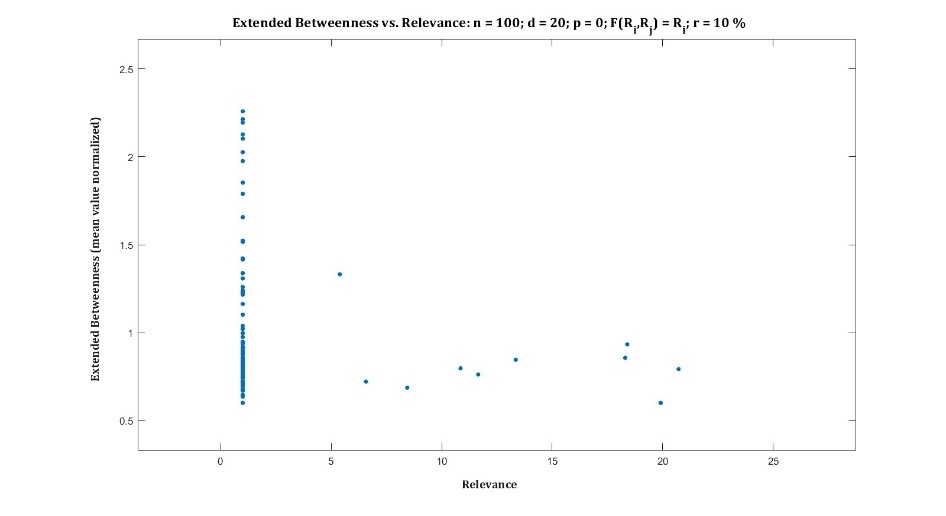

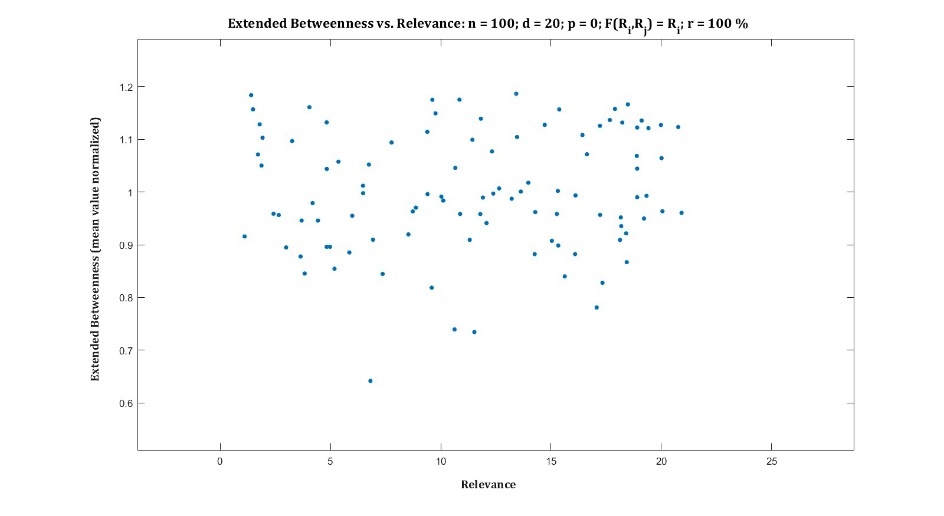


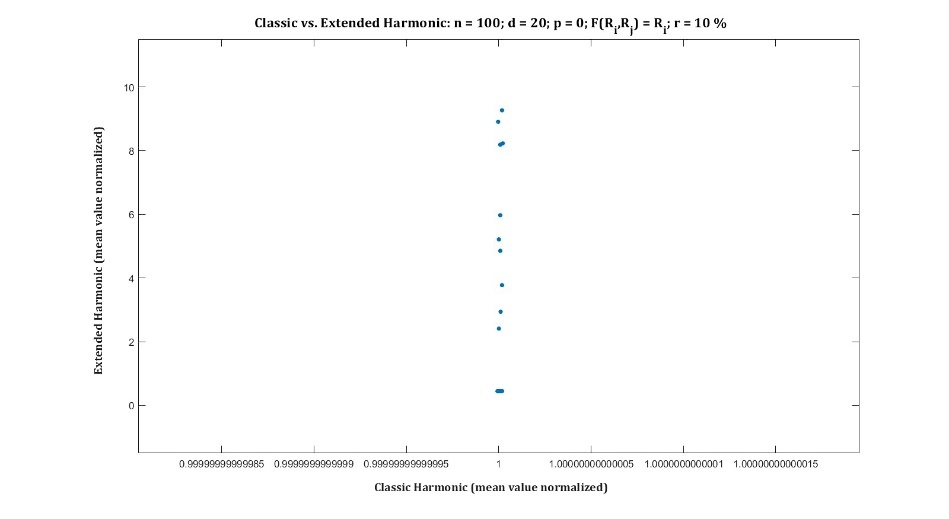

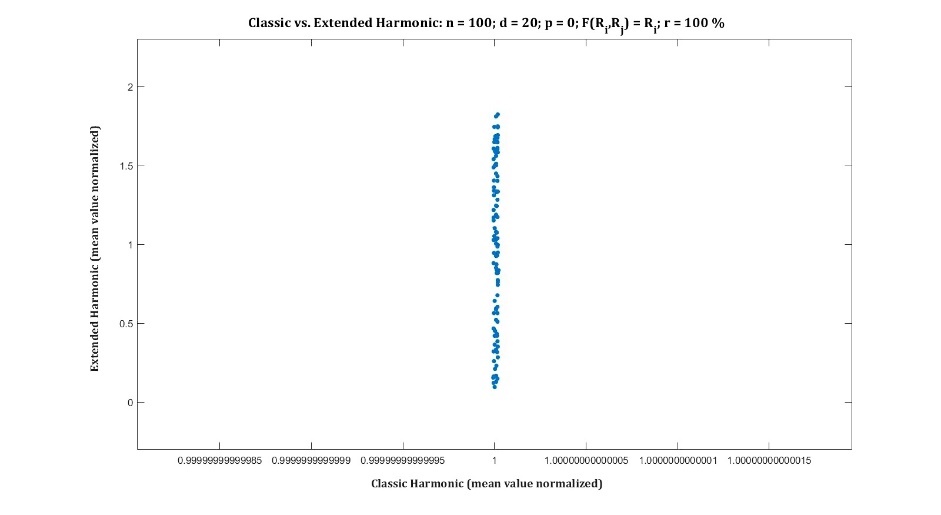


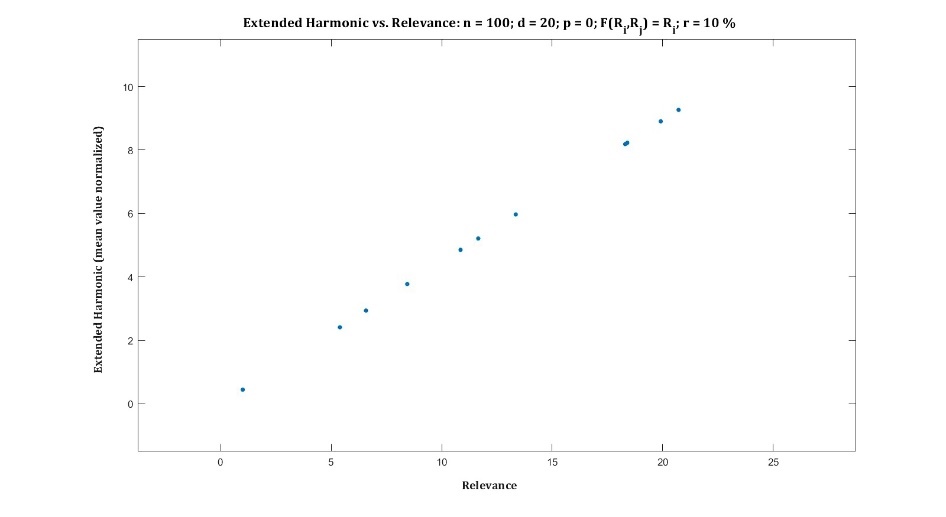

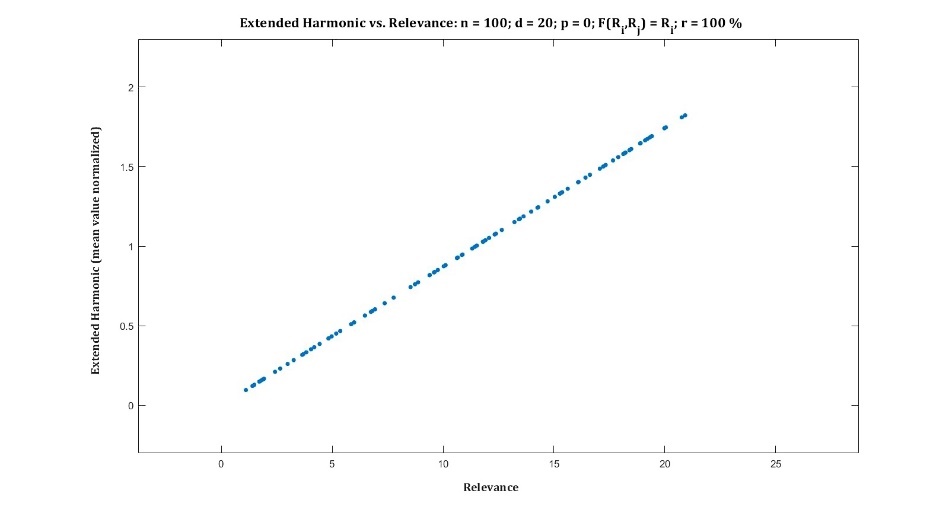


Figure 4A: *f*(*R_i_*, *R_j_*) = *R_i_* performed for a regular network with 100 vertices. It reports: standard *vs* relevance-based betweenness with *r*=10% (1^st^ panel-left) and *r*=100% (1^st^ panel-right), intrinsic relevance *vs* relevance-based betweenness with *r* =10% (2^nd^ panel-left) and *r* =100% (2^nd^ panel-right), classic *vs* relevance-based harmonic centrality with *r* =10% (3^rd^ panel-left) and *r* =100% (3^rd^ panel-right), and intrinsic relevance *vs* relevance-based harmonic with *r* =10% (4^th^ panel-left) and *r* =100% (4^th^ panel-right).


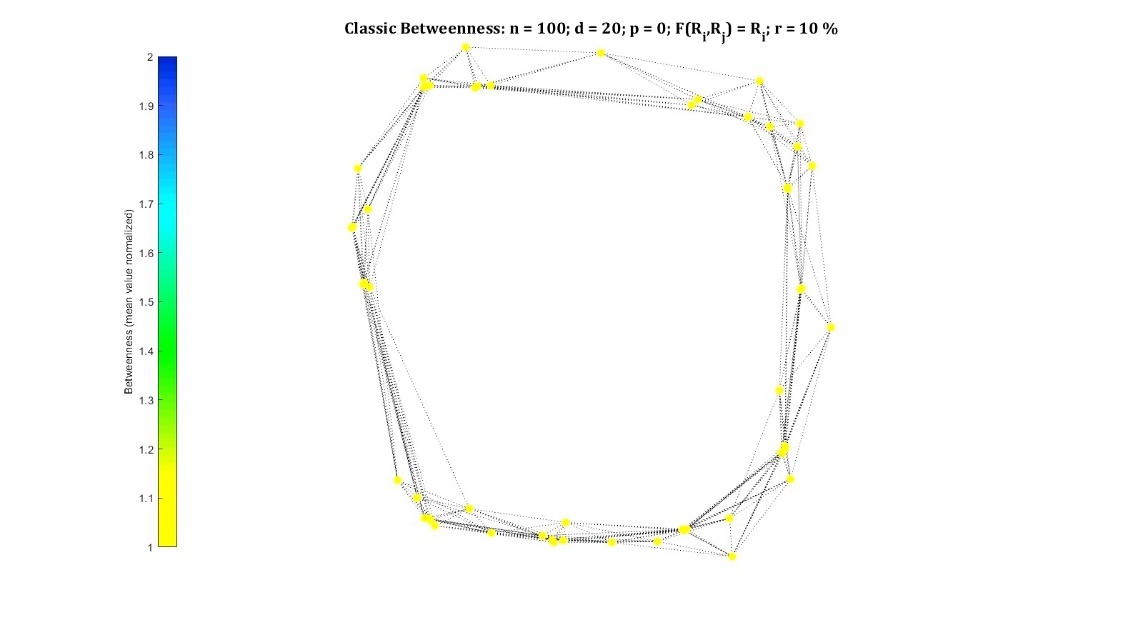

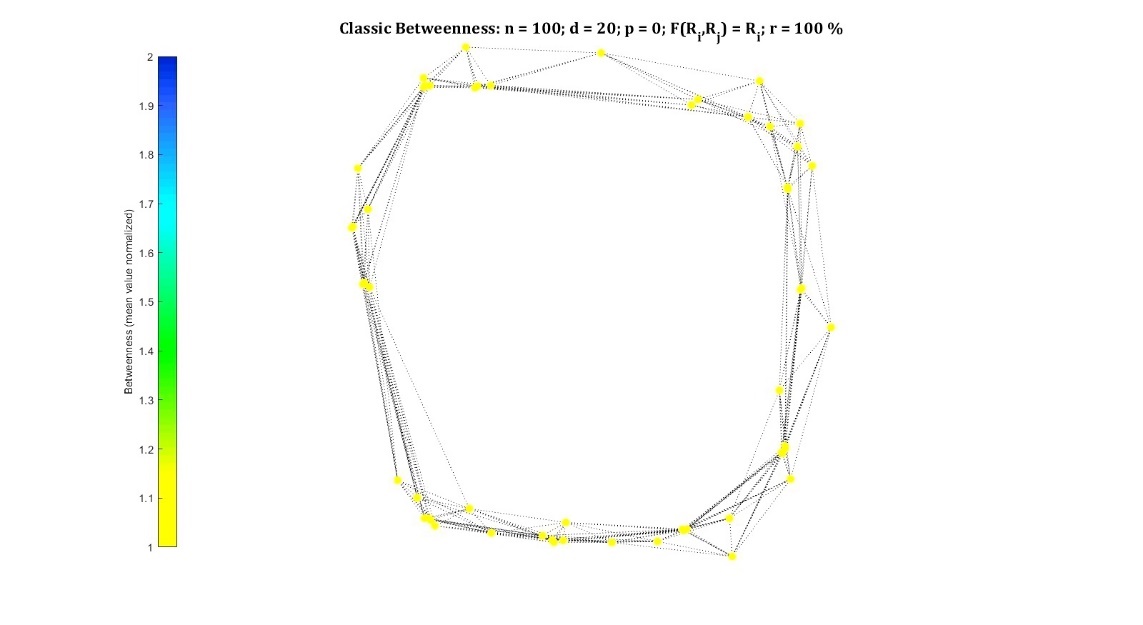


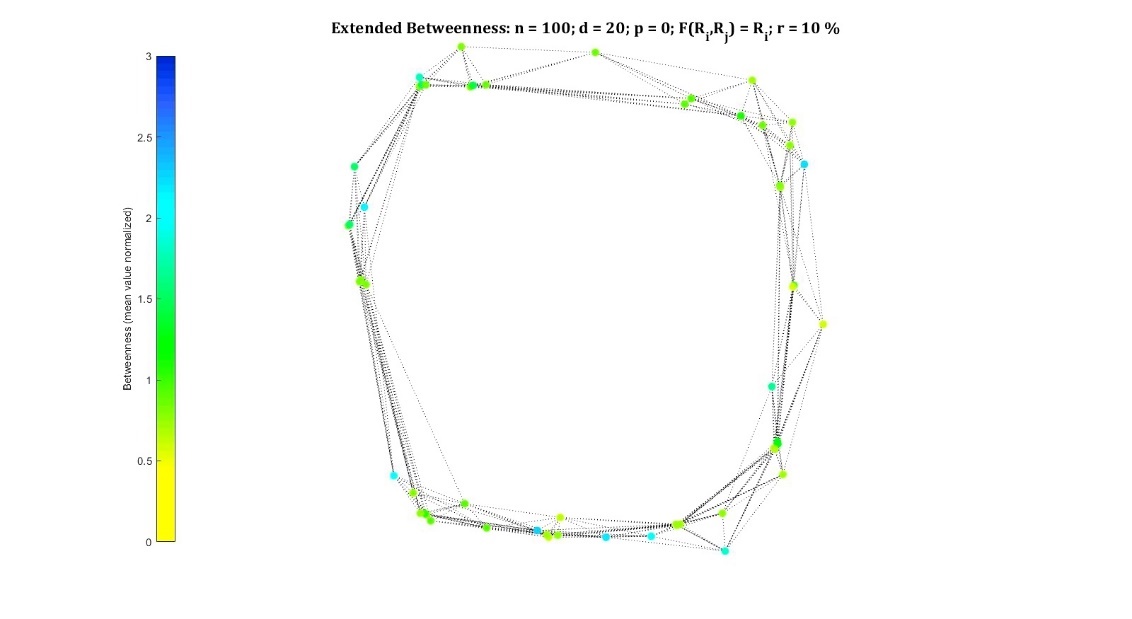

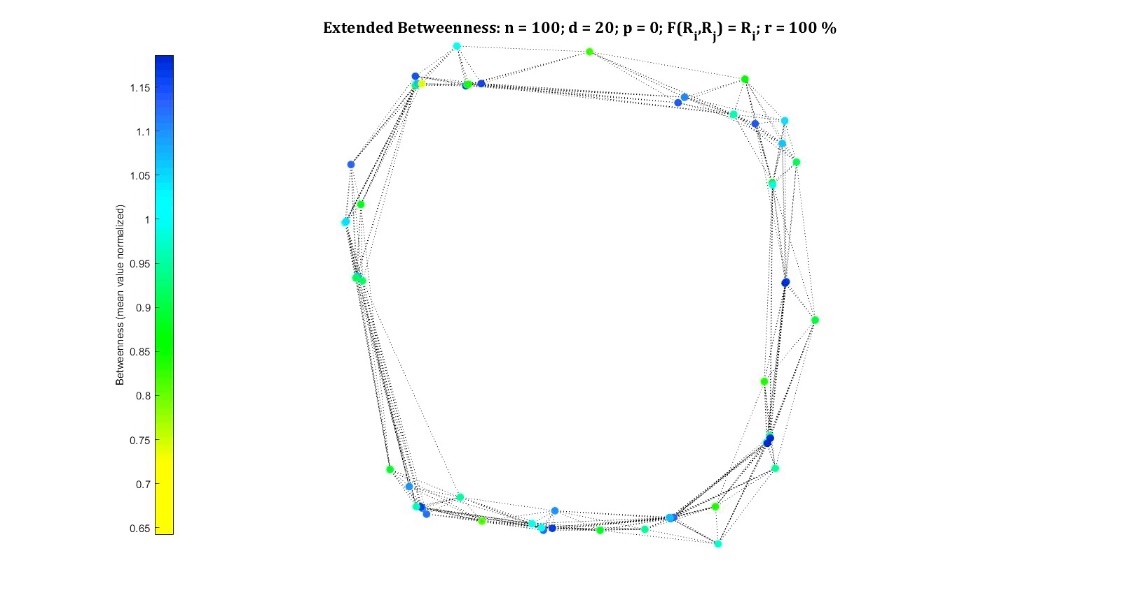


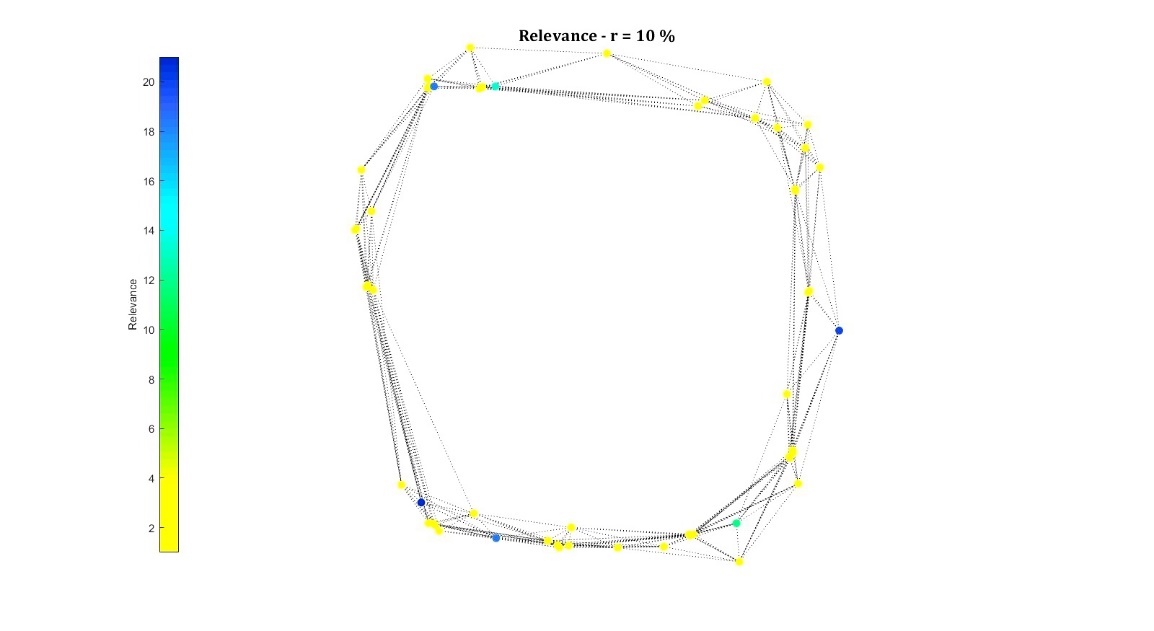

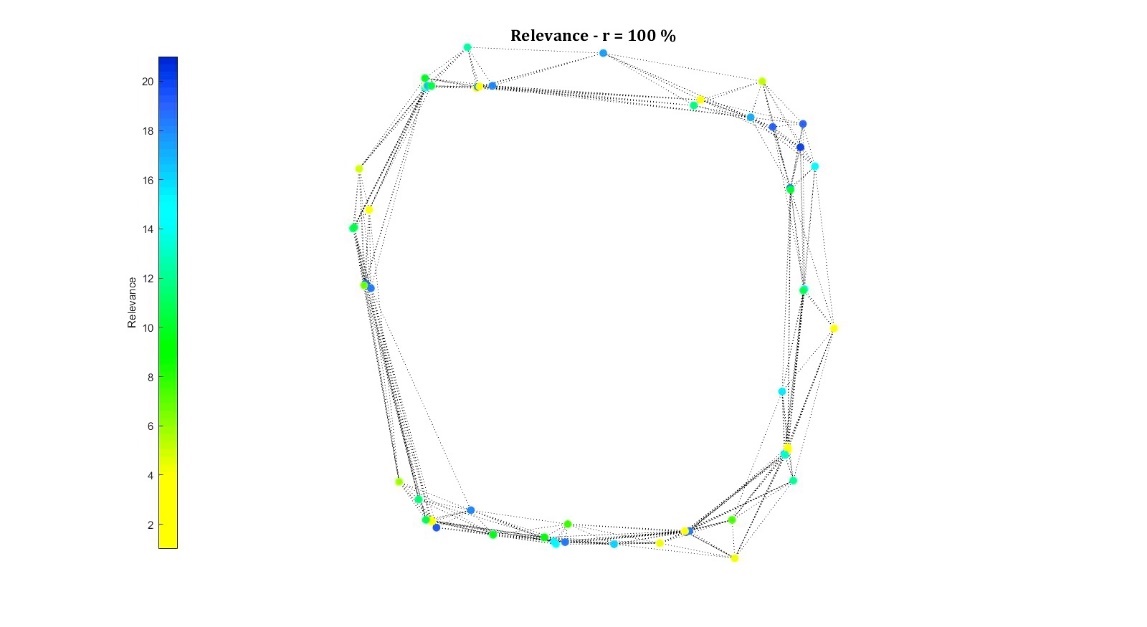


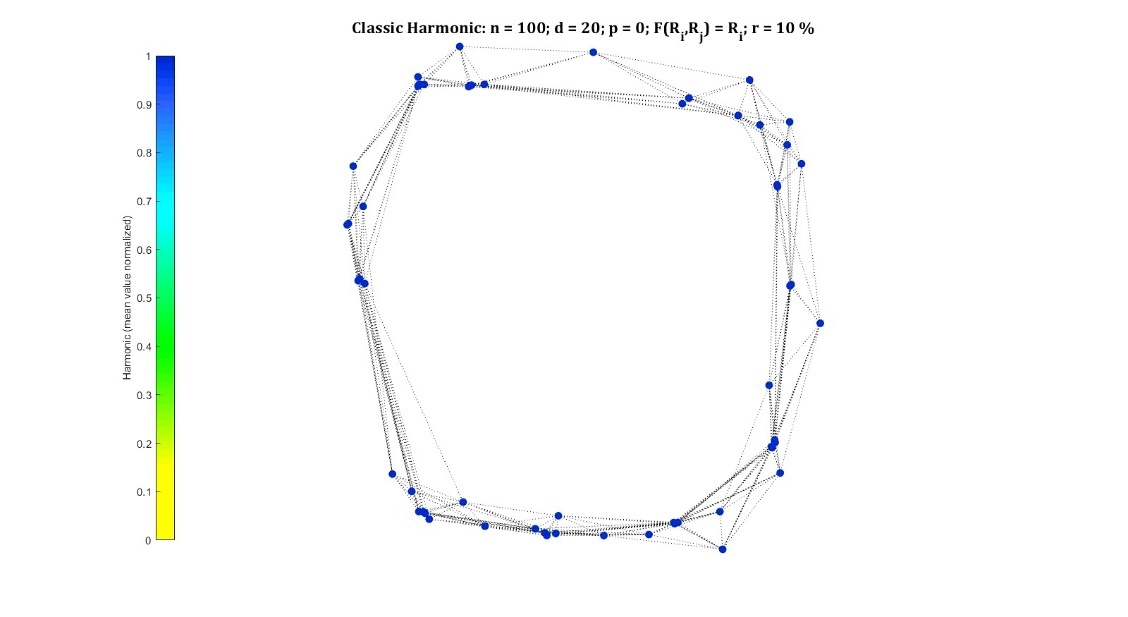

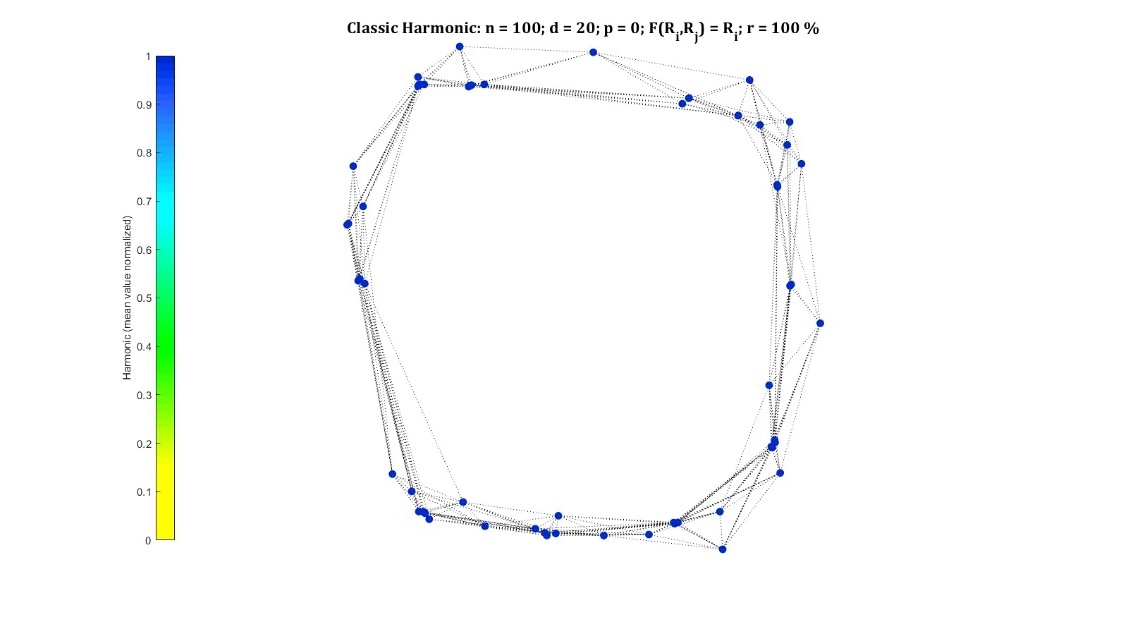


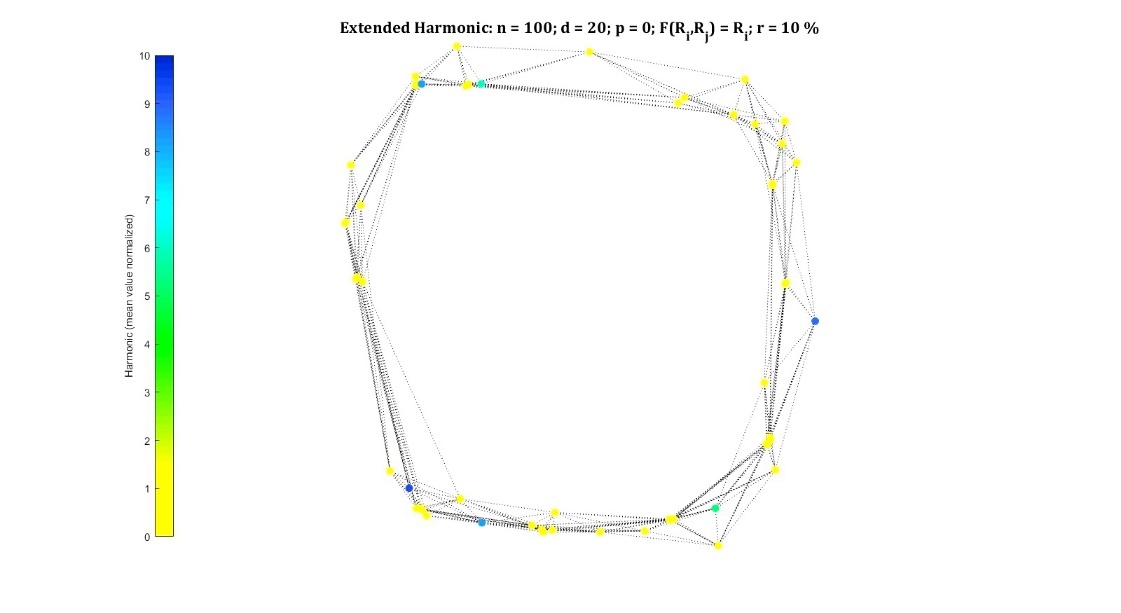

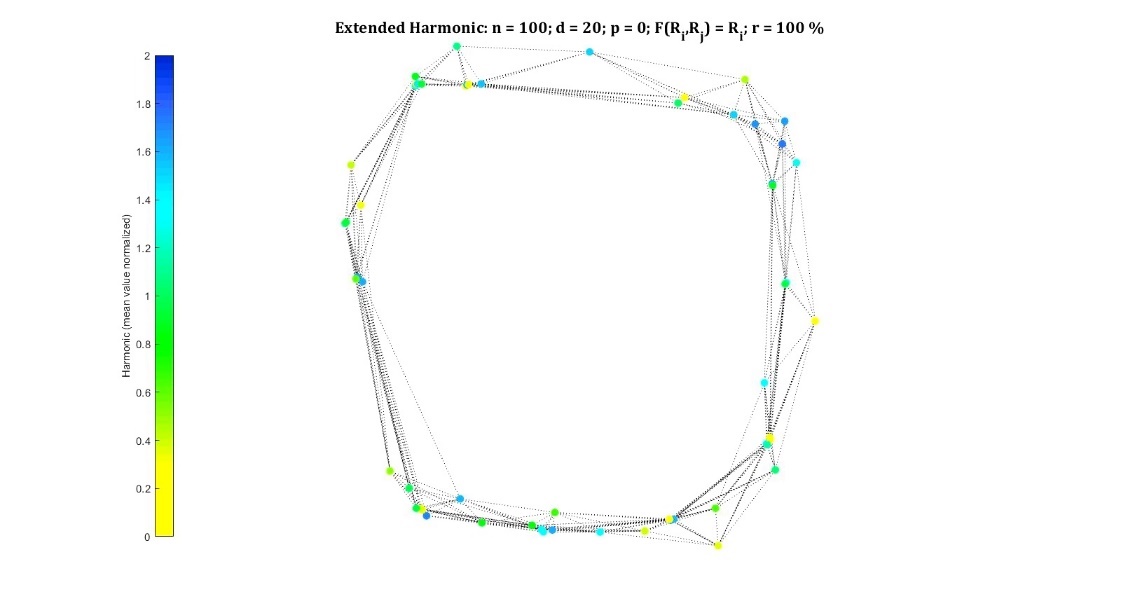


Figure 4B: regular network with 100 vertices corresponding to *r*=10% (left-panel) and *r*=100% (right-panel) of the randomly assigned intrinsic relevance. The coloured vertices refer to the colour-bar indicating the values of the: standard betweenness (1^st^ panel), relevance-based betweenness (2^nd^ panel), intrinsic relevance of vertices (3^rd^ panel), standard harmonic centrality (4^th^ panel) and relevance-based harmonic centrality (5^th^ panel). The relevance-based metrics refers to the function *f*(*R_i_*, *R_j_*) = *R_i_* as in Figure 4A.


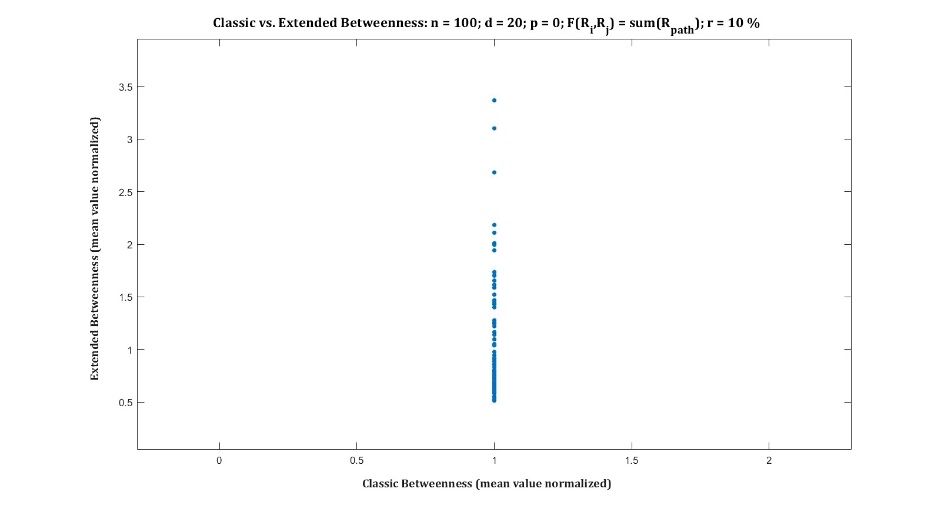

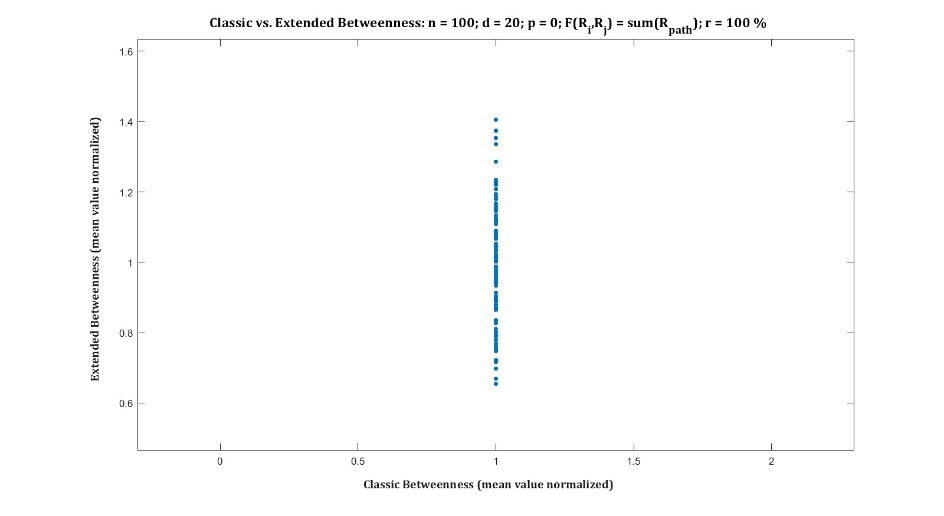


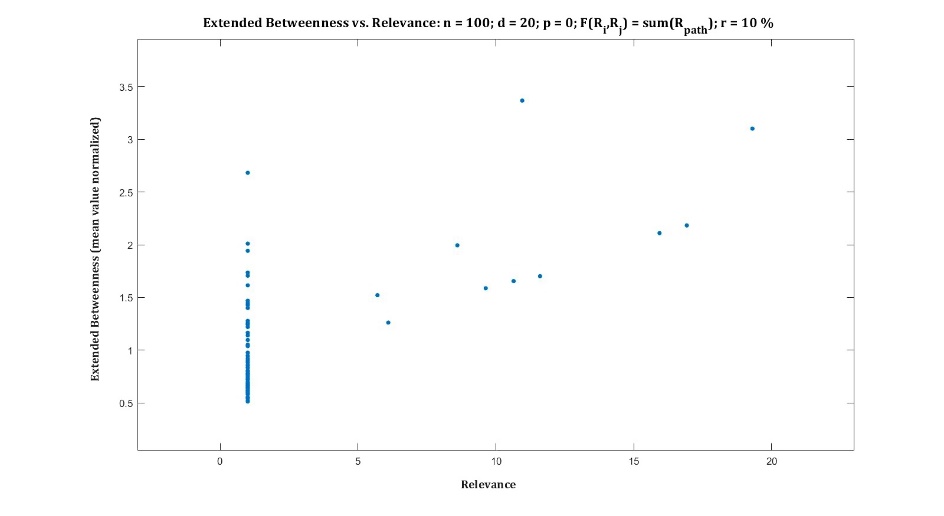

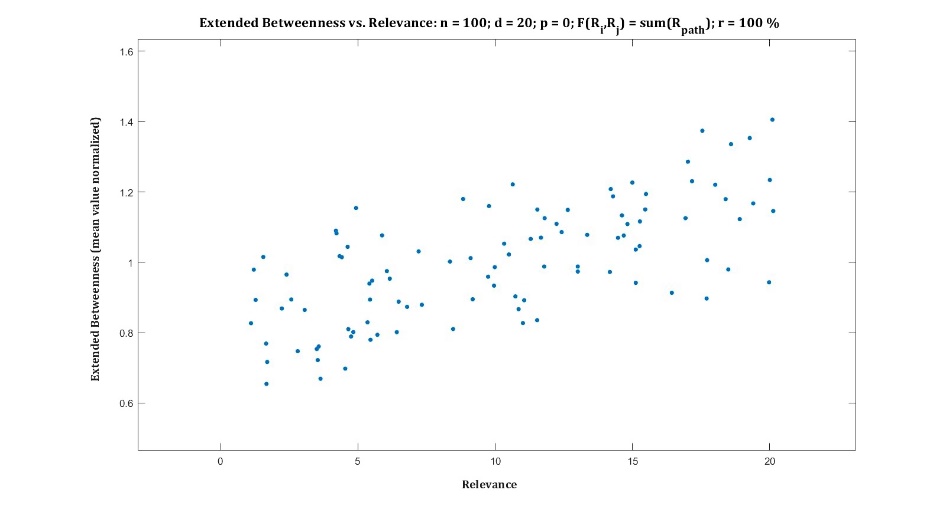


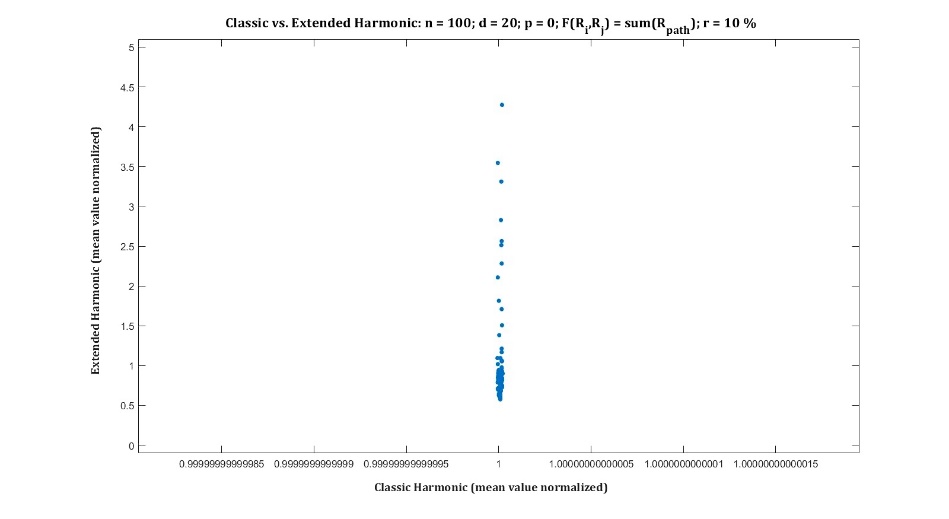

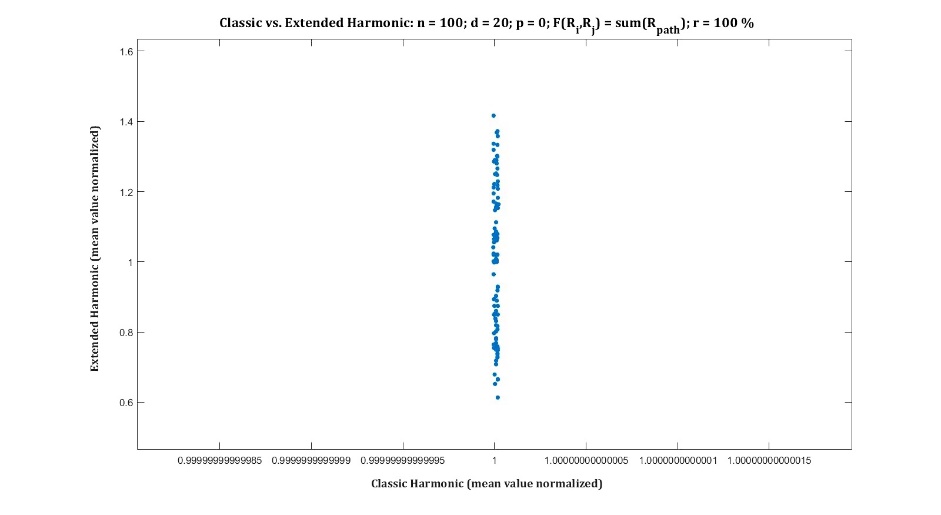


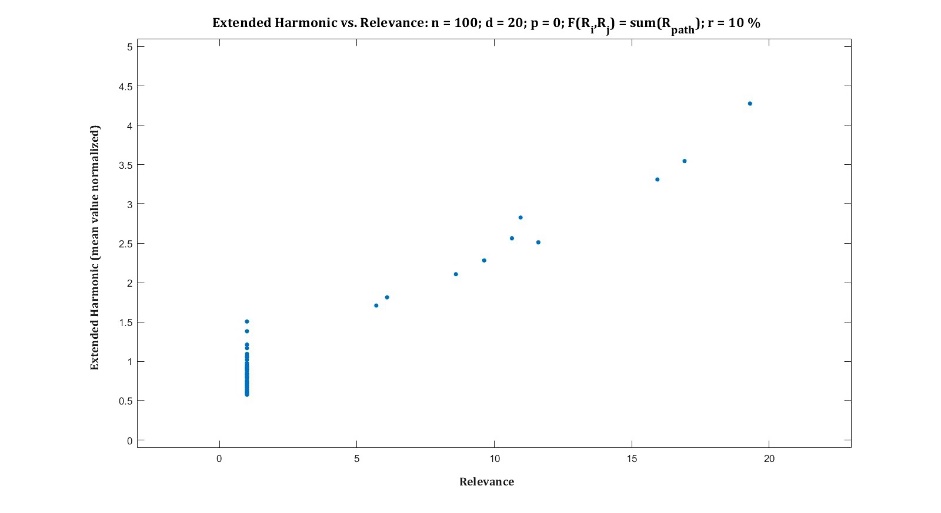

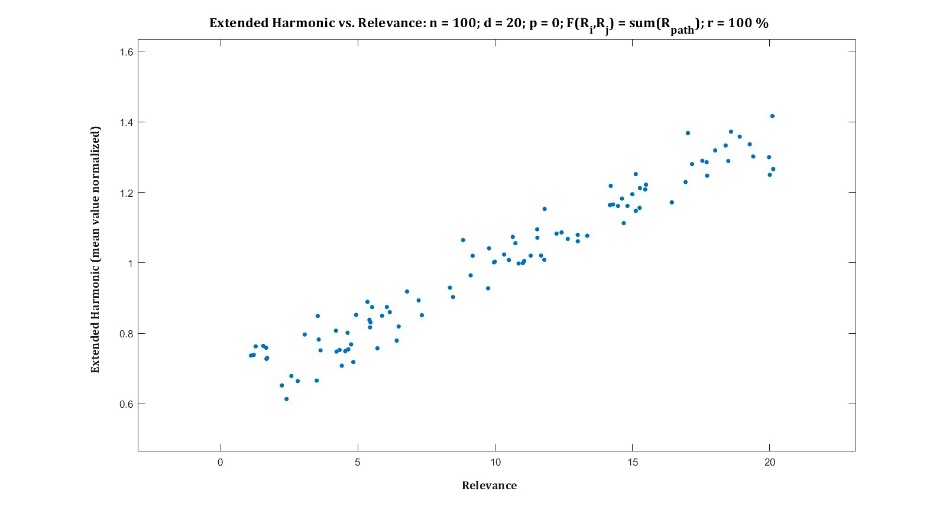


Figure 5A: *f*(*R_i_*, *R_j_*) = Σ*R_path_* performed for a regular network with 100 vertices. It reports: standard *vs* relevance-based betweenness with *r*=10% (1^st^ panel-left) and *r*=100% (1^st^ panel-right), intrinsic relevance *vs* relevance-based betweenness with *r* =10% (2^nd^ panel-left) and *r* =100% (2^nd^ panel-right), classic *vs* relevance-based harmonic centrality with *r* =10% (3^rd^ panel-left) and *r* =100% (3^rd^ panel-right), and intrinsic relevance *vs* relevance-based harmonic with *r* =10% (4^th^ panel-left) and *r* =100% (4^th^ panel-right).


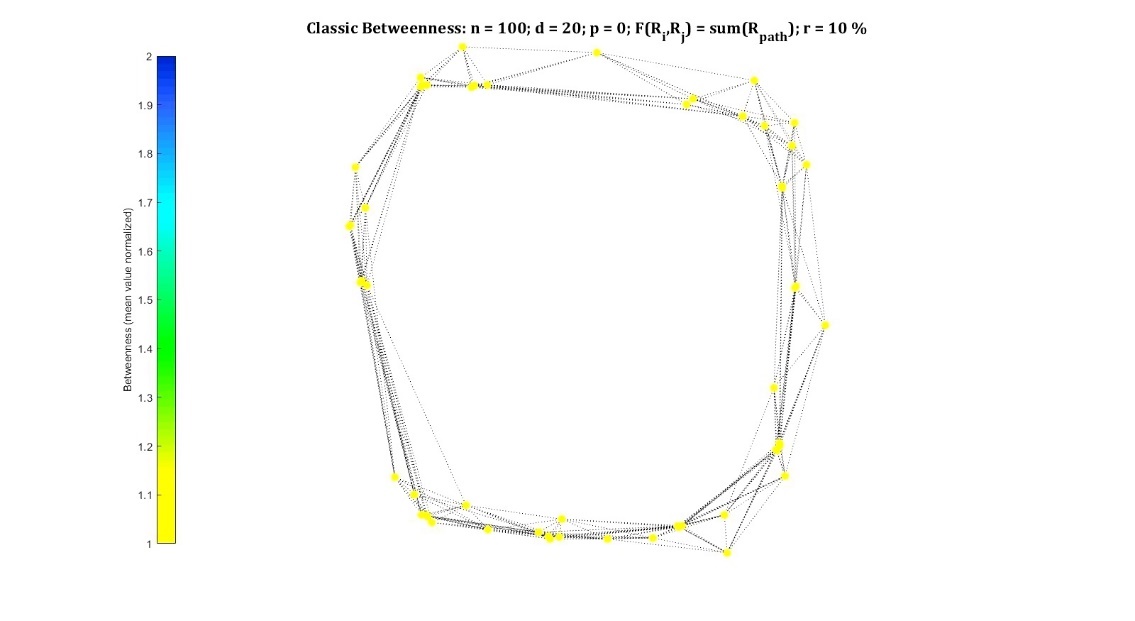

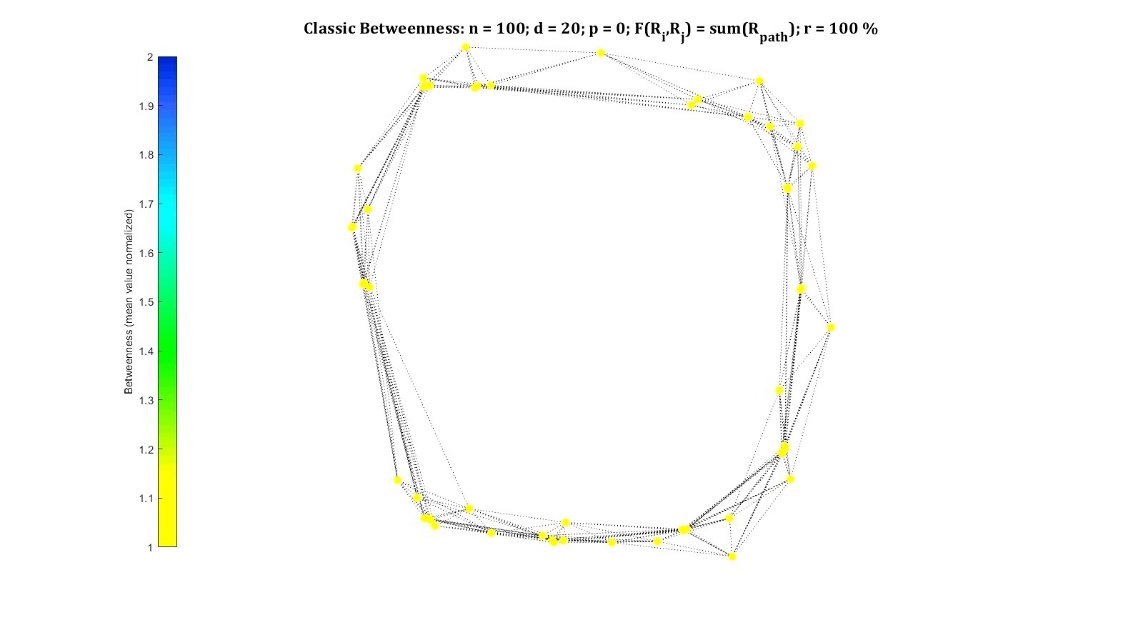


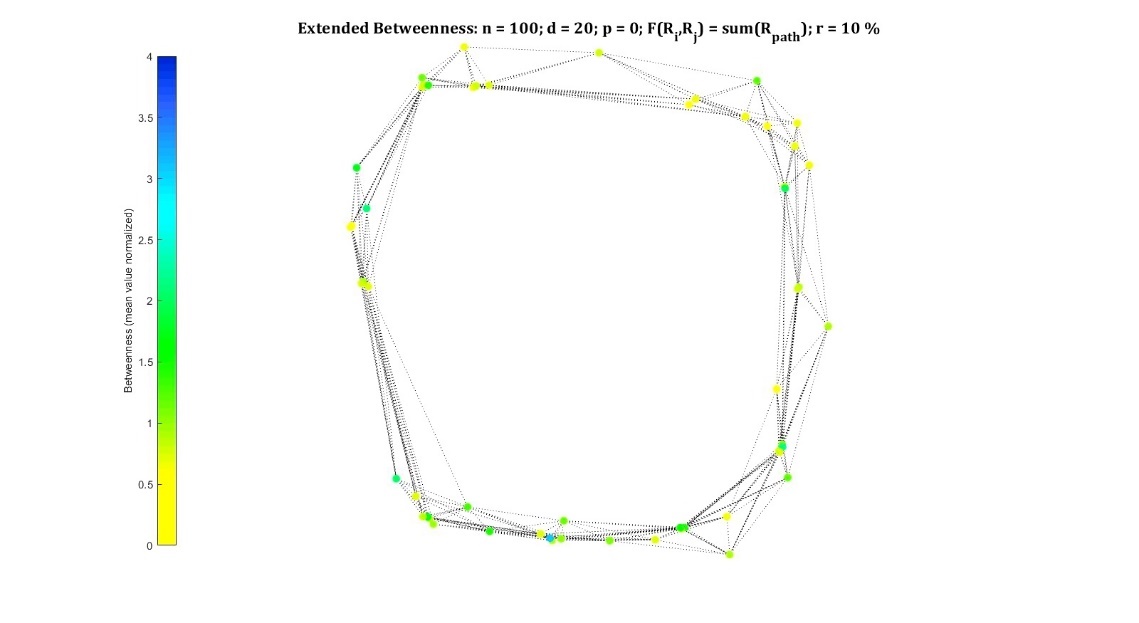

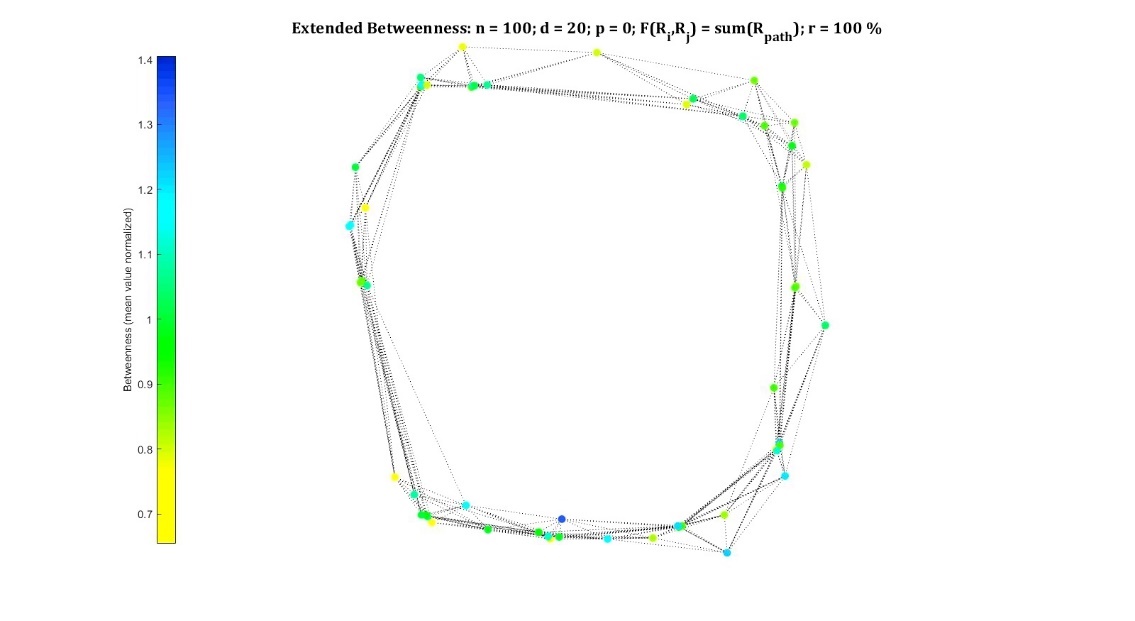


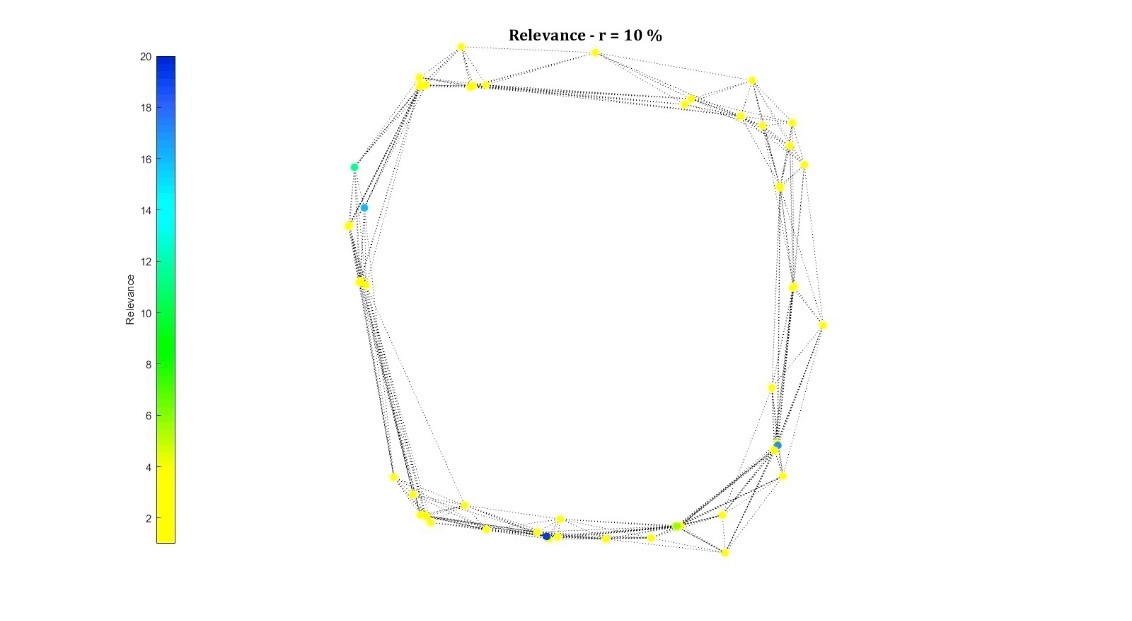

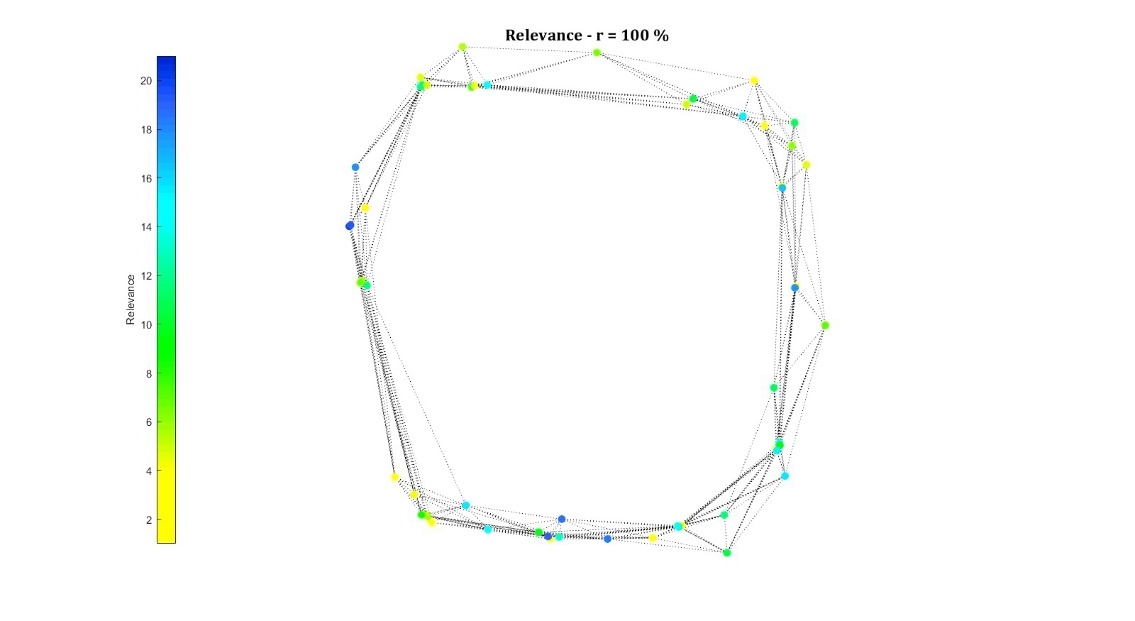


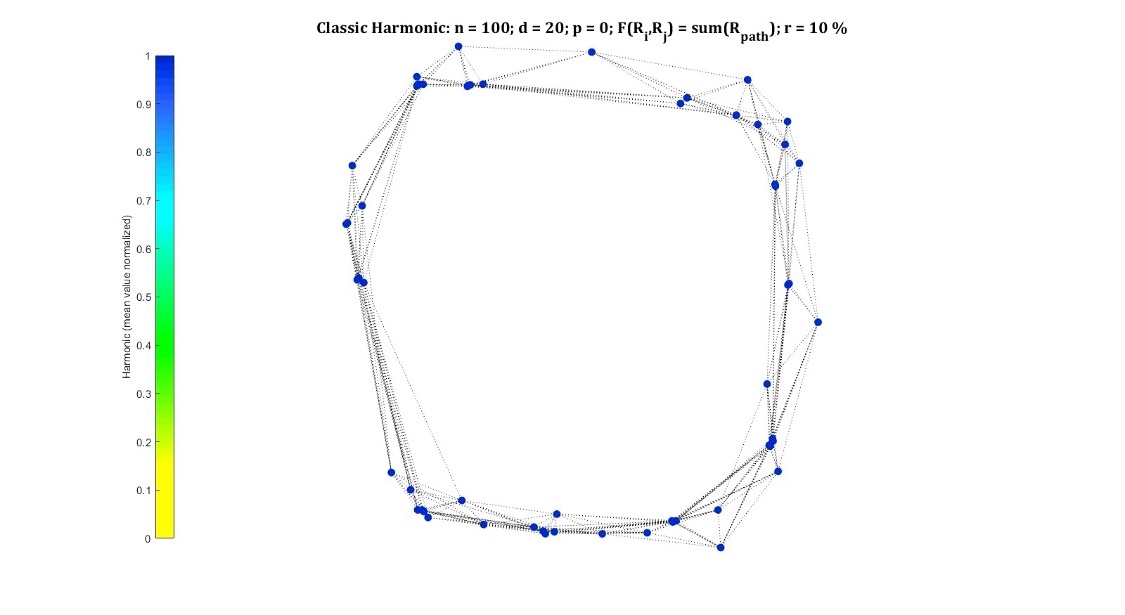

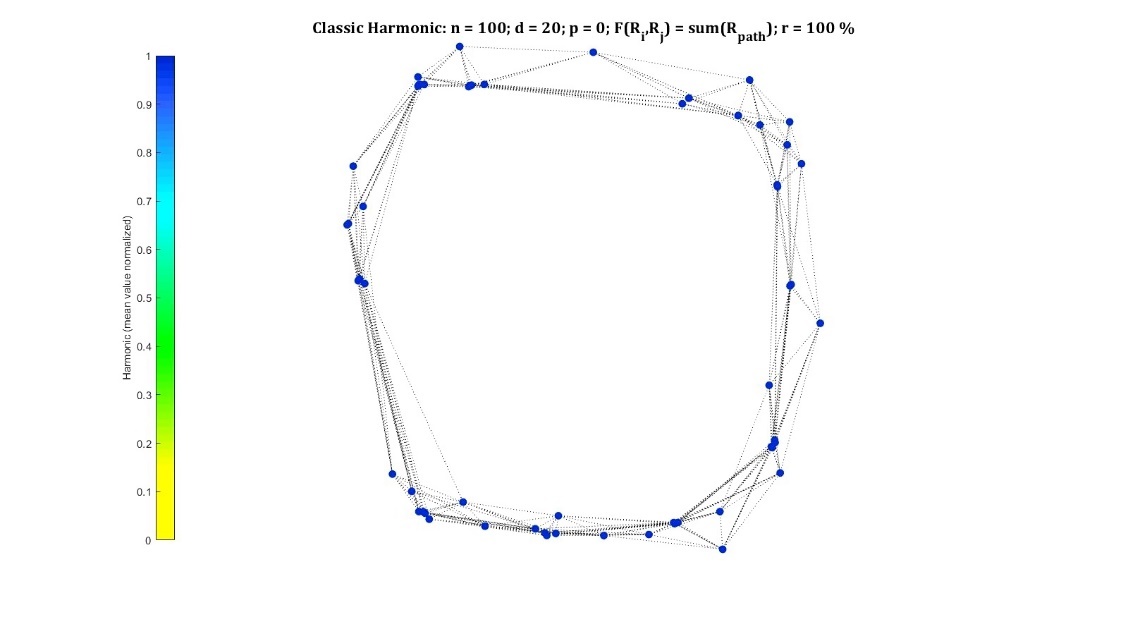


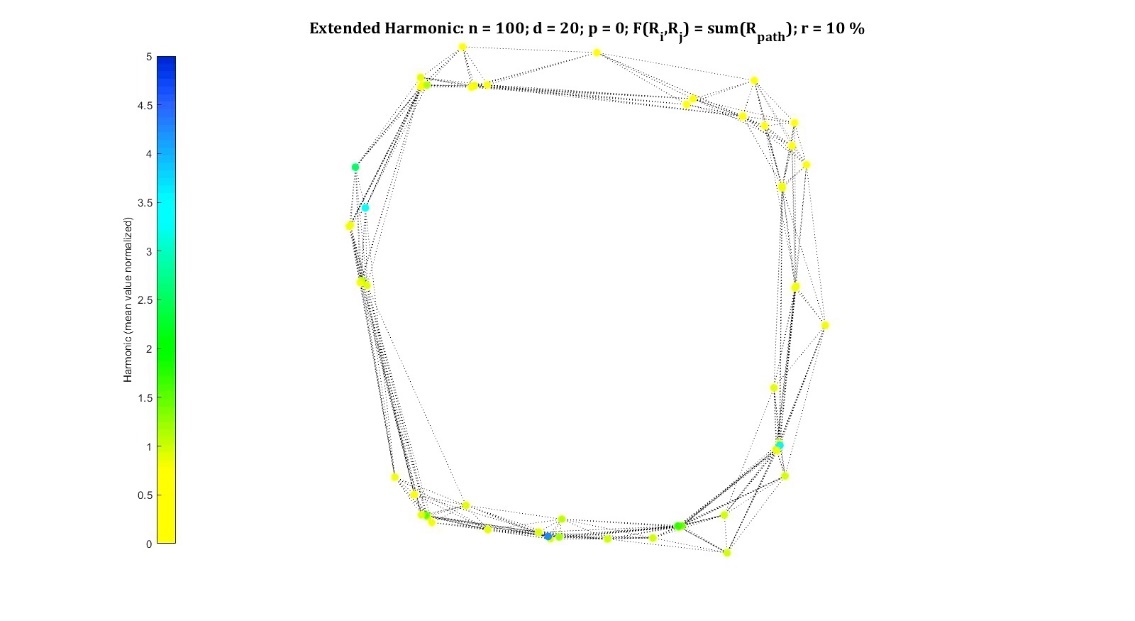

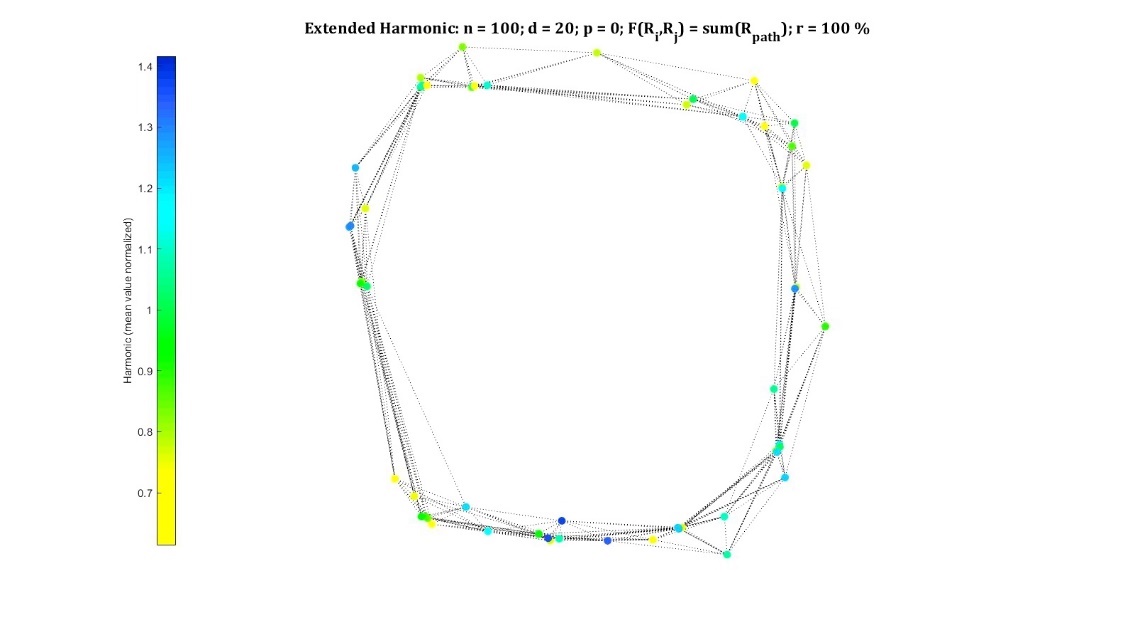


Figure 5B: regular network with 100 vertices corresponding to *r*=10% (left-panel) and *r*=100% (right-panel) of the randomly assigned intrinsic relevance. The coloured vertices refer to the colour-bar indicating the values of the: standard betweenness (1^st^ panel), relevance-based betweenness (2^nd^ panel), intrinsic relevance of vertices (3^rd^ panel), standard harmonic centrality (4^th^ panel) and relevance-based harmonic centrality (5^th^ panel). The relevance-based metrics refers to the function *f*(*R_i_*, *R_j_*) = Σ*R_path_* as in Figure 5A.


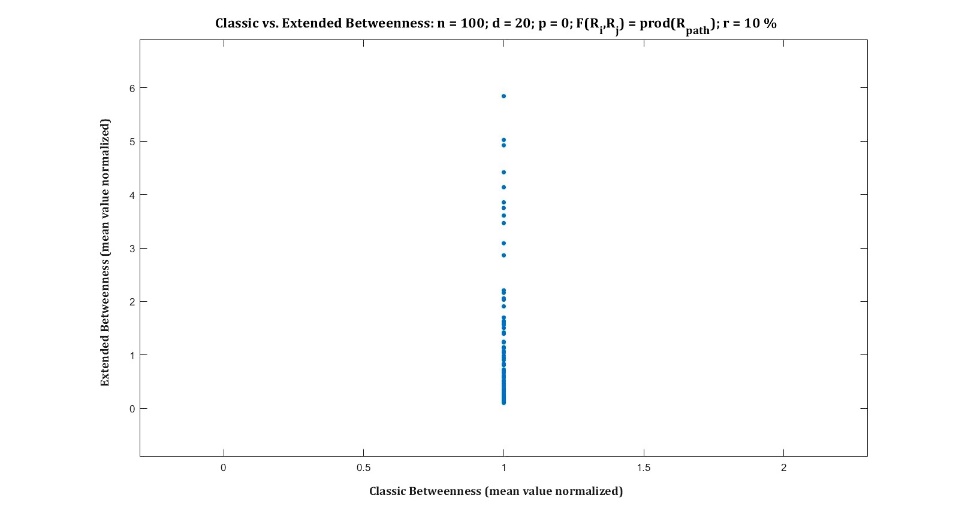

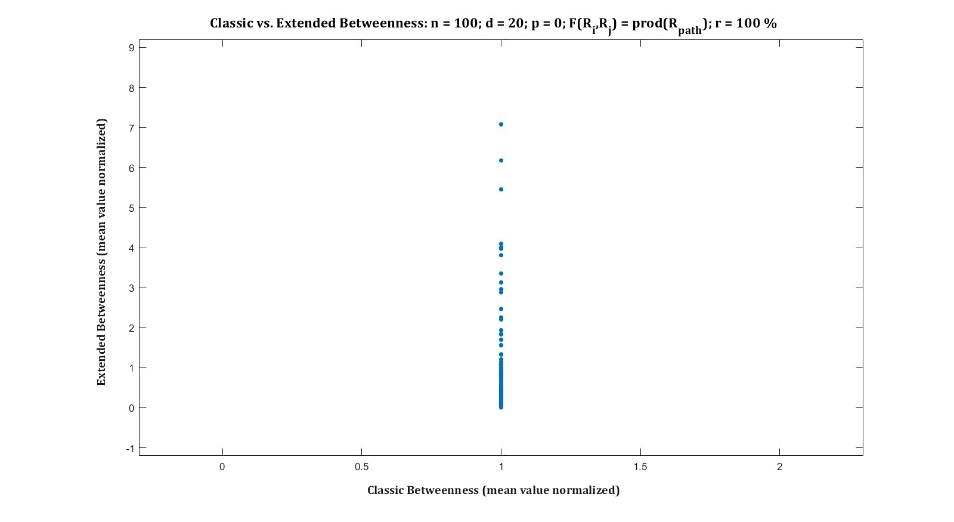


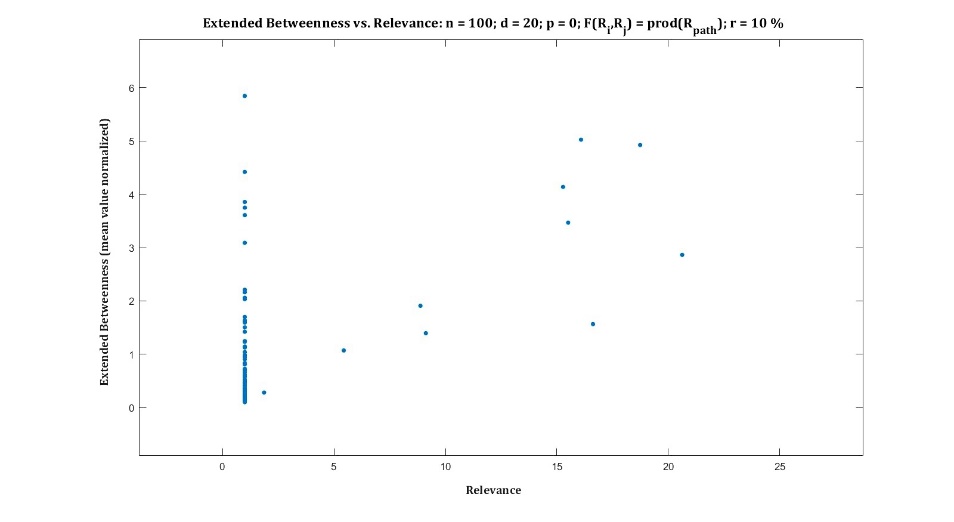

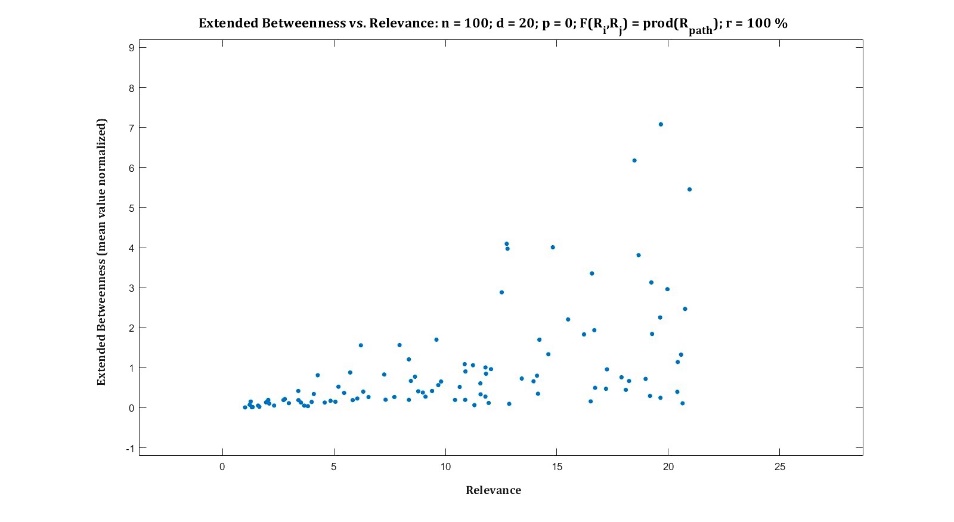


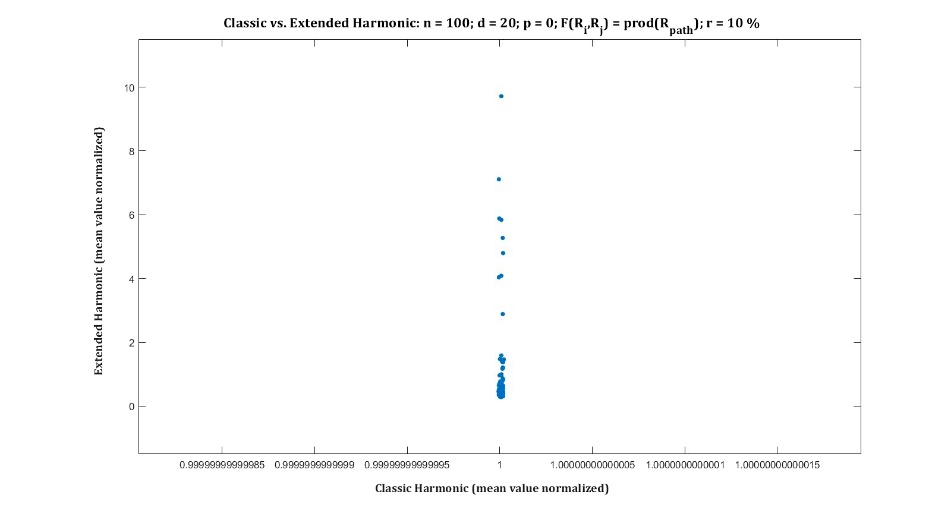

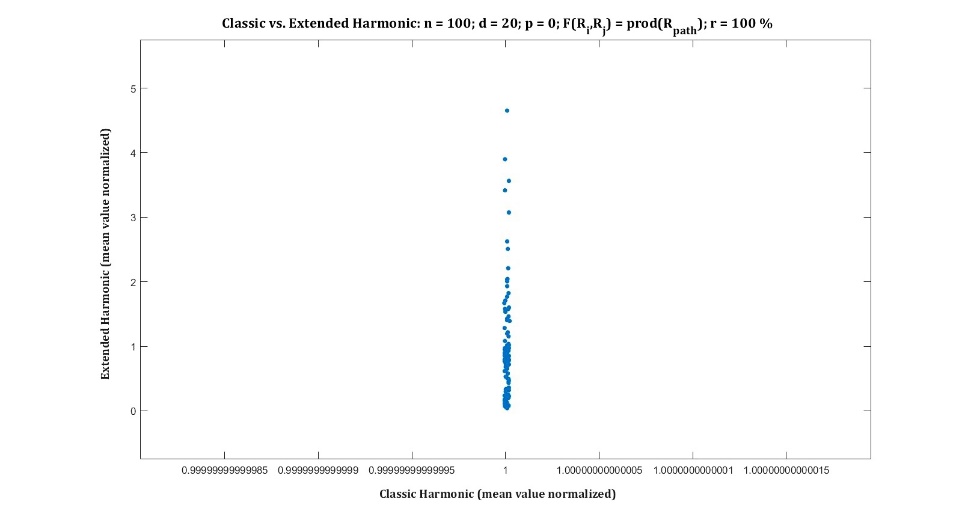


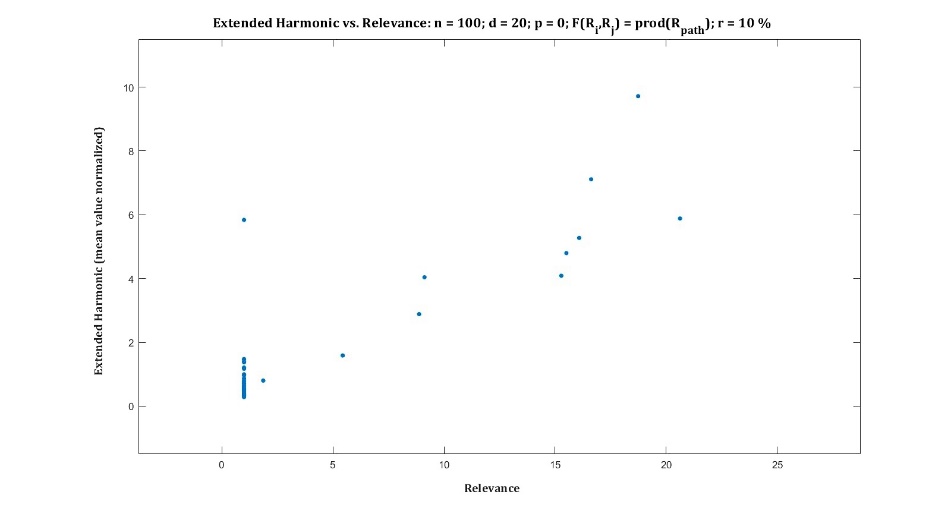

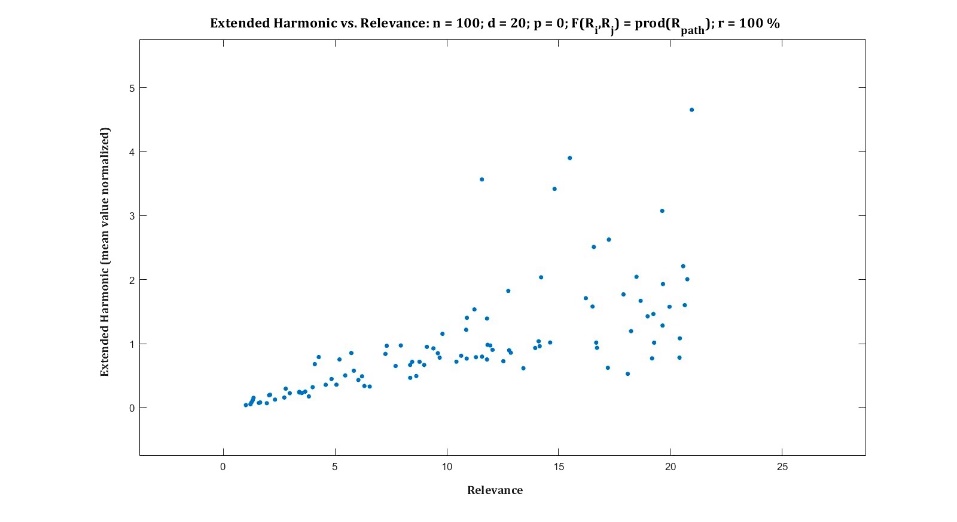


Figure 6A: *f*(*R_i_*, *R_j_*) = Π*R_path_* performed for a regular network with 100 vertices. It reports: standard *vs* relevance-based betweenness with *r*=10% (1^st^ panel-left) and *r*=100% (1^st^ panel-right), intrinsic relevance *vs* relevance-based betweenness with *r* =10% (2^nd^ panel-left) and *r* =100% (2^nd^ panel-right), classic *vs* relevance-based harmonic centrality with *r* =10% (3^rd^ panel-left) and *r* =100% (3^rd^ panel-right), and intrinsic relevance *vs* relevance-based harmonic with *r* =10% (4^th^ panel-left) and *r* =100% (4^th^ panel-right).


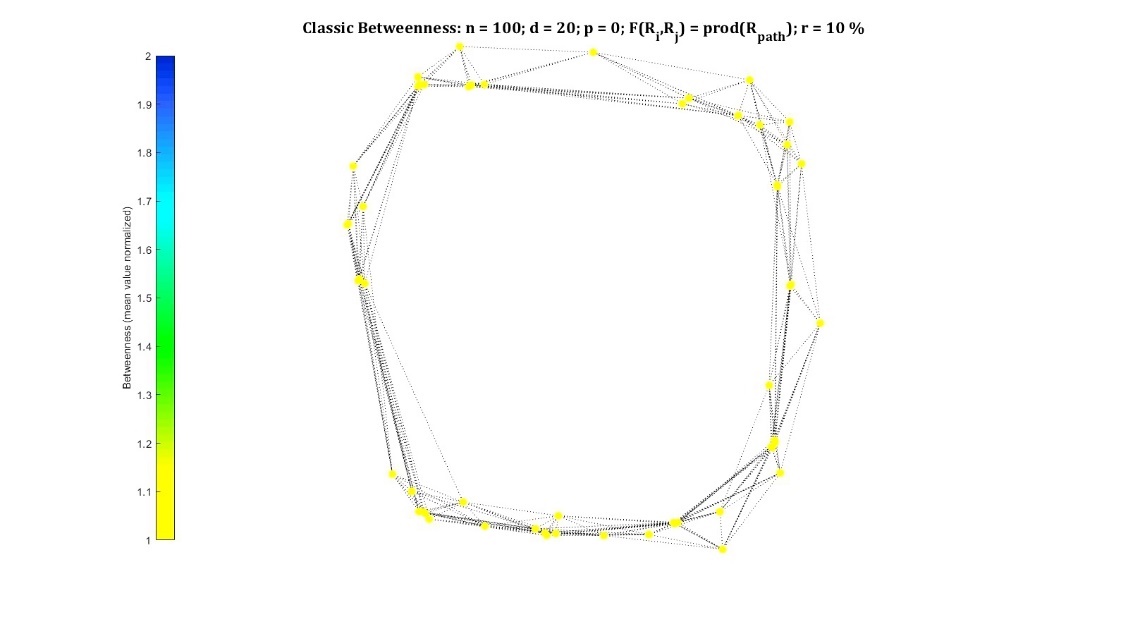

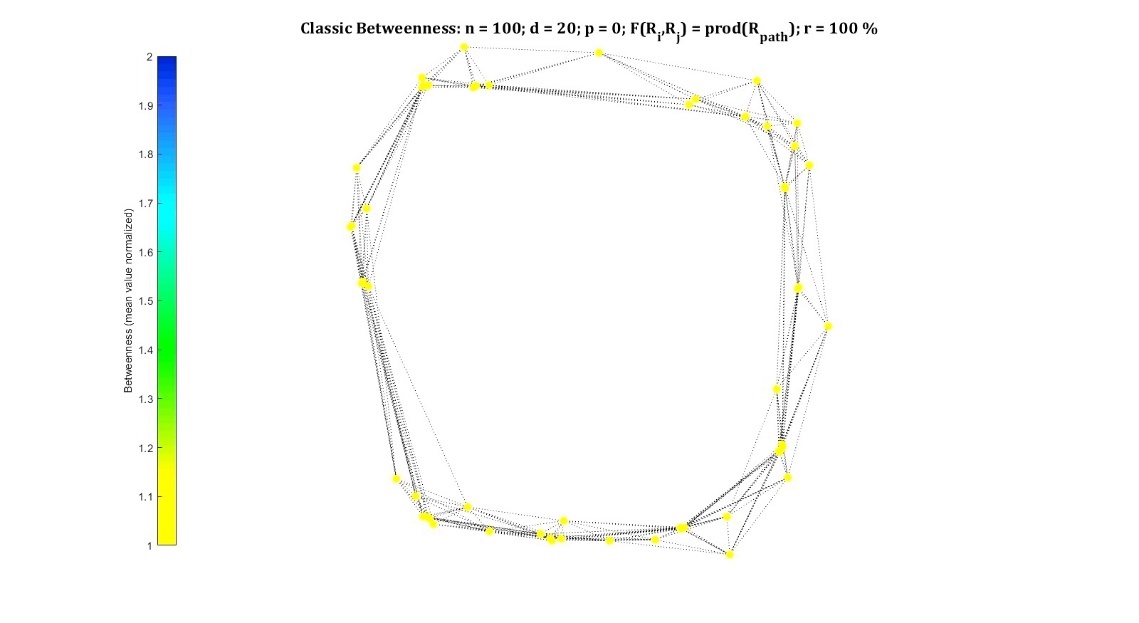


Figure 6B: regular network with 100 vertices corresponding to *r*=10% (left-panel) and *r*=100% (right-panel) of the randomly assigned intrinsic relevance. The coloured vertices refer to the colour-bar indicating the values of the: standard betweenness (1^st^ panel), relevance-based betweenness (2^nd^ panel), intrinsic relevance of vertices (3^rd^ panel), standard harmonic centrality (4^th^ panel) and relevance-based harmonic centrality (5^th^ panel). The relevance-based metrics refers to the function *f*(*R_i_*, *R_j_*) = Π*R_path_* as in Figure 6A.

Figure 7A: *f*(*R_i_*, *R_j_*) = (*R_i_*+*R_j_*)/2 performed for a random network with 100 vertices. It reports: standard *vs* relevance-based betweenness with *r*=10% (1^st^ panel-left) and *r*=100% (1^st^ panel-right), intrinsic relevance *vs* relevance-based betweenness with *r* =10% (2^nd^ panel-left) and *r* =100% (2^nd^ panel-right), classic *vs* relevance-based harmonic centrality with *r* =10% (3^rd^ panel-left) and *r* =100% (3^rd^ panel-right), and intrinsic relevance *vs* relevance-based harmonic with *r* =10% (4^th^ panel-left) and *r* =100% (4^th^ panel-right).

Figure 7B: random network with 100 vertices corresponding to *r*=10% (left-panel) and *r*=100% (right-panel) of the randomly assigned intrinsic relevance. The coloured vertices refer to the colour-bar indicating the values of the: standard betweenness (1^st^ panel), relevance-based betweenness (2^nd^ panel), intrinsic relevance of vertices (3^rd^ panel), standard harmonic centrality (4^th^ panel) and relevance-based harmonic centrality (5^th^ panel). The relevance-based metrics refers to the function *f*(*R_i_*, *R_j_*) = (*R_i_*+*R_j_*)/2 as in Figure 7A.

Figure 8A: *f*(*R_i_*, *R_j_*) = *R_i_*∙*R_j_* performed for a random network with 100 vertices. It reports: standard *vs* relevance-based betweenness with *r*=10% (1^st^ panel-left) and *r*=100% (1^st^ panel-right), intrinsic relevance *vs* relevance-based betweenness with *r* =10% (2^nd^ panel-left) and *r* =100% (2^nd^ panel-right), classic *vs* relevance-based harmonic centrality with *r* =10% (3^rd^ panel-left) and *r* =100% (3^rd^ panel-right), and intrinsic relevance *vs* relevance-based harmonic with *r* =10% (4^th^ panel-left) and *r* =100% (4^th^ panel-right).

Figure 8B: random network with 100 vertices corresponding to *r*=10% (left-panel) and *r*=100% (right-panel) of the randomly assigned intrinsic relevance. The coloured vertices refer to the colour-bar indicating the values of the: standard betweenness (1^st^ panel), relevance-based betweenness (2^nd^ panel), intrinsic relevance of vertices (3^rd^ panel), standard harmonic centrality (4^th^ panel) and relevance-based harmonic centrality (5^th^ panel). The relevance-based metrics refers to the function *f*(*R_i_*, *R_j_*) = *R_i_*∙*R_j_* as in Figure 8A.

Figure 9A: *f*(*R_i_*, *R_j_*) = max(*R_i_*, *R_j_*) performed for a random network with 100 vertices. It reports: standard *vs* relevance-based betweenness with *r*=10% (1^st^ panel-left) and *r*=100% (1^st^ panel-right), intrinsic relevance *vs* relevance-based betweenness with *r* =10% (2^nd^ panel-left) and *r* =100% (2^nd^ panel-right), classic *vs* relevance-based harmonic centrality with *r* =10% (3^rd^ panel-left) and *r* =100% (3^rd^ panel-right), and intrinsic relevance *vs* relevance-based harmonic with *r* =10% (4^th^ panel-left) and *r* =100% (4^th^ panel-right).

Figure 9B: random network with 100 vertices corresponding to *r*=10% (left-panel) and *r*=100% (right-panel) of the randomly assigned intrinsic relevance. The coloured vertices refer to the colour-bar indicating the values of the: standard betweenness (1^st^ panel), relevance-based betweenness (2^nd^ panel), intrinsic relevance of vertices (3^rd^ panel), standard harmonic centrality (4^th^ panel) and relevance-based harmonic centrality (5^th^ panel). The relevance-based metrics refers to the function *f*(*R_i_*, *R_j_*) = max(*R_i_*, *R_j_*) as in Figure 9A.

Figure 10A: *f*(*R_i_*, *R_j_*) = *R_i_* performed for a random network with 100 vertices. It reports: standard *vs* relevance-based betweenness with *r*=10% (1^st^ panel-left) and *r*=100% (1^st^ panel-right), intrinsic relevance *vs* relevance-based betweenness with *r* =10% (2^nd^ panel-left) and *r* =100% (2^nd^ panel-right), classic *vs* relevance-based harmonic centrality with *r* =10% (3^rd^ panel-left) and *r* =100% (3^rd^ panel-right), and intrinsic relevance *vs* relevance-based harmonic with *r* =10% (4^th^ panel-left) and *r* =100% (4^th^ panel-right).

Figure 10B: random network with 100 vertices corresponding to *r*=10% (left-panel) and *r*=100% (right-panel) of the randomly assigned intrinsic relevance. The coloured vertices refer to the colour-bar indicating the values of the: standard betweenness (1^st^ panel), relevance-based betweenness (2^nd^ panel), intrinsic relevance of vertices (3^rd^ panel), standard harmonic centrality (4^th^ panel) and relevance-based harmonic centrality (5^th^ panel). The relevance-based metrics refers to the function *f*(*R_i_*, *R_j_*) = *R_i_* as in Figure 10A.

Figure 11A: *f*(*R_i_*, *R_j_*) = Σ*R_path_* performed for a random network with 100 vertices. It reports: standard *vs* relevance-based betweenness with *r*=10% (1^st^ panel-left) and *r*=100% (1^st^ panel-right), intrinsic relevance *vs* relevance-based betweenness with *r* =10% (2^nd^ panel-left) and *r* =100% (2^nd^ panel-right), classic *vs* relevance-based harmonic centrality with *r* =10% (3^rd^ panel-left) and *r* =100% (3^rd^ panel-right), and intrinsic relevance *vs* relevance-based harmonic with *r* =10% (4^th^ panel-left) and *r* =100% (4^th^ panel-right).

Figure 11B: random network with 100 vertices corresponding to *r*=10% (left-panel) and *r*=100% (right-panel) of the randomly assigned intrinsic relevance. The coloured vertices refer to the colour-bar indicating the values of the: standard betweenness (1^st^ panel), relevance-based betweenness (2^nd^ panel), intrinsic relevance of vertices (3^rd^ panel), standard harmonic centrality (4^th^ panel) and relevance-based harmonic centrality (5^th^ panel). The relevance-based metrics refers to the function *f*(*R_i_*, *R_j_*) = Σ*R_path_* as in Figure 11A.

Figure 12A: *f*(*R_i_*, *R_j_*) = Π*R_path_* performed for a random network with 100 vertices. It reports: standard *vs* relevance-based betweenness with *r*=10% (1^st^ panel-left) and *r*=100% (1^st^ panel-right), intrinsic relevance *vs* relevance-based betweenness with *r* =10% (2^nd^ panel-left) and *r* =100% (2^nd^ panel-right), classic *vs* relevance-based harmonic centrality with *r* =10% (3^rd^ panel-left) and *r* =100% (3^rd^ panel-right), and intrinsic relevance *vs* relevance-based harmonic with *r* =10% (4^th^ panel-left) and *r* =100% (4^th^ panel-right).

Figure 12B: random network with 100 vertices corresponding to *r*=10% (left-panel) and *r*=100% (right-panel) of the randomly assigned intrinsic relevance. The coloured vertices refer to the colour-bar indicating the values of the: standard betweenness (1^st^ panel), relevance-based betweenness (2^nd^ panel), intrinsic relevance of vertices (3^rd^ panel), standard harmonic centrality (4^th^ panel) and relevance-based harmonic centrality (5^th^ panel). The relevance-based metrics refers to the function *f*(*R_i_*, *R_j_*) = Π*R_path_* as in Figure 12A.

Figure 13A: *f*(*R_i_*, *R_j_*) = (*R_i_*+*R_j_*)/2 performed for a regular network with 1,000 vertices. It reports: standard *vs* relevance-based betweenness with *r*=10% (1^st^ panel-left) and *r*=100% (1^st^ panel-right), intrinsic relevance *vs* relevance-based betweenness with *r* =10% (2^nd^ panel-left) and *r* =100% (2^nd^ panel-right), classic *vs* relevance-based harmonic centrality with *r* =10% (3^rd^ panel-left) and *r* =100% (3^rd^ panel-right), and intrinsic relevance *vs* relevance-based harmonic with *r* =10% (4^th^ panel-left) and *r* =100% (4^th^ panel-right).

Figure 13B: regular network with 1,000 vertices corresponding to *r*=10% (left-panel) and *r*=100% (right-panel) of the randomly assigned intrinsic relevance. The coloured vertices refer to the colour-bar indicating the values of the: standard betweenness (1^st^ panel), relevance-based betweenness (2^nd^ panel), intrinsic relevance of vertices (3^rd^ panel), standard harmonic centrality (4^th^ panel) and relevance-based harmonic centrality (5^th^ panel). The relevance-based metrics refers to the function *f*(*R_i_*, *R_j_*) = (*R_i_*+*R_j_*)/2 as in Figure 13A.

Figure 14A: *f*(*R_i_*, *R_j_*) = *R_i_*∙*R_j_* performed for a regular network with 1,000 vertices. It reports: standard *vs* relevance-based betweenness with *r*=10% (1^st^ panel-left) and *r*=100% (1^st^ panel-right), intrinsic relevance *vs* relevance-based betweenness with *r* =10% (2^nd^ panel-left) and *r* =100% (2^nd^ panel-right), classic *vs* relevance-based harmonic centrality with *r* =10% (3^rd^ panel-left) and *r* =100% (3^rd^ panel-right), and intrinsic relevance *vs* relevance-based harmonic with *r* =10% (4^th^ panel-left) and *r* =100% (4^th^ panel-right).

Figure 14B: regular network with 1,000 vertices corresponding to *r*=10% (left-panel) and *r*=100% (right-panel) of the randomly assigned intrinsic relevance. The coloured vertices refer to the colour-bar indicating the values of the: standard betweenness (1^st^ panel), relevance-based betweenness (2^nd^ panel), intrinsic relevance of vertices (3^rd^ panel), standard harmonic centrality (4^th^ panel) and relevance-based harmonic centrality (5^th^ panel). The relevance-based metrics refers to the function *f*(*R_i_*, *R_j_*) = *R_i_*∙*R_j_* as in Figure 14A.

Figure 15A: *f*(*R_i_*, *R_j_*) = max(*R_i_*, *R_j_*) performed for a regular network with 1,000 vertices. It reports: standard *vs* relevance-based betweenness with *r*=10% (1^st^ panel-left) and *r*=100% (1^st^ panel-right), intrinsic relevance *vs* relevance-based betweenness with *r* =10% (2^nd^ panel-left) and *r* =100% (2^nd^ panel-right), classic *vs* relevance-based harmonic centrality with *r* =10% (3^rd^ panel-left) and *r* =100% (3^rd^ panel-right), and intrinsic relevance *vs* relevance-based harmonic with *r* =10% (4^th^ panel-left) and *r* =100% (4^th^ panel-right).

Figure 15B: regular network with 1,000 vertices corresponding to *r*=10% (left-panel) and *r*=100% (right-panel) of the randomly assigned intrinsic relevance. The coloured vertices refer to the colour-bar indicating the values of the: standard betweenness (1^st^ panel), relevance-based betweenness (2^nd^ panel), intrinsic relevance of vertices (3^rd^ panel), standard harmonic centrality (4^th^ panel) and relevance-based harmonic centrality (5^th^ panel). The relevance-based metrics refers to the function *f*(*R_i_*, *R_j_*) = max(*R_i_*, *R_j_*) as in Figure 15A.

Figure 16A: *f*(*R_i_*, *R_j_*) = *R_i_* performed for a regular network with 1,000 vertices. It reports: standard *vs* relevance-based betweenness with *r*=10% (1^st^ panel-left) and *r*=100% (1^st^ panel-right), intrinsic relevance *vs* relevance-based betweenness with *r* =10% (2^nd^ panel-left) and *r* =100% (2^nd^ panel-right), classic *vs* relevance-based harmonic centrality with *r* =10% (3^rd^ panel-left) and *r* =100% (3^rd^ panel-right), and intrinsic relevance *vs* relevance-based harmonic with *r* =10% (4^th^ panel-left) and *r* =100% (4^th^ panel-right).

Figure 16B: regular network with 1,000 vertices corresponding to *r*=10% (left-panel) and *r*=100% (right-panel) of the randomly assigned intrinsic relevance. The coloured vertices refer to the colour-bar indicating the values of the: standard betweenness (1^st^ panel), relevance-based betweenness (2^nd^ panel), intrinsic relevance of vertices (3^rd^ panel), standard harmonic centrality (4^th^ panel) and relevance-based harmonic centrality (5^th^ panel). The relevance-based metrics refers to the function *f*(*R_i_*, *R_j_*) = *R_i_* as in Figure 16A.

Figure 17A: *f*(*R_i_*, *R_j_*) = Σ*R_path_* performed for a regular network with 1,000 vertices. It reports: standard *vs* relevance-based betweenness with *r*=10% (1^st^ panel-left) and *r*=100% (1^st^ panel-right), intrinsic relevance *vs* relevance-based betweenness with *r* =10% (2^nd^ panel-left) and *r* =100% (2^nd^ panel-right), classic *vs* relevance-based harmonic centrality with *r* =10% (3^rd^ panel-left) and *r* =100% (3^rd^ panel-right), and intrinsic relevance *vs* relevance-based harmonic with *r* =10% (4^th^ panel-left) and *r* =100% (4^th^ panel-right).

Figure 17B: regular network with 1,000 vertices corresponding to *r*=10% (left-panel) and *r*=100% (right-panel) of the randomly assigned intrinsic relevance. The coloured vertices refer to the colour-bar indicating the values of the: standard betweenness (1^st^ panel), relevance-based betweenness (2^nd^ panel), intrinsic relevance of vertices (3^rd^ panel), standard harmonic centrality (4^th^ panel) and relevance-based harmonic centrality (5^th^ panel). The relevance-based metrics refers to the function *f*(*R_i_*, *R_j_*) = Σ*R_path_* as in Figure 17A.

Figure 18A: *f*(*R_i_*, *R_j_*) = Π*R_path_* performed for a regular network with 1,000 vertices. It reports: standard *vs* relevance-based betweenness with *r*=10% (1^st^ panel-left) and *r*=100% (1^st^ panel-right), intrinsic relevance *vs* relevance-based betweenness with *r* =10% (2^nd^ panel-left) and *r* =100% (2^nd^ panel-right), classic *vs* relevance-based harmonic centrality with *r* =10% (3^rd^ panel-left) and *r* =100% (3^rd^ panel-right), and intrinsic relevance *vs* relevance-based harmonic with *r* =10% (4^th^ panel-left) and *r* =100% (4^th^ panel-right).

Figure 18B: regular network with 1,000 vertices corresponding to *r*=10% (left-panel) and *r*=100% (right-panel) of the randomly assigned intrinsic relevance. The coloured vertices refer to the colour-bar indicating the values of the: standard betweenness (1^st^ panel), relevance-based betweenness (2^nd^ panel), intrinsic relevance of vertices (3^rd^ panel), standard harmonic centrality (4^th^ panel) and relevance-based harmonic centrality (5^th^ panel). The relevance-based metrics refers to the function *f*(*R_i_*, *R_j_*) = Π*R_path_* as in Figure 18A.

Figure 19A: *f*(*R_i_*, *R_j_*) = (*R_i_*+*R_j_*)/2 performed for a random network with 1,000 vertices. It reports: standard *vs* relevance-based betweenness with *r*=10% (1^st^ panel-left) and *r*=100% (1^st^ panel-right), intrinsic relevance *vs* relevance-based betweenness with *r* =10% (2^nd^ panel-left) and *r* =100% (2^nd^ panel-right), classic *vs* relevance-based harmonic centrality with *r* =10% (3^rd^ panel-left) and *r* =100% (3^rd^ panel-right), and intrinsic relevance *vs* relevance-based harmonic with *r* =10% (4^th^ panel-left) and *r* =100% (4^th^ panel-right).

Figure 19B: random network with 1,000 vertices corresponding to *r*=10% (left-panel) and *r*=100% (right-panel) of the randomly assigned intrinsic relevance. The coloured vertices refer to the colour-bar indicating the values of the: standard betweenness (1^st^ panel), relevance-based betweenness (2^nd^ panel), intrinsic relevance of vertices (3^rd^ panel), standard harmonic centrality (4^th^ panel) and relevance-based harmonic centrality (5^th^ panel). The relevance-based metrics refers to the function *f*(*R_i_*, *R_j_*) = (*R_i_*+*R_j_*)/2 as in Figure 19A.

Figure 20A: *f*(*R_i_*, *R_j_*) = *R_i_*∙*R_j_* performed for a random network with 1,000 vertices. It reports: standard *vs* relevance-based betweenness with *r*=10% (1^st^ panel-left) and *r*=100% (1^st^ panel-right), intrinsic relevance *vs* relevance-based betweenness with *r* =10% (2^nd^ panel-left) and *r* =100% (2^nd^ panel-right), classic *vs* relevance-based harmonic centrality with *r* =10% (3^rd^ panel-left) and *r* =100% (3^rd^ panel-right), and intrinsic relevance *vs* relevance-based harmonic with *r* =10% (4^th^ panel-left) and *r* =100% (4^th^ panel-right).

Figure 20B: random network with 1,000 vertices corresponding to *r*=10% (left-panel) and *r*=100% (right-panel) of the randomly assigned intrinsic relevance. The coloured vertices refer to the colour-bar indicating the values of the: standard betweenness (1^st^ panel), relevance-based betweenness (2^nd^ panel), intrinsic relevance of vertices (3^rd^ panel), standard harmonic centrality (4^th^ panel) and relevance-based harmonic centrality (5^th^ panel). The relevance-based metrics refers to the function *f*(*R_i_*, *R_j_*) = *R_i_*∙*R_j_* as in Figure 20A.

Figure 21A: *f*(*R_i_*, *R_j_*) = max(*R_i_*, *R_j_*) performed for a random network with 1,000 vertices. It reports: standard *vs* relevance-based betweenness with *r*=10% (1^st^ panel-left) and *r*=100% (1^st^ panel-right), intrinsic relevance *vs* relevance-based betweenness with *r* =10% (2^nd^ panel-left) and *r* =100% (2^nd^ panel-right), classic *vs* relevance-based harmonic centrality with *r* =10% (3^rd^ panel-left) and *r* =100% (3^rd^ panel-right), and intrinsic relevance *vs* relevance-based harmonic with *r* =10% (4^th^ panel-left) and *r* =100% (4^th^ panel-right).

Figure 21B: random network with 1,000 vertices corresponding to *r*=10% (left-panel) and *r*=100% (right-panel) of the randomly assigned intrinsic relevance. The coloured vertices refer to the colour-bar indicating the values of the: standard betweenness (1^st^ panel), relevance-based betweenness (2^nd^ panel), intrinsic relevance of vertices (3^rd^ panel), standard harmonic centrality (4^th^ panel) and relevance-based harmonic centrality (5^th^ panel). The relevance-based metrics refers to the function *f*(*R_i_*, *R_j_*) = max(*R_i_*, *R_j_*) as in Figure 21A.

Figure 22A: *f*(*R_i_*, *R_j_*) = *R_i_* performed for a random network with 1,000 vertices. It reports: standard *vs* relevance-based betweenness with *r*=10% (1^st^ panel-left) and *r*=100% (1^st^ panel-right), intrinsic relevance *vs* relevance-based betweenness with *r* =10% (2^nd^ panel-left) and *r* =100% (2^nd^ panel-right), classic *vs* relevance-based harmonic centrality with *r* =10% (3^rd^ panel-left) and *r* =100% (3^rd^ panel-right), and intrinsic relevance *vs* relevance-based harmonic with *r* =10% (4^th^ panel-left) and *r* =100% (4^th^ panel-right).

Figure 22B: random network with 1,000 vertices corresponding to *r*=10% (left-panel) and *r*=100% (right-panel) of the randomly assigned intrinsic relevance. The coloured vertices refer to the colour-bar indicating the values of the: standard betweenness (1^st^ panel), relevance-based betweenness (2^nd^ panel), intrinsic relevance of vertices (3^rd^ panel), standard harmonic centrality (4^th^ panel) and relevance-based harmonic centrality (5^th^ panel). The relevance-based metrics refers to the function *f*(*R_i_*, *R_j_*) = *R_i_* as in Figure 22A.

Figure 23A: *f*(*R_i_*, *R_j_*) = Σ*R_path_* performed for a random network with 1,000 vertices. It reports: standard *vs* relevance-based betweenness with *r*=10% (1^st^ panel-left) and *r*=100% (1^st^ panel-right), intrinsic relevance *vs* relevance-based betweenness with *r* =10% (2^nd^ panel-left) and *r* =100% (2^nd^ panel-right), classic *vs* relevance-based harmonic centrality with *r* =10% (3^rd^ panel-left) and *r* =100% (3^rd^ panel-right), and intrinsic relevance *vs* relevance-based harmonic with *r* =10% (4^th^ panel-left) and *r* =100% (4^th^ panel-right).

Figure 23B: random network with 1,000 vertices corresponding to *r*=10% (left-panel) and *r*=100% (right-panel) of the randomly assigned intrinsic relevance. The coloured vertices refer to the colour-bar indicating the values of the: standard betweenness (1^st^ panel), relevance-based betweenness (2^nd^ panel), intrinsic relevance of vertices (3^rd^ panel), standard harmonic centrality (4^th^ panel) and relevance-based harmonic centrality (5^th^ panel). The relevance-based metrics refers to the function *f*(*R_i_*, *R_j_*) = Σ*R_path_* as in Figure 23A.

Figure 24A: *f*(*R_i_*, *R_j_*) = Π*R_path_* performed for a random network with 1,000 vertices. It reports: standard *vs* relevance-based betweenness with *r*=10% (1^st^ panel-left) and *r*=100% (1^st^ panel-right), intrinsic relevance *vs* relevance-based betweenness with *r* =10% (2^nd^ panel-left) and *r* =100% (2^nd^ panel-right), classic *vs* relevance-based harmonic centrality with *r* =10% (3^rd^ panel-left) and *r* =100% (3^rd^ panel-right), and intrinsic relevance *vs* relevance-based harmonic with *r* =10% (4^th^ panel-left) and *r* =100% (4^th^ panel-right).

Figure 24B: random network with 1,000 vertices corresponding to *r*=10% (left-panel) and *r*=100% (right-panel) of the randomly assigned intrinsic relevance. The coloured vertices refer to the colour-bar indicating the values of the: standard betweenness (1^st^ panel), relevance-based betweenness (2^nd^ panel), intrinsic relevance of vertices (3^rd^ panel), standard harmonic centrality (4^th^ panel) and relevance-based harmonic centrality (5^th^ panel). The relevance-based metrics refers to the function *f*(*R_i_*, *R_j_*) = Π*R_path_* as in Figure 24A.

**Supplementary Table S1-S9**

Tables report the values of the standard and relevance-embedding betweenness, edge betweenness, degree and harmonic centrality for Florence family’s network, standard and relevance-embedding edge betweenness for the infrastructure water supply.

Supplementary Table S1: standard betweenness for Florence family’s network. Vertices are characterized by identical intrinsic relevance. The colour bar of the corresponding figure is obtained subtracting the minimum value and normalizing to 100.

| **Family** | **Standard Betweenness Centrality** |
| --- | --- |
| Guicciardini | 0,00 |
| Tornabuoni | 0,00 |
| Medici | 24,00 |
| Da Uzzano | 0,00 |
| Rondinelli | 3,00 |
| **Guasconi** | **34,92** |
| Pepi | 0,00 |
| Scambrilla | 0,00 |
| Benizzi | 0,00 |
| Castellani | 23,00 |
| Strozzi | 26,33 |
| Rucellai | 0,00 |
| Peruzzi | 29,75 |
| Albizzi | 19,00 |
| Altoviti | 0,00 |
| Della Casa | 0,00 |
| Panciatichi | 2,00 |

Supplementary Table S2: standard edge betweenness for Florence family’s network. Vertices are characterized by identical intrinsic relevance. The colour bar of the corresponding figure is obtained subtracting the minimum value and normalizing to 100.

| **Marriage Edge** | | **Standard edge betweenness** |
| --- | --- | --- |
| Guicciardini | Medici | 8,50 |
| Tornabuoni | Medici | 8,50 |
| Medici | Guasconi | 17,50 |
| Medici | Albizzi | 7,00 |
| Da Uzzano | Guasconi | 8,33 |
| Rondinelli | Guasconi | 9,08 |
| Rondinelli | Castellani | 1,00 |
| Guasconi | Strozzi | 13,00 |
| Guasconi | Peruzzi | 4,25 |
| Guasconi | Albizzi | 1,00 |
| Pepi | Castellani | 8,00 |
| Scambrilla | castellani | 8,00 |
| Benizzi | Strozzi | 9,67 |
| **Castellani** | **Peruzzi** | **22,00** |
| Strozzi | Rucellai | 9,67 |
| Strozzi | Peruzzi | 10,00 |
| Strozzi | Panciatichi | 0,00 |
| Peruzzi | Albizzi | 3,00 |
| Peruzzi | Della Casa | 3,83 |
| Peruzzi | Panciatichi | 0,42 |
| Albizzi | Altoviti | 9,00 |
| Albizzi | Della Casa | 0,00 |
| Albizzi | Panciatichi | 2,00 |

Supplementary Table S3: relevance-embedding betweenness centrality for Florence family’s network, assuming the vertex intrinsic relevance based on gross wealth in florins. The colour bar of the corresponding figure is obtained subtracting the minimum value and normalizing to 100.

| **Family** | **Intrinsic relevance in florins** | **Relevance-embedding betweenness centrality** |
| --- | --- | --- |
| Guicciardini | 203,087 | 0,00E+00 |
| Tornabuoni | 299,878 | 0,00E+00 |
| **Medici** | 248,105 | **9,65E+11** |
| Da Uzzano | 96,131 | 0,00E+00 |
| Rondinelli | 43,588 | 3,87E+10 |
| Guasconi | 341,198 | 7,62E+11 |
| Pepi | 43,100 | 0,00E+00 |
| Scambrilla | 148 | 0,00E+00 |
| Benizzi | 26,093 | 0,00E+00 |
| Castellani | 111,355 | 8,01E+10 |
| Strozzi | 407,296 | 2,40E+11 |
| Rucellai | 93,891 | 0,00E+00 |
| Peruzzi | 150,375 | 3,69E+11 |
| Albizzi | 249,940 | 4,10E+11 |
| Altoviti | 77,621 | 0,00E+00 |
| Della Casa | 140,624 | 0,00E+00 |
| Panciatichi | 193,878 | 5,76E+10 |

Supplementary Table S4: relevance-embedding edge betweenness for Florence family’s network, assuming the vertex intrinsic relevance based on gross wealth in florins. The colour bar of the corresponding figure is obtained subtracting the minimum value and normalizing to 100.

| **Marriage Edge** | | **Relevance-embedding edge betweenness** |
| --- | --- | --- |
| Guicciardini | Medici | 4,76E+11 |
| Tornabuoni | Medici | 6,74E+11 |
| **Medici** | **Guasconi** | **8,52E+11** |
| Medici | Albizzi | 4,97E+11 |
| Da Uzzano | Guasconi | 2,26E+11 |
| Rondinelli | Guasconi | 1,35E+11 |
| Rondinelli | Castellani | 4,51E+10 |
| Guasconi | Strozzi | 6,49E+11 |
| Guasconi | Peruzzi | 2,22E+11 |
| Guasconi | Albizzi | 1,57E+11 |
| Pepi | Castellani | 8,43E+10 |
| Scambrilla | castellani | 0,00E+00 |
| Benizzi | Strozzi | 6,45E+10 |
| Castellani | Peruzzi | 2,41E+11 |
| Strozzi | Rucellai | 2,26E+11 |
| Strozzi | Peruzzi | 2,35E+11 |
| Strozzi | Panciatichi | 1,60E+11 |
| Peruzzi | Albizzi | 9,96E+10 |
| Peruzzi | Della Casa | 1,63E+11 |
| Peruzzi | Panciatichi | 1,03E+11 |
| Albizzi | Altoviti | 1,78E+11 |
| Albizzi | Della Casa | 1,51E+11 |
| Albizzi | Panciatichi | 2,66E+11 |

Supplementary Table S5: Standard degree and harmonic centrality for Florence family’s network.

| **Family** | **Degree** | **Harmonic** |
| --- | --- | --- |
| Guicciardini | 1 | 5.983 |
| Tornabuoni | 1 | 5.983 |
| Medici | 4 | 9.000 |
| Da Uzzano | 1 | 6.667 |
| Rondinelli | 2 | 7.833 |
| Guasconi | 6 | 10.667 |
| Pepi | 1 | 5.817 |
| Scambrilla | 1 | 5.817 |
| Benizzi | 1 | 6.250 |
| Castellani | 4 | 8.667 |
| Strozzi | 5 | 9.667 |
| Rucellai | 1 | 6.250 |
| Peruzzi | 6 | 10.667 |
| Albizzi | 6 | 10.333 |
| Altoviti | 1 | 6.500 |
| Della Casa | 2 | 7.667 |
| Panciatichi | 3 | 8.500 |

Supplementary Table S6: relevance-embedding degree and harmonic centrality for Florence family’s network, assuming the vertex intrinsic relevance based on gross wealth in florins. The embeds considers the intrinsic relevance of vertices in florins.

| **Family** | **Degree** | **Harmonic** |
| --- | --- | --- |
| Guicciardini | 0.50E+11 | 2,29E+11 |
| Tornabuoni | 0.74E+11 | 3,24E+11 |
| Medici | 2.71E+11 | 4,31E+11 |
| Da Uzzano | 0.32E+11 | 1,23E+11 |
| Rondinelli | 0.19E+11 | 6,08E+10 |
| Guasconi | 40.78E+11 | 6,08E+11 |
| Pepi | 0.04E+11 | 3,83E+10 |
| Scambrilla | 0.00E+00 | 1,35E+08 |
| Benizzi | 0.10E+11 | 3,26E+10 |
| Castellani | 0.26E+11 | 1,35E+11 |
| Strozzi | 32.80E+11 | 5,94E+11 |
| Rucellai | 0.38E+11 | 1,14E+11 |
| Peruzzi | 21.71E+11 | 2,90E+11 |
| Albizzi | 28.78E+11 | 4,47E+11 |
| Altoviti | 0.19E+11 | 9,43E+10 |
| Della Casa | 0.56E+11 | 1,91E+11 |
| Panciatichi | 15.65E+11 | 3,02E+11 |

Supplementary Table S7: standard edge betweenness for all pipes of the infrastructure water supply system. Source and demand nodes are characterized by the identical intrinsic relevance. The colour bar of the corresponding figure is obtained subtracting the minimum value and normalizing to 100.

| **Pipe** | **Standard edge betweenness** |
| --- | --- |
| 1 | 38 |
| 2 | 26 |
| 3 | 52 |
| **4** | **65** |
| 5 | 3 |
| 6 | 32 |
| 7 | 58 |
| 8 | 60 |
| 9 | 0 |
| 10 | 22 |
| 11 | 15 |
| 12 | 15 |
| 13 | 8 |
| 14 | 30 |
| 15 | 43 |
| 16 | 48 |
| 17 | 62 |
| 18 | 22 |
| 19 | 31 |
| 20 | 25 |
| 21 | 13 |
| 22 | 44 |
| 23 | 26 |
| 24 | 28 |
| 25 | 40 |
| 26 | 15 |
| 27 | 26 |
| 28 | 22 |
| 29 | 8 |
| 30 | 15 |
| 31 | 52 |
| 32 | 17 |
| 33 | 17 |
| 34 | 23 |

Supplementary Table S8: relevance-embedding edge betweenness of the water supply system. The intrinsic relevance of vertices is assumed equal to the demand, for nodes, and the sum of demands, for the source node. The colour bar of the corresponding figure is obtained subtracting the minimum value and normalizing to 100.

| **Pipe** | **Relevance-embedding edge betweenness** |
| --- | --- |
| 1 | 0,0305 |
| 2 | 0,0198 |
| 3 | 0,0075 |
| 4 | 0,0209 |
| 5 | 0,0044 |
| 6 | 0,0396 |
| 7 | 0,0344 |
| 8 | 0,0089 |
| 9 | 0,0000 |
| 10 | 0,0080 |
| 11 | 0,0035 |
| 12 | 0,0026 |
| 13 | 0,0007 |
| 14 | 0,0087 |
| 15 | 0,0140 |
| 16 | 0,0213 |
| 17 | 0,0279 |
| 18 | 0,0179 |
| 19 | 0,0105 |
| 20 | 0,0069 |
| 21 | 0,0040 |
| 22 | 0,0105 |
| 23 | 0,0037 |
| 24 | 0,0040 |
| 25 | 0,0060 |
| 26 | 0,0053 |
| 27 | 0,0041 |
| 28 | 0,0137 |
| 29 | 0,0050 |
| 30 | 0,0024 |
| 31 | 0,0156 |
| 32 | 0,0102 |
| 33 | 0,0027 |
| **34** | **0,0796** |

Supplementary Table S9: relevance-embedding edge betweenness of the water supply system. The intrinsic relevance of vertices is assumed equal to the demand, for nodes, and the sum of demands, for the source node. The intrinsic relevance at the vertex 10 is increased ten times due to the assumption of a hospital. The colour bar of the corresponding figure is obtained subtracting the minimum value and normalizing to 100.

| **Pipe** | **Relevance-embedding edge betweenness** |
| --- | --- |
| 1 | 0,1014 |
| 2 | 0,0887 |
| 3 | 0,0191 |
| 4 | 0,1013 |
| 5 | 0,0063 |
| 6 | 0,0565 |
| 7 | 0,0498 |
| 8 | 0,0185 |
| 9 | 0,0000 |
| 10 | 0,0875 |
| 11 | 0,0045 |
| 12 | 0,0145 |
| 13 | 0,0017 |
| 14 | 0,0197 |
| 15 | 0,0249 |
| 16 | 0,0293 |
| 17 | 0,0371 |
| 18 | 0,0282 |
| 19 | 0,0148 |
| 20 | 0,0132 |
| 21 | 0,0052 |
| 22 | 0,0170 |
| 23 | 0,0050 |
| 24 | 0,0040 |
| 25 | 0,0094 |
| 26 | 0,0068 |
| 27 | 0,0060 |
| 28 | 0,0201 |
| 29 | 0,0067 |
| 30 | 0,0041 |
| 31 | 0,0960 |
| 32 | 0,0139 |
| 33 | 0,0027 |
| **34** | **0,1710** |
